# Genetic Dissection of Cardiac Remodeling in an Isoproterenol-Induced Heart Failure Mouse Model

**DOI:** 10.1371/journal.pgen.1006038

**Published:** 2016-07-06

**Authors:** Jessica Jen-Chu Wang, Christoph Rau, Rozeta Avetisyan, Shuxun Ren, Milagros C. Romay, Gabriel Stolin, Ke Wei Gong, Yibin Wang, Aldons J. Lusis

**Affiliations:** 1 Division of Cardiology, Department of Medicine, David Geffen School of Medicine, University of California, Los Angeles, Los Angeles, California, United States of America; 2 Department of Anesthesiology, David Geffen School of Medicine, University of California, Los Angeles, Los Angeles, California, United States of America; 3 Department of Microbiology, Immunology, and Molecular Genetics, David Geffen School of Medicine, University of California, Los Angeles, Los Angeles, California, United States of America; 4 Department of Molecular, Cell, and Developmental Biology, David Geffen School of Medicine, University of California, Los Angeles, Los Angeles, California, United States of America; 5 Department of Physiology, David Geffen School of Medicine, University of California, Los Angeles, Los Angeles, California, United States of America; 6 Department of Human Genetics, David Geffen School of Medicine, University of California, Los Angeles, Los Angeles, California, United States of America; University of North Carolina, UNITED STATES

## Abstract

We aimed to understand the genetic control of cardiac remodeling using an isoproterenol-induced heart failure model in mice, which allowed control of confounding factors in an experimental setting. We characterized the changes in cardiac structure and function in response to chronic isoproterenol infusion using echocardiography in a panel of 104 inbred mouse strains. We showed that cardiac structure and function, whether under normal or stress conditions, has a strong genetic component, with heritability estimates of left ventricular mass between 61% and 81%. Association analyses of cardiac remodeling traits, corrected for population structure, body size and heart rate, revealed 17 genome-wide significant loci, including several loci containing previously implicated genes. Cardiac tissue gene expression profiling, expression quantitative trait loci, expression-phenotype correlation, and coding sequence variation analyses were performed to prioritize candidate genes and to generate hypotheses for downstream mechanistic studies. Using this approach, we have validated a novel gene, *Myh14*, as a negative regulator of ISO-induced left ventricular mass hypertrophy in an *in vivo* mouse model and demonstrated the up-regulation of immediate early gene *Myc*, fetal gene *Nppb*, and fibrosis gene *Lgals3* in ISO-treated *Myh14* deficient hearts compared to controls.

## Author Summary

Heart failure is the most common cause of morbidity and mortality in the aging population. Previous large-scale human genome-wide association studies have yielded only a handful of genetic loci contributing to heart failure-related traits. Using a panel of diverse inbred mouse strains, treated with a β-adrenergic agonist isoproterenol to mimic the heart failure state, we sought to uncover the contribution of common genetic variation in heart failure. We found that heart failure has a strong genetic component. We successfully identified 17 genome-wide significant loci associated with indices of heart failure. We showed that genetic variation in a novel gene *Myh14* affects heart failure by altering the mechanical responses of heart muscles to isoproterenol-induced stress. Follow-up studies of this gene and additional candidate genes and loci should reveal potential mechanisms by which genetic variations contribute to heart failure in the general human population. Such insights may lead to improved diagnosis and tailor treatment based on the genetic makeup of individuals in the population.

## Introduction

Heart failure (HF) is a major health issue, affecting 5.7 million people in the United States [1]. Despite advances in therapy, HF remains a lethal condition with 5- and 10-year mortality rates of greater than 40% and 60% [2]. A number of etiologic factors, such as coronary artery disease, hypertension, valvular disease, alcohol, chemotherapy, and rare deleterious genetic mutations can lead to cardiac injury that results in HF but little is known about how common genetic variants contribute to HF progression. Irrespective of the primary insult, compensatory adrenergic and renin-angiotensin activation augment heart rate (HR), contractility and fluid retention to maintain adequate cardiac output and preserve organ function, which in turn leads to chronic maladaptive cellular growth and irreversible myocardial injury, furthering HF progression [3].

Such molecular, cellular and extracellular changes, manifested clinically as changes in size, shape and function of the heart, is also known as cardiac remodeling and is one of the most important clinical determinants of HF progression. In addition, β-adrenergic receptor blockers and angiotensin-converting enzyme (ACE) inhibitors, HF therapeutic agents that provide morbidity and mortality benefits, reverse ventricular dilation and systolic dysfunction, further supporting the importance of clinical cardiac remodeling as HF therapeutic targets [4,5]. Understanding how common genetic variation modifies the pathophysiology of HF progression in terms of cardiac remodeling will likely provide insights in the design of novel therapeutics to improve survival and life quality of HF patients.

The unbiased genome-wide association study (GWAS) design is well suited to detect the effects of genetic variation on complex traits such as HF [6]. However, a number of human HF GWAS performed to-date have had limited success. For example, a study on cardiac structure and function yielded one significant locus explaining <1% of variance in left ventricular diastolic diameter (LVIDd) [7]. A meta-analysis of 4 community-based GWAS cohorts, involving nearly 24,000 subjects, identified only two loci to be significantly associated with incident HF, explaining a very small fraction of the variance [8]. A sporadic dilated cardiomyopathy GWAS, involving 1179 cases and 1108 controls from several European populations, identified only two associated loci [9]. Lastly, a GWAS of cardiac structure and function in 6,765 African Americans identified 4 loci associated with left ventricular mass (LVM), interventricular septal wall thickness (IVSd), LVIDd, and ejection fraction (EF) [10]. In spite of their scales, the paucity of detailed phenotypic data as well as environmental heterogeneity hampered detection of genetic signals driving HF in these large human cohorts.

The laboratory mouse, with its fully sequenced and annotated genome, targeted germ line modification and easy accessibility, provides a means of overcoming some of the challenges in human studies and is complementary. Particularly informative have been reverse genetic studies of candidate genes and pathways utilizing engineered mouse models. Studies of natural variation of mice and rats, some involving sensitized models, have also resulted in the identification of novel pathways contributing to HF and other common cardiovascular traits [11,12,13,14,15]. A major difficulty of the latter, however, has been the poor resolution of classical linkage studies, making the identification of underlying causal genes a laborious process.

Recently, with the development of high-density genotyping and sequencing in rodents, relatively high-resolution association mapping approaches, analogous to human GWAS, have become feasible [16]. Our group has pioneered a resource termed the Hybrid Mouse Diversity Panel (HMDP), a panel of 100+ strains of inbred mice that have either been sequenced or densely genotyped and display natural inter-strain genetic variation, allowing a mapping resolution more than an order of magnitude higher than traditional crosses [17]. The method combines the use of classic inbred strains for mapping resolution and recombinant inbred (RI) strains for power and has been used to successfully identify a number of genes and loci involved in lipid, obesity, bone, and behavioral traits [16,18,19,20]. Because the HMDP strains are renewable, the resource is well suited to the application of systems genetics approaches, involving the integration of high throughput molecular phenotypes, such as expression array data, with clinical phenotypes.

We have previously reported a genetic analysis of β-adrenergic agonist isoproterenol (ISO)-induced cardiac hypertrophy in the HMDP, identifying a number of loci contributing to heart weight and fibrosis [21]. We now extend this study to examine ISO-induced cardiac remodeling, i.e. cardiac structural and functional changes as characterized by serial echocardiography, a powerful and noninvasive tool to serially monitor cardiac structure and function in murine injury models [22,23]. We demonstrate fine phenotypic data across the concentric-eccentric and functional cardiac remodeling spectra and found that cardiac remodeling traits among the HMDP are highly heritable. Using a common genetic variation association method, we identify 17 genome-wide significant loci with high resolution that modify ISO-induced cardiac remodeling in the heart, many of which are less than 1 Mb in size. Then, we utilize expression arrays from left ventricular (LV) tissues, with and without ISO treatment, to understand the genetic control of gene expression and gene expression correlation to cardiac remodeling phenotypes to further prioritize candidate genes in each locus. Finally, using *in vitro* and *in vivo* models, we validate a novel gene *Myh14* as causative in ISO-induced cardiac remodeling not previously reported in our heart weight analyses [21].

## Materials and Methods

### Ethics statement

All animal experiments were conducted following guidelines established and approved by the University of California, Los Angeles Institutional Animal Care and Use Committee. Induction and maintenance dosages of isoflurane was used to ensure adequate sedation while minimizing the effects of inhaled isoflurane on loading conditions, heart rate, cardiac structure and function during echocardiogram. At the end of the protocol, mice were sacrificed by sub-lethal dosage of isoflurane anesthesia followed by cervical dislocation.

### Chronic β-adrenergic stimulation, echocardiography, and tissue collection from the HMDP

#### HMDP mice

Breeding pairs from the HMDP inbred strains were obtained from the Jackson Laboratory (Bar Harbor, ME, USA). Eight- to ten-week-old nulliparous female offspring from the following 104 mouse strains were used, including 30 classical inbred strains (129X1/SvJ, A/J, AKR/J, BALB/cByJ, BALB/cJ, BTBRT<+>tf/J, BUB/BnJ, C3H/HeJ, C57BL/6J, C57BLKS/J, C57L/J, C58/J, CBA/J, CE/J, DBA/2J, FVB/NJ, KK/HlJ, LG/J, LP/J, MA/MyJ, NOD/LtJ, NON/LtJ, NZB/BlNJ, NZW/LacJ, PL/J, RIIIS/J, SEA/GnJ, SJL/J, SM/J, SWR/J) and 74 recombinant inbred (RI) strains [RI (number of strains)–AXB (8), BXA (10), BXD (44), BXH(5), CXB (7)]. All animal experiments were conducted following guidelines established and approved by the University of California, Los Angeles Institutional Animal Care and Use Committee.

#### Chronic β-adrenergic stimulation and tissue collection

Using i.p. ketamine as a surgical anesthetic agent, ALZET Model 1004 minipumps (Cupertino, CA, USA) were implanted intra-peritoneally to administered ISO, a non-selective β-adrenergic agonist that has been used widely to mimic the HF state in experimental animals [24,25], at a dose of 30 mg/kg body weight/day for 21 days. Approximately four ISO-treated (S5 Table) and 2–4 control ([21] S1 Table) mice from each strain were examined. At the end of the protocol, mice were sacrificed by giving a sub-lethal dosage of inhaled isoflurane followed by cervical dislocation. LV tissues were collected and frozen immediately in liquid nitrogen.

#### Echocardiography

To ensure adequate sedation while minimizing the effects of inhaled isoflurane on loading conditions, HR, cardiac structure and function, we minimized induction and maintenance doses of isoflurane at or below 1.25% and 1%, respectively, while closely monitoring for HR < 475 bpm as a sign for deep sedation and adjusting isoflurane dosage as needed [26].

A single operator (JJW), who followed a standard operating protocol detailed below, performed all of the echocardiograms using the Vevo 770 ultrasound system (VisualSonics, Inc., Toronto, ON, Canada): A parasternal long-axis B-mode image was obtained. The maximal long-axis of the LV was positioned perpendicular to the ultrasound beam. A 90° rotation of the ultrasound probe at the papillary muscle level was performed to obtain a parasternal short-axis view of the LV. A M-mode image to document LV dimensions was captured and saved for analysis. At a later time, saved images were analyzed using the Vevo 770 cardiac analysis package by a single observer (JJW) who was blinded to mouse strains.

A baseline echocardiogram was performed on all the mice. In ISO-treated mice, serial echocardiograms were performed at 1, 2, and 3 weeks of treatment. In control mice, a second echocardiogram was performed in 70 mouse strains at week 3 as an internal control. The reproducibility of our echocardiographic measurements was assessed using the Bland-Altman plots, which demonstrated acceptable agreement between week 0 and week 3 control measurements (S1B Fig). IVSd and LVM were not statistically different between the 2 time points, although fractional shortening (FS) was 2.3% higher and LVIDd 0.07 mm lower at week 3 compared to baseline (p = 0.002 and 0.019, respectively), possibly due to the effects of co-housing with ISO-treated animals (S1 Table).

### Transcriptome profiling and analysis

#### RNA extraction

Frozen LV tissues were homogenized in QIAzol Lysis Reagent prior to RNA isolation using RNeasy columns (QIAGEN, Valencia, CA, USA). RNA quality was assessed using the Bioanalyzer RNA kits (Agilent Technologies, Santa Clara, CA, USA). RIN ≥ 7.0 was considered acceptable.

#### Expression array profiling

Expression profiling of pooled biological replicates was performed using Illumina MouseRef-8 v2.0 Expression BeadChip arrays (Illumina, Inc., San Diego, CA, USA). To minimize artifacts due to single nucleotide polymorphisms (SNPs), we excluded probes that aligned to sequences containing known SNPs. Background correction and quantile normalization of the image data was performed using the neqc method from the R package limma [27]. Hierarchical clustering of samples was performed to exclude outlier samples using the R package WGCNA [28]. In total, expression profiles for 90 control and 91 ISO strains (including 82 strains with matching control and isoproterenol samples) were included in downstream analyses.

#### Differential gene expression

Using the R package limma, moderated t-statistics and the associated p values were calculated. Multiple testing was corrected by controlling for false discovery rate using the Benjamini-Hochberg procedure [29]. Probes with log2-fold change > 0.2 and adjusted p-value < 0.05 were considered significantly differentially expressed. Functional analysis of probe lists was performed using the Database for Annotation, Visualization and Integrated Discovery (DAVID) [30] to identify pathways and cellular processes enriched in genes differentially regulated by ISO stimulation [30,31]. In total 1,502 of the 18,335 probes (8.2%) were differentially expressed at > 15% change at an adjusted p-value < 0.05, including 840 up-regulated and 662 down-regulated probes (S2A Table). DAVID gene ontology analysis was performed on the differentially expressed probes as well as the top 1000 correlated transcripts for each phenotype-expression pair (S2B–S2D Table).

### Statistical analyses, heritability estimation, association mapping and candidate prioritization

#### Statistical analysis and graphical display

The standard R program was used to performed t-test. The R package vioplot was used to plot violin plots. The R package corrplot was used to plot correlations among traits. Consistent with time series data, serial measurements of the same trait were generally correlated to each other with adjacent time points being the most correlated. Of note, LVM was a calculated measure based in part on IVSd and LVIDd; therefore, it is not surprising that LVM was correlated with IVSd and LVIDd at each time point. The R package FactoMineR was used to compute principal component analysis and display variables factor map and individuals factor map. The R package WGCNA was used to calculate biweight midcorrelation (bicor), which is a median based correlation measure that is more robust than the Pearson correlation but often more powerful than the Spearman correlation. The proportion of variance (ω^2^) explained was computed from the formula:

$$\omega^{2}=\frac{SSQ_{condition}-(k-1)MSE}{SSQ_{total}+MSE}$$ (1)

where MSE is the mean square error and k is the number of conditions.

#### Genotypes

The HMDP mouse strains were previously genotyped using the JAX Mouse Diveristy Genotyping Array [32,33]. To select for informative and high quality SNPs, each SNP was filtered for > 5% minor allele frequency and < 10% missing values among the strains using plink [34]. Approximately 210,000 of the informative SNPs were selected.

#### Heritability calculation

Heritability is the proportion of observed differences due to genetic variation in the population. Specifically, broad-sense heritability (H2) reflects all the genetic contributions to a population's phenotypic variance including additive, dominant, and epistatic effects, while narrow-sense heritability (h2) represents the proportion of phenotypic variance that is due to additive genetic effects alone. In inbred model organisms, repeatability expresses the proportion of variation in a trait that is due to differences between individual strains and not due to differences within individual strains. Under the assumption that all differences between genotypes are genetic and not due to genotype-environment correlation, repeatability is equal to broad-sense heritability; otherwise it only provides an upper-bound for broad-sense heritability. Using the R package heritability [35], an analysis of variance (ANOVA)–based method that accounts for differing numbers of replicates, called line repeatability, was used to estimate H2 for each phenotype and time point. In addition, a linear model method incorporating genetic relatedness matrix and within-strain variability, called marker-based h2, was used to compute h2 and confidence intervals.

#### Genome-wide significance threshold

The threshold for genome-wide significance was determined by simulation and permutation of strain genotypes, as previously described [19]. The significance threshold for clinical traits was determined to be 4.1 x 10^−6^. The significance threshold for cis-eQTL and trans-eQTL were 3.63 x 10^−3^ and 6.1 x 10^−5^, respectively. Given that the studied traits could be collapsed to roughly a handful of PCs, the significance threshold for clinical trait analyses was kept at the nominal p-value of 4.1 x 10^−6^, without Bonferroni adjustment for multiple testing.

#### Association mapping

We used the Factored Spectrally Transformed Linear Mixed Models (FaST-LMM) to test for association while accounting for population structure and genetic relatedness among strains as previously described [36], which produced the same results as efficient mixed-model association (EMMA) but with a run time and memory footprint that is only linear to the cohort size (S2 Fig). Association mapping was performed for echocardiographic measurements at each time point and the change in measurements from baseline at each time point while adjusting for baseline body weight (BBW) and HR to define clinical quantitative trait loci (cQTL). In addition, association mapping for gene expression traits, under baseline and isoproterenol-treated conditions, was performed to define expression quantitative trait loci (eQTL). As a general rule, upwards of 90 to 100 strains are required to ensure stable mapping results.

#### Linkage disequilibrium block boundaries

Linkage disequilibrium (LD) is the non-random association of alleles generally near one another at a locus. SNPs in strong LD tend to have association p-values that are similar in strength. Genomic boundaries around lead association SNPs were chosen based on flanking SNPs with p-values < 1 x 10^−5^ that were no more than 2 Mb apart between nearest consecutive pairs. Lead SNPs without any neighboring SNPs at p-value < 1 x 10^−5^ were excluded. LD between lead SNPs and flanking SNPs were calculated and visualized by plotting regional plots using a custom-built of LocusZoom [37] based on our HMDP mice.

#### Candidate gene prioritization

Genes within LD (r^2^ > 0.8) of the lead SNPs were examined for coding sequence and splice region variations using the Wellcome Trust Mouse Genomes Project (MGP) sequencing database [38] using a vcftools-based pipeline. SIFT score, a functional annotation score for non-synonymous variants, was noted whenever available [39]. The expression profiles of genes within LD or nearby the lead SNPs were further examined for the presence of cis-expression quantitative trait loci (cis-eQTL). When a transcript’s cis-eQTL coincides with the clinical trait locus and is correlated with the trait, a causal relationship between the locus, the transcript and the trait may be inferred.

### *Myh14* functional validation in NRVM and in knock-out mice

#### siRNA knock-down neonatal rat ventricular myocytes

Neonatal rat ventricular myocytes (NRVMs) were isolated and cultured at the UCLA NRVM core facility using 2–4 day old rats. Myocytes and fibroblasts were separated using Percoll density gradient. NRVMs were plated at 375,000 cells/well in DMEM + 10% FBS + 100 U/mL penicillin + 100 μg/mL streptomycin (p/s) on Day 1. On the morning of Day 2, plated NRVMs were transfected with 30 pmol per well of siRNA (siMyh14#1—#RNC.RNAI.N001100690.12.1, siMyh14#2—RNC.RNAI.N001100690.12.6., or scrambled control purchased from IDT) using Lipofectamine 2000 (Life Technologies) and media changed to DMEM + 1% ITS (Insulin, Transferrium and Selenium) without p/s for *Myh14* knockdown experiments. On Day 3, at 24 hours following transfection, cells were treated with 20 μM ISO or 50 μM phenylephrine (PE) for an additional 24 hours prior to bright field imaging on day 4.

MTT Assay was performed using the Vybrant MTT Cell Proliferation Assay Kit (Life Technologies V13154). Briefly, NRVMs were plated in 96 well plates at a density of 10,000 cells per well and were transfected with either scrambled control or *Myh14* siRNA using Lipofectamine RNAimax overnight. Cells were then treated the next day with 20 μM ISO or 50 μM PE for 24 hours and then viability was assayed using MTT.

#### Myh14 knock-out mice

*Myh14*^-/-^ mice generated on a 129/Sv background followed by backcrossing to strain C57BL/6 for more than 7 generations were obtained from the Adelstein lab [40] and backcrossed (*Myh14*^-/-^ male x C57BL/6J female) to generate *Myh14*^+/-^ mice. *Myh14*^+/-^ heterozygous crosses generated wild-type (WT), heterozygous (HET) and homozygous (KO) littermates at normal mendelian ratios (Chi-Square test p = 0.13, S3 Table). Six WT, nine HET, and seven KO twelve-week-old nulliparous female mice were subjected to chronic β-adrenergic stimulation and echocardiogram in the same manner as in the HMDP described above.

#### Histology and immunofluorescence microscopy

Hearts were fixed in 10% paraformaldehyde in PBS. Masson’s trichrome staining and immunofluorescence staining were performed as previously described [40]. The antibodies used for immunofluorescence staining were N-cadherin (mouse, 1:2000, Invitrogen), β-catenin (mouse, 1:1000, Zymed), and wheat germ agglutinin (WGA). Paraffin embedded heart sections were first dewaxed and rehydrated by standard procedures. Following antigen retrieval in citric acid buffer (10 mM, pH 6.0), the sections were blocked with 1% BSA/5% goat serum in PBS for 1 hour at room temperature and then incubated with Alexa Fluor 594-WGA (10 μg/ml, Invitrogen) and DAPI in blocking solution for 1 hour at room temperature. The slides were then washed and mounted using Prolong Anti-fade mount media (Invitrogen).

### Accession numbers

Expression array data are deposited in the Gene Expression Omnibus 19 (GEO) online database http://www.ncbi.nlm.nih.gov/geo/ (Accession GSE48760).

## Results

### Experimental design and quality control

**Fig 1:**
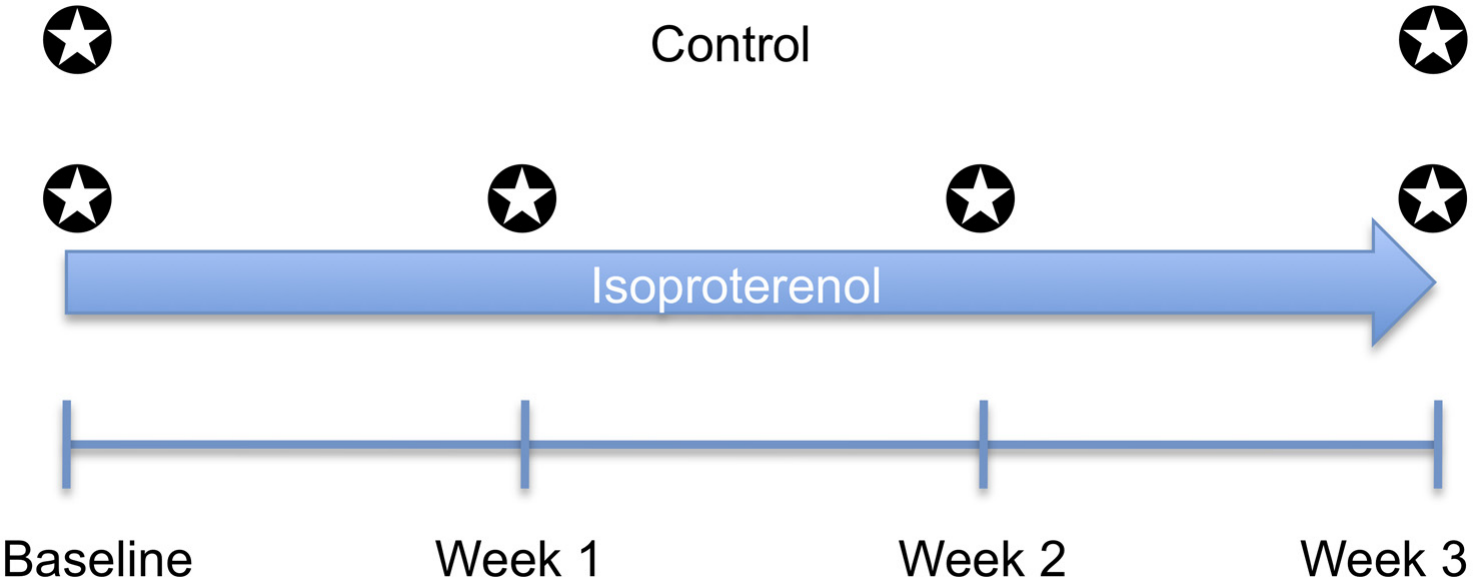
Experimental design. We characterized 104 classical and recombinant inbred mouse strains of the Hybrid Mouse Diversity Panel (HMDP) by serial echocardiograms at baseline, week 1, week 2, and week 3 under the control condition or chronic isoproterenol (ISO) infusion. The ✪ symbol indicates the time points echocardiograms were performed.

We characterized 104 classical and RI mouse strains by obtaining echocardiographic measurements under the baseline condition and in response to chronic ISO administration for 3 weeks (Fig 1). Global gene expression profiling of LV tissues from control and ISO-treated mice was performed to identify genes whose expression was correlated to HF traits and to identify eQTLs to prioritize candidate genes. We previously reported association mapping of heart weight and fibrosis [21]; the present study focuses on echocardiographic measures of clinical significance, including IVSd, LVIDd, LVM and FS, to enable mapping of fine cardiac remodeling phenotypes due to ISO-treatment.

To minimize inter-operator and inter-observer variation, all echocardiograms were performed by a single operator and interpreted by the same observer who was blinded to strain name and treatment assignment. Inhaled isoflurane was titrated to achieve adequate sedation, while maintaining target HR above 475 bpm. The Bland-Altman plot, which shows the agreement between two quantitative methods, demonstrated acceptable differences between ISO-treated LVM (a calculated value based on echo parameters) and LV weight (a weighed value after LV was dissected from the atria and RV) estimates (S3A–S3C Fig). LVM by echocardiogram was significantly correlated to LV weight at sacrifice (r = 0.77 in control and r = 0.85 in ISO both with p-value < 2.2 x 10^−16^), validating our echocardiographic measures externally.

### The HMDP exhibits significant variation in cardiac structure and function both under baseline and ISO-treated conditions

**Fig 2:**
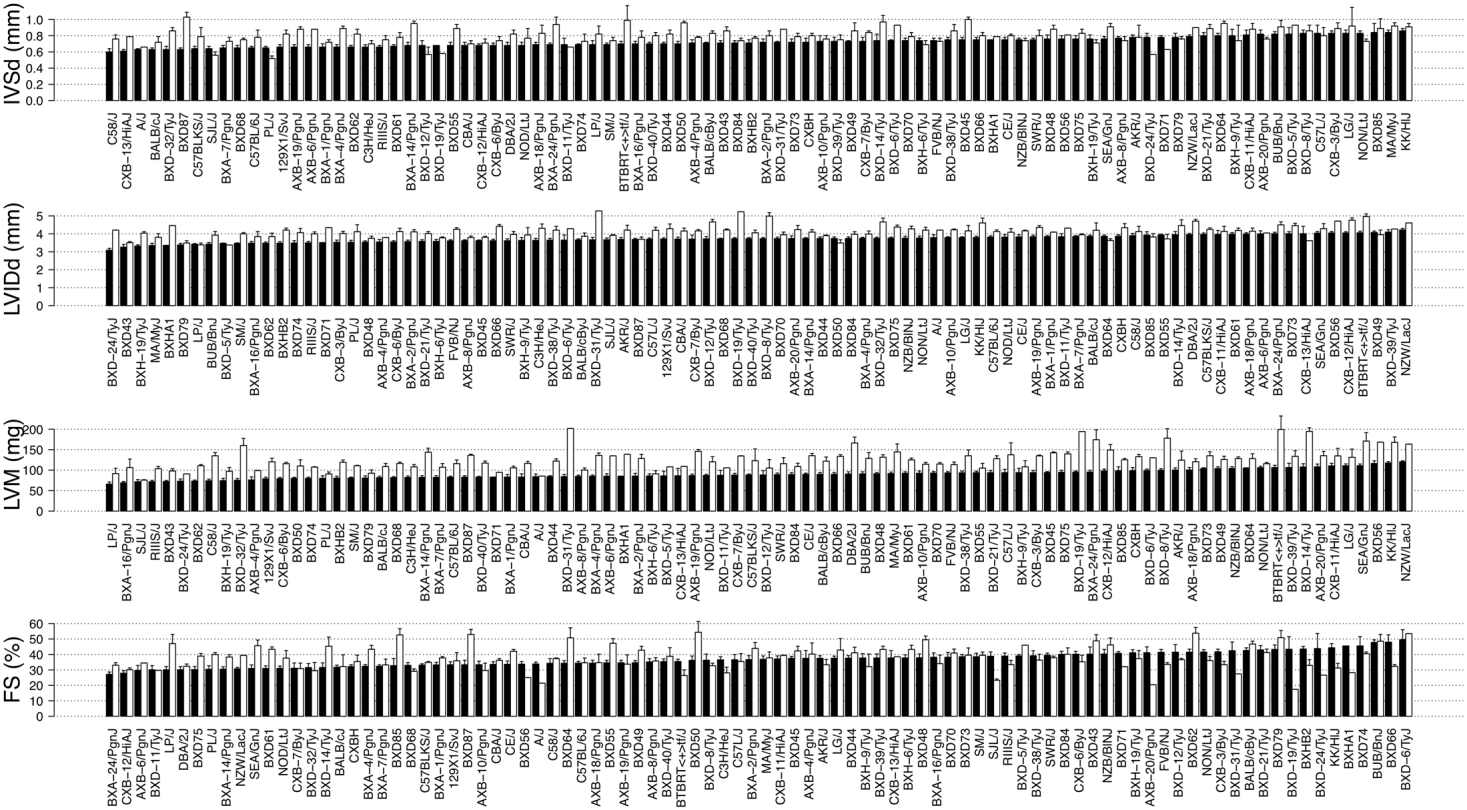
Variation in echocardiographic measures of cardiac structure and function among HMDP mouse strains. Black bars represent measurements under the baseline condition in ranked order. White bars represent measurements after 3 weeks of continuous ISO infusion. IVSd = interventricular septal wall thickness; LVIDd = left ventricular diastolic diameter; LVM = left ventricular mass; FS = fractional shortening. Error bars represent the standard errors of the means.

**Fig 3:**
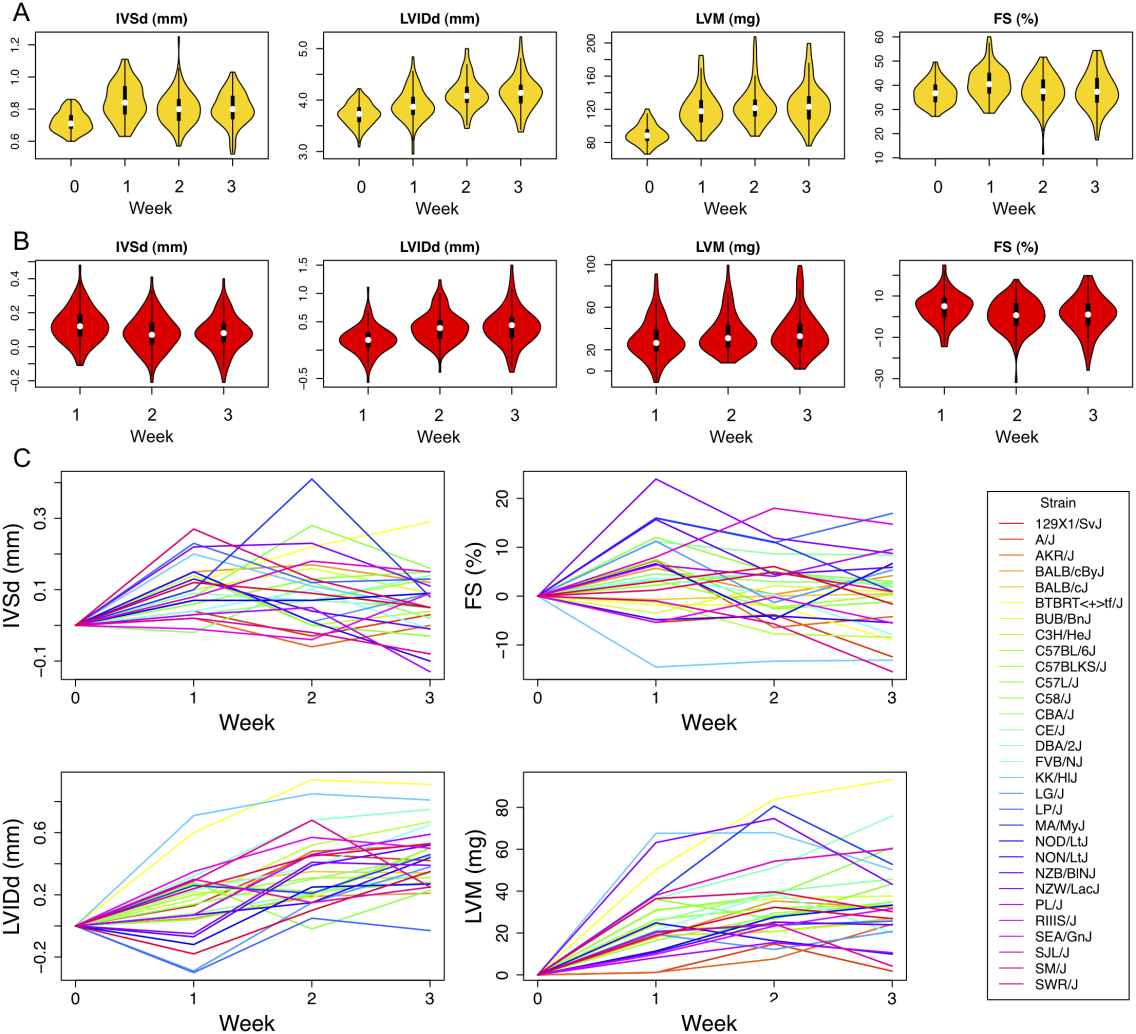
Population distribution of echocardiographic measures at each time point. The violin plot is a combination of a boxplot and a kernel density plot (a smooth histogram) rotated on its side. The white dot represents the median. The black box represents the interquartile range (IQR). The black vertical line represents the whiskers spanning the lowest and the highest data within 1.5 IQR from the lower and upper quartile. A) The population distribution at each ISO treatment time point. B) The distribution of the changes in echocardiographic measures from baseline. C) The changes in echocardiographic measures compared to baseline at each ISO time point for individual classical inbred strains.

We observed striking variation in cardiac structure and function among the HMDP strains both under baseline condition and in response to chronic ISO administration (Fig 2 and S4 Fig). Importantly, the phenotypic spectrum among the BXD RI lines exceeded that of their parental strains C57BL/6 and DBA/2, consistent with cardiac remodeling being a complex trait involving multiple genes (S5 Fig). Of note, the phenotypic spectra on ISO treatment were significantly broader than baseline, consistent with ISO perturbation enhancing the phenotypic variation among HMDP strains (Fig 3A and 3B). Moreover, across the HMDP strains, IVSd and FS increased significantly at week 1 and attenuated at later time points, reflecting the acute and chronic effects of ISO on septal wall hypertrophy and cardiac inotropy. LVIDd and LVM progressively increased with ISO treatment at each time point, reflecting progressive changes in LV chamber dimension and LV mass due to ISO (S4 Table). While many of the strains follow the general population trend described above, strains particularly susceptible to adverse cardiac remodeling, such as KK/HlJ and BTBRT<+>tf/J, demonstrated significantly decreased FS and increased in LVIDd (Fig 3C). The observed phenotypic variation, especially upon ISO treatment, underpins the basis for association mapping. The strain-level phenotype data are provided in S5 Table for reference.

### The HMDP demonstrates the presence of a compensatory-decompensating phenotypic spectrum

We performed correlation analysis across the HMDP to further understand the phenotype data observed. Not surprisingly, baseline body weight (BBW), a surrogate for body size, was significantly correlated with LVM and LVIDd but not with FS (S6A Fig), consistent with larger mice having larger and heavier hearts but body size not being a determinant of systolic heart function. In addition, under the baseline and ISO-treated conditions, HR was positively correlated with FS and negatively correlated with LVIDd. This observation is consistent with known negative chronotropic and inotropic effects of isoflurane on the heart and reflects the varying degrees of isoflurane sensitivity among HMDP strain. In fact, HR was significantly correlated across control and different ISO-treated time points, indicative of a strain-specific HR response to ISO. (S6B and S6C Fig). Interestingly, HR had no significant correlation with LVM estimates. Based on these findings, we chose to correct for confounding effects of body size and isoflurane sensitivity using BBW and HR as surrogate markers in downstream analyses.

**Fig 4:**
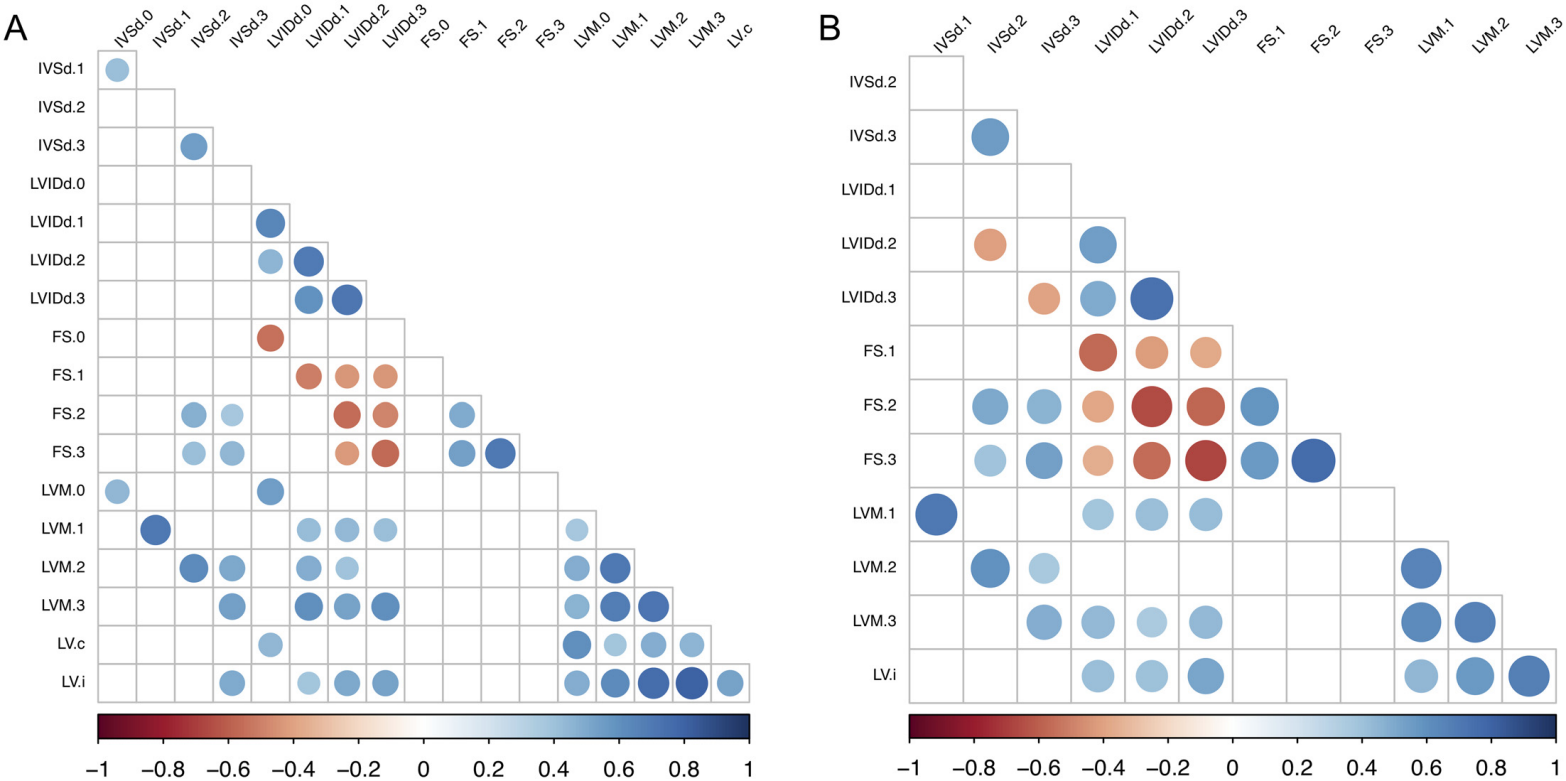
Correlations among echocardiographic traits across time points. A) Correlations among baseline body weight- and heart rate-adjusted traits across time points. B) Correlations among isoproterenol-induced changes in traits from baseline across time points. Colored dots represent the pairwise correlation r values with p-values exceeding Bonferroni corrected α significance level of 0.05. LV represents LV weight. Suffixes represent weekly echocardiographic time points under ISO treatment (0 = baseline, 1 = week 1, 2 = week 2, 3 = week 3) or control versus ISO LV weight at week 3 (c = control, i = ISO).

BBW- and HR-adjusted partial correlations among pairs of echocardiographic and LV weight traits showed that ISO treatment uncoupled many measures of cardiac structure and function from baseline (Fig 4A). ISO-treatment introduced an immediate perturbation on FS, such that FS from week 1 onwards was uncorrelated to FS at baseline, possibly due to different genetic factors controlling FS before and after ISO. In fact, FS at week 1 was negatively correlated with LVIDd at later time points, consistent with week 1 FS being a measure of contractile reserve and an early predictor of progressive ventricular dilation. In contrast, ISO-induced perturbation on IVSd was not observed until week 2. IVSd hypertrophy was positively correlated with increased FS and negatively correlated with LVIDd dilation, consistent with the presence of a compensatory-decompensating spectrum that is genetically controlled and required for association mapping (Fig 4B).

**Fig 5:**
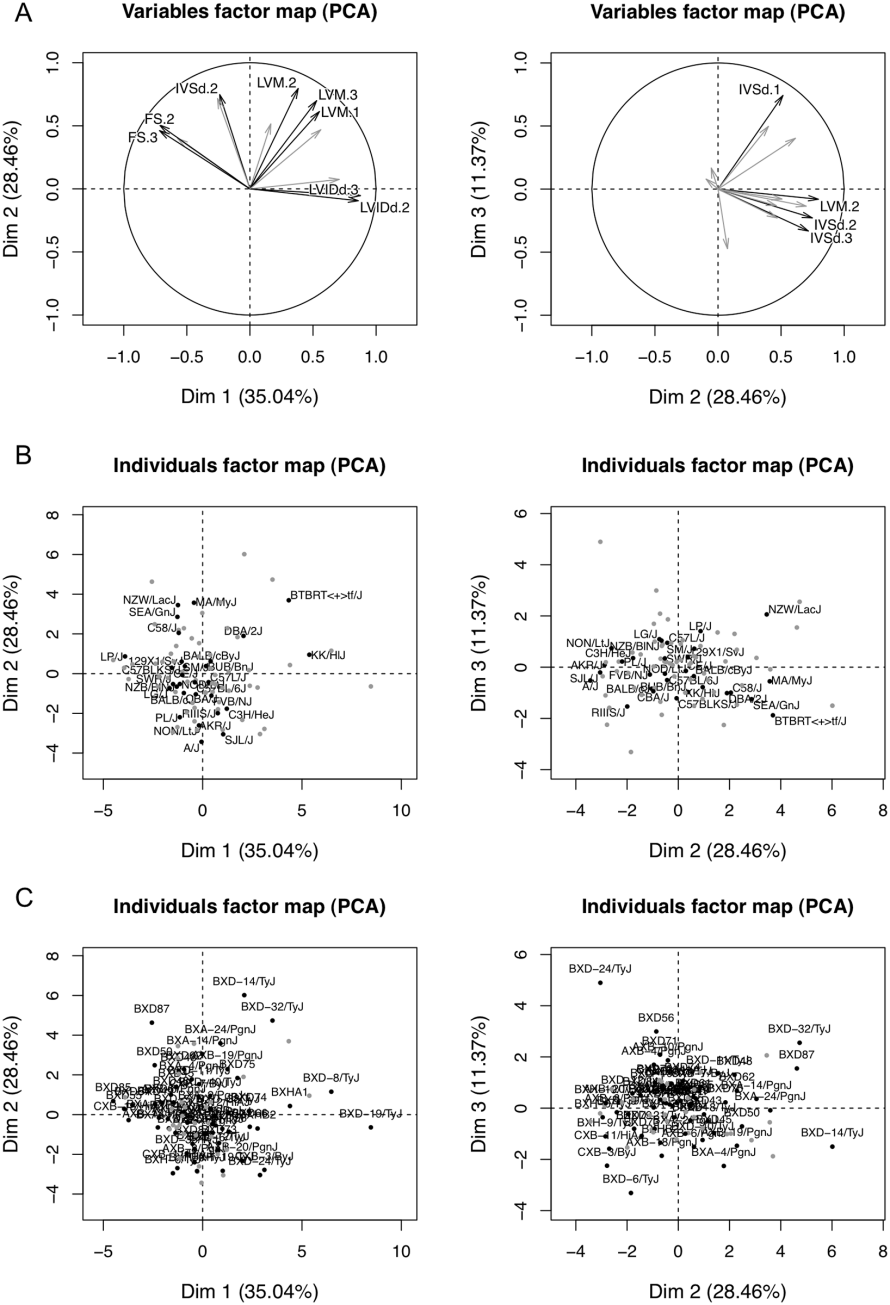
Principal component analysis of isoproterenol-induced changes from baseline. A. The variables factor map demonstrates the first three principal components that account for 74.8% of inter-strain variations. The first principal component corresponds roughly to LVIDd dilation and FS decrease. The second principal component corresponds to IVSd and LVM hypertrophy. The third principal component corresponds to early IVSd hypertrophy. The individual factor map projects the inbred mouse strains onto the first three principal components. Black dots highlight the classical (B) and recombinant (C) inbred strains and gray dots represent the remaining HMDP strains.

To describe each strain based on a composite of correlated traits, we used principal component (PC) analysis to transform ISO-induced changes into a set of linearly uncorrelated variables. The first three PCs account for 35%, 28% and 11% of the inter-strain variance observed, with the remaining PCs each accounting for < 10% of the variance observed. The variables factor map showed that the first three PCs captured phenotypes such as LVIDd dilation and FS decrease; LVM hypertrophy; and early IVSd increase (Fig 5A). The individuals factor maps highlighted KK/HlJ with decompensating features, such as LVIDd dilation and FS decrease, and NZW/LacJ with compensatory features, such as late IVSd and LVM increase and preserved FS (Fig 5B and 5C).

### Additive effects account for roughly half of the heritability estimates of cardiac structure and function across the HMDP

Heritability is the proportion of observed differences due to genetic variation in the population. Specifically, broad-sense heritability (H^2^) reflects all the genetic contributions to a population's phenotypic variance including additive, dominant, and epistatic effects, while narrow-sense heritability (h^2^) represents the proportion of phenotypic variance that is due to additive genetic effects alone. In inbred model organisms, where the environment is controlled and multiple animals of identical genetic background are available, between-strain and within-strain variation can be used to estimate H^2^, which reflects all genetic contribution to phenotypic variance. The majority of the traits we examined were highly heritable (H^2^ > 0.4) (S7 Fig). H^2^ estimates of LVM in the HMDP were between 61 and 81% (Table 1), which is similar to estimates for other common traits, such as body fat and insulin resistance, in the HMDP panel [18] and consistent with genetic factors making a significant contribution to cardiac structure and function. Narrow-sense heritability h^2^, the proportion of phenotypic variance due to additive genetic effects alone, incorporates genetic marker information and is the theoretical upper bound of what could be mapped in an association study. Narrow-sense heritability estimates h^2^ of LVM were from 39 to 57% (Table 1). Our results suggest that additive and non-additive effects, such as dominant and epistatic gene x gene interactions, each account for roughly half of the broad-sense heritability estimates in cardiac structural and functional traits in mice.

**Table 1:** Heritability estimates of cardiac structure and function under isoproterenol treatment.

r^2^	Control	Week 1	Week 2	Week 3
Broad-sense heritability estimates				
IVSd	0.61	0.59	0.49	0.43
LVIDd	0.76	0.68	0.67	0.68
LVM	0.81	0.70	0.61	0.74
FS	0.73	0.64	0.60	0.68
Narrow-sense heritability estimates				
IVSd	0.20 (0.12–0.31)	0.27 (0.15–0.44)	0.21 (0.09–0.41)	0.15 (0.06–0.35)
LVIDd	0.40 (0.29–0.51)	0.41 (0.27–0.57)	0.45 (0.31–0.60)	0.49 (0.34–0.64)
LVM	0.48 (0.38–0.59)	0.44 (0.31–0.58)	0.39 (0.26–0.55)	0.57 (0.43–0.70)
FS	0.30 (0.21–0.41)	0.31 (0.18–0.47)	0.34 (0.20–0.50)	0.38 (0.24–0.55)

Broad-sense heritability estimates are based on line repeatability. Narrow-sense heritability estimates are based on marker-based heritability estimates. Ninety-five percent confidence intervals are represented within the parentheses.

### ISO-induced gene expression changes across the HMDP are consistent with molecular, cellular and extracellular changes of cardiac remodeling

Human heart failure is characterized by chronic sympathetic activation to compensate for progressive cardiac function loss. Chronically, high levels of catecholamines may lead to calcium overload, initiating a cascade of alterations at the cellular level to result in cardiomyocyte death and progressive deterioration in cardiac structure and function. To protect itself from harmful effects of chronic adrenergic stimulation, a protective mechanism, called desensitization, reduced membrane availability of the beta-adrenergic receptors, leading to diminished downstream signaling. ISO is a non-selective β-adrenergic agonist that, when administered chronically in mice, mimics the state of chronic sympathetic activation in human heart failure [41].

Consistent with molecular changes of cardiac remodeling in humans, ISO resulted in gene expression changes downstream of β-adrenergic receptor signaling across the HMDP mice. β1-adrenoceptor expression decreased by 17% (*Adrb1*, adjusted p-value = 2.56 x 10^−5^) and the inhibitory G-protein G_i_ increased by 16% (*Gnai2*, adjusted p-value = 4.47 x 10^−7^), reminiscent of diminished β-adrenergic responsiveness secondary to chronic β-adrenergic overdrive in cardiomyopathic hearts (S2A Table). In addition, ISO downregulated *Myh7* expression by 34% and *Myh6* expression by 15% (adjusted p-value = 6.93 x 10–7 and 6.77 x 10–4, respectively), possibly reflecting down-regulation of cardiac structural proteins or increased fibrotic tissue. Finally, one of the most significantly up-regulated genes *Lgals3* was increased by 3.5-fold (adjusted p-value = 1.62E-22). *Lgals3*, or galectin-3, is an emerging clinical marker of cardiac fibrosis, adverse LV remodeling, and mortality in HF patients [42].

Gene ontology analysis of the up- and down-regulated probes was performed to examine the global changes in gene expression due to ISO. The up-regulated probes were most enriched for secreted signal glycoprotein, proteinaceous extracellular matrix (ECM), angiogenesis, polysaccharide binding, actin cytoskeleton, vacuole, response to wounding, chemokine signaling pathway, and epidermal growth factor (EGF)-like calcium-binding, prenylation and growth factor binding (S2B Table). The down-regulated probes were most enriched in mitochondrial matrix, mitochondrial inner membrane, and flavoprotein (S2C Table). Gene ontology analysis of the top 1000 correlated transcripts for each phenotype-expression pair was performed to identify processes specific for each phenotype. Under the baseline condition, transcripts that correlated with LVIDd were modestly enriched for actin-binding, cell junction and synapse. At week 3 of ISO, transcripts that correlated with LVID and LVM were highly enriched for proteinaceous ECM, secreted signal glycoprotein, actin cytoskeleton, collagen, ECM receptor interaction, EGF, polysaccharide binding, and mitochondrial membrane (S2D Table). These findings provided strong evidence that ISO induced cellular and ECM changes of cardiac remodeling found in HF.

### Association mapping of cardiac remodeling and gene expression identified candidate genes for 17 cQTL associated with ISO-induced cardiac remodeling traits

Association mapping at each ISO-treated time point was performed to define cQTLs for BBW- and HR-adjusted echocardiographic changes in measures from baseline. In addition, association mapping was performed for each gene transcripts under baseline and ISO-treated conditions, to define local expression quantitative trait loci (cis-eQTL) near each cQTL. Because genetic information flows from DNA to transcript to clinical phenotypes, a transcript with an eQTL that coincides with cQTL of a trait of interest may be hypothesized to be causal for the trait. Using a custom pipeline that overlays cQTL, cis-eQTL, expression-phenotype correlation, and structural variation, candidate genes for each cQTL were prioritized. The significantly associated loci and candidate genes for echocardiographic measures are provided in Table 2. All significantly associated loci as well as supporting evidence described above are provided in S6 Table. All SNPs with association p-value < 0.001 are provided in S7 Table.

**Table 2:** Significant association loci.

Chr	Start	End	Range	Trait	rsID	P-value	MAF	ES	ω^2^	Analyses	Candidate genes
1	187.4	187.6	0.2	LVIDd	rs32292745	2.97E-06	0.08	0.21	1%	1dLVIDd	*Lyplal1*
3	82.1	82.6	0.5	FS	rs32712632	9.06E-07	0.3	6.1	14%	3FS	**Tdo2**, **Accn5**, **Gucy1a3**
3	94.3	95.7	1.4	LVIDd	rs33064660	1.30E-06	0.22	0.29	7%	3LVIDd	*Rorc*, **Oaz3**, **Mrpl9**, Snx27*, **Selenbp1**, **Psmd4**, **Tmod4**, **Gm128**, **Bnipl**, **Anxa9**, **Lass2**, **Ctsk**, Golph3l, **Ecm1**, **Prpf3**, **Mrps21**, **Gm129**, **BC028528**, **Car14**
4	54.2	55	0.8	LVM	rs27794497	9.30E-07	0.36	18.2	11%	3LVM,3dLVM,1LVM,1dLVM	***Klf4***
4	58.2	58.3	0.1	LVIDd	rs27851114	2.78E-06	0.06	0.2	6%	1dLVIDd	**Txndc8**, Svep1, Musk
4	62.4	62.7	0.3	LVM	rs28295600	3.77E-06	0.39	18.2	9%	3LVM	*Rgs3*, Zfp618, **Orm3**, **Whrn**
4	63.7	64	0.3	LVM	rs3656076	1.74E-07	0.44	20.8	8%	3LVM,3dLVM	Tnc
4	93.1	95.2	2.1	IVSd	rs28128253	4.65E-09	0.36	0.10	15%	1IVSd	**Jun**
7	51.5	52.4	0.9	LVM	rs40560913	1.44E-09	0.4	18.8	10%	3LVM,3dLVM,2LVIDd,iLV,dLV	Myh14, Izumo2*
9	66.9	69.6	2.7	IVSd	rs49424819	7.35E-07	0.35	0.09	1%	1dIVSd	M5C1000I18Rik, ***Vps13c***
9	74.9	75.8	0.9	FS	rs33896682	3.41E-06	0.40	3.22	4%	2dFS	Arpp19*, Bmp5
9	75.8	80.2	4.4	IVSd	rs13480288	1.95E-09	0.38	0.10	12%	1dIVSd	**Tinag**, Lrrc1*, **Omt2b**, **Impg1**
9	84	84.6	0.6	IVSd	rs36266287	6.89E-07	0.38	0.09	6%	1dIVSd	Bckdhb, Fam46a
11	36.8	36.9	0.1	LVIDd	rs6333970	2.83E-06	0.31	0.25	8%	1LVIDd	Tenm2
12	57.8	57.9	0.1	LVIDd	rs47048438	3.41E-06	0.07	0.20	1%	1dLVIDd	**Nkx2-9**, Slc25a21*
15	40	40.1	0.1	FS	rs48791248	2.71E-07	0.10	6.79	14%	1FS	Dpys, **Lrp12**, **Zfpm2**
18	47	49.2	2.2	FS	rs51860788	1.04E-06	0.41	6.62	6%	2FS	**Trim36**

Start and end positions as well as range are in the unit of Mb. Peak p-value and SNP rsID and are listed. If multiple SNPs have the same p-value, only one representative rsID is listed and indicated by an underline. MAF denotes the minor allele frequency. ES denotes the effect size per allele. The units for the effect sizes are mm for IVSd and LVIDd, mg for LVM, and % for FS. Variance explained is denoted by ω^2^. Analyses are the analyses in which the SNP exceeded the genome-wide significant threshold. If multiple analyses yielded significant p-value at a given SNP, the underlined analyses has the most statistically significant p-value. Candidate genes are denoted by structural variation (underlined), cis-eQTL (italics) and correlation with trait were present (bold). *Golph3l* and *Bmp5* have a stop_gained variant. *M5C1000I18Rik* and *Dpys* have a splice acceptor variant. *Dpys* has 2 missense variants.

* denotes that one of the lead SNPs resided in the gene.

A total of 17 genome-wide significant cQTL were identified, including 4 for IVSd, 5 for LVIDd, 4 for LVM and 4 for FS, many of which were less than 1 Mb in size (Table 2). As expected, some of the correlated measures mapped to the same loci across ISO-treated time points or traits (S8 Fig). We compared our loci with available cardiovascular disease GWAS loci in human (S8 Table). One of the loci for week 1 IVSd hypertrophy near *Fam46a* on chromosome 9 has previously been shown in a multi-ethnic GWAS meta-analysis to be associated with systolic and diastolic blood pressure (p-value = 6.2 x 10^−5^ and 5.5 x 10^−7^, respectively) [43]. One of the loci for week 1 FS near *Lrp12* and *Zfpm2* on chromosome 15 has previously been shown in a small human GWAS to be associated with sudden cardiac arrest due to ventricular tachycardia and ventricular fibrillation in patients with coronary artery disease (p-value = 1 x 10^−6^) [44].

### *Klf4* is a causal gene for ISO-induced LVM hypertrophy

**Fig 6:**
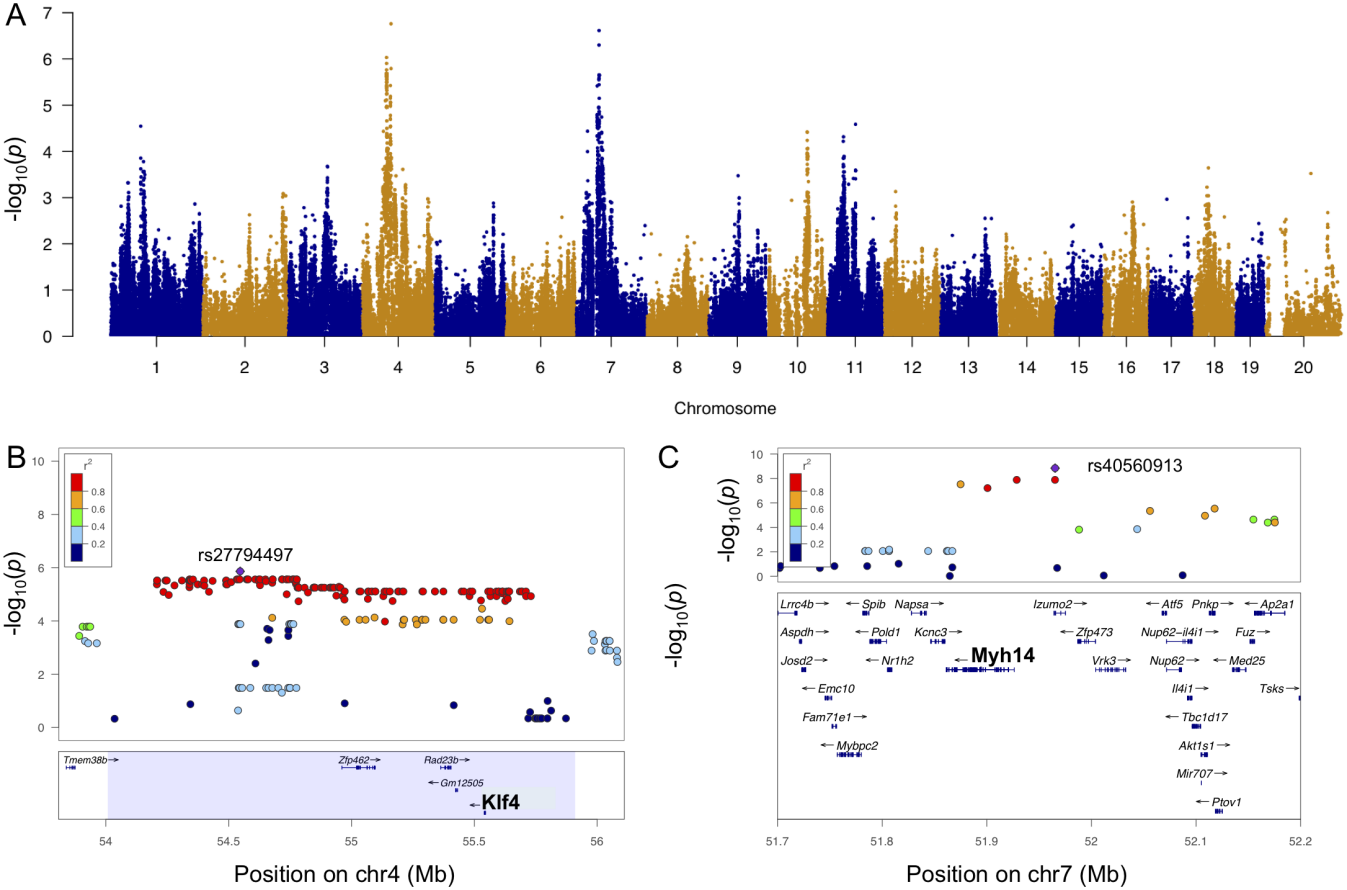
Genome-wide association for week 3 LVM. A. Manhattan plot for week 3 LVM. B. Regional plot for week 3 LVM around peak SNP rs27794497 (purple). C. Regional plot for week 3 LVM hypertrophy around SNP rs40560913 (purple). Pairwise r2 between the peak SNP and the surrounding SNPs are denoted by color scale.

A locus on chromosome 4 was significantly associated with LVM at week 3 (Fig 6A). LVM hypertrophy at weeks 1 and 2 and LV weight hypertrophy were also associated with this locus at lesser significance levels, while baseline LVM did not map to this locus (S8A–S8C Fig). Mouse strains with the GG and the TT genotypes at the lead SNP rs2779449 (p-value = 9.3 x 10^−7^) conferred a median week 3 LVM of 117 mg and 135 mg, respectively. SNP rs27794497, located in an intergenic region between *Tmem38b* and *Zfp462*, and surrounding SNPs in LD (r^2^ > 0.8) spanned a region containing 4 genes (Fig 6B). Among these genes, *Klf4* alone had a significant cis-eQTL at SNP rs27794497 (p-value = 5.9 x 10^−5^) and was significantly correlated with LVM hypertrophy at week 3 (bicor = -0.3, p-value = 0.0088; S8D and S9 Figs). Our results suggest that *Klf4* is a negative modulator of ISO-induced LVM. During the course of this study, Yoshida *et al*. demonstrated that, when subjected to ISO, cardiomyocyte-specific *Klf4* knock-out mice demonstrated enhanced cardiac hypertrophy, augmented cellular enlargement, exaggerated expression of the transcriptional regulator myocardin and fetal cardiac genes [45].

### *Myh14* is a causal gene for ISO-induced LVM hypertrophy

**Fig 7:**
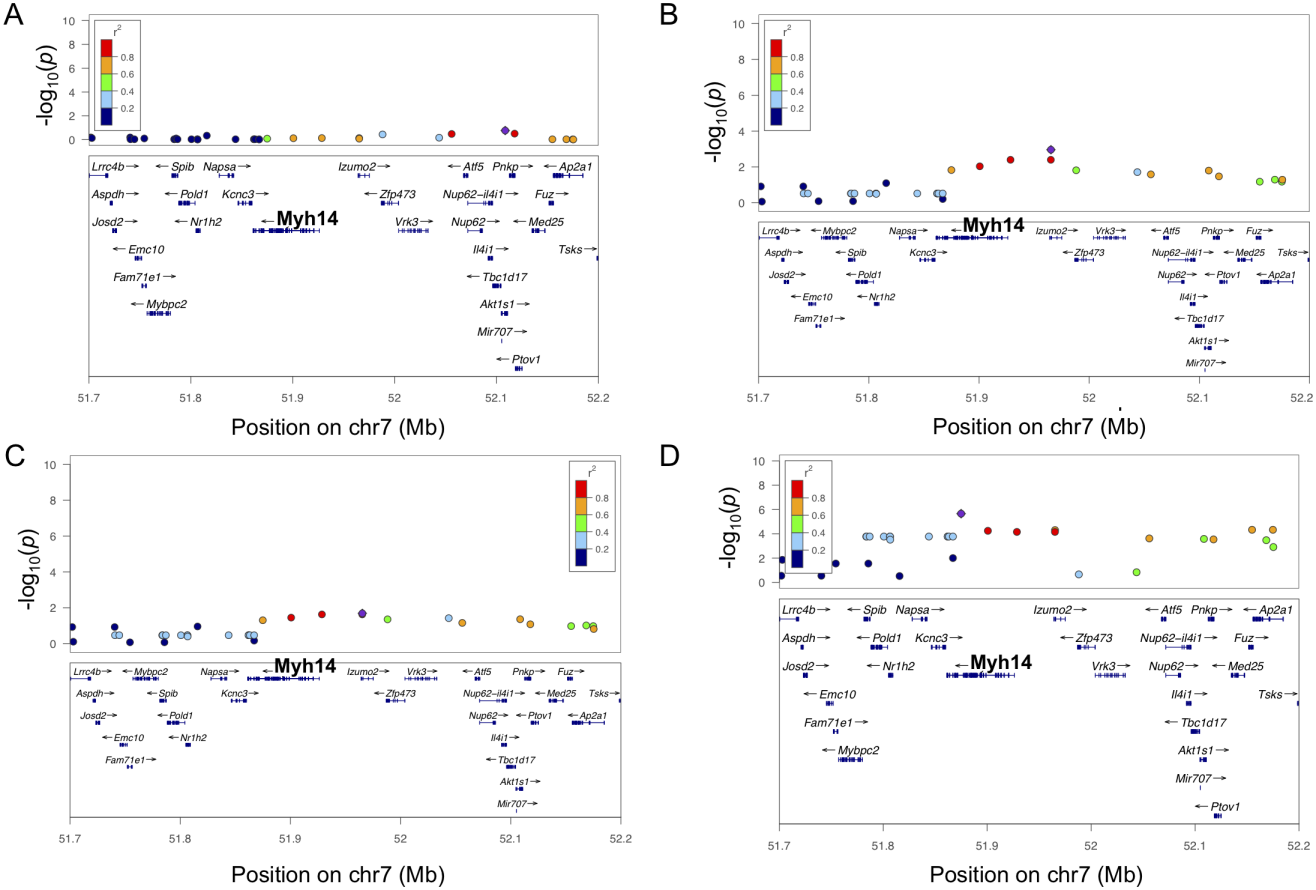
Regional plots of LV hypertrophy at chromosome 7 across time points. A. Regional plot for LVM at baseline. B. Regional plots for ISO-induced LVM hypertrophy at week 1. C. Regional plots for ISO-induced LVM hypertrophy at week 2. D. Regional plot for ISO-induced LV weight hypertrophy at week 3.

In addition, a locus on chromosome 7 was significantly associated with LVM hypertrophy at weeks 3 (Fig 6A). This locus was also associated with LVM hypertrophy at week 1 and LV weight hypertrophy at lesser significance levels, while baseline LVM did not map to this locus (Fig 7). Mouse strains with the AA and the GG genotypes at the lead SNP rs40560913 (p-value = 1.44 x 10^−9^) conferred a median LVM increase of 43 mg and 28 mg, respectively. SNP rs40560913, located in the intron of *Izumo2*, and surrounding SNPs in LD (r^2^ > 0.8) spanned only one other gene *Myh14* (Fig 6C).

Neither *Izumo2*, a sperm-specific gene, nor *Myh14* was represented on the expression array used in our study. By qPCR of available matching control and ISO RNA samples in 26 mouse strains, we found *Myh14* expression to be increased by 46% with ISO treatment (p-value = 0.002). The available qPCR results was not adequate to map for eQTL. As *Myh14* was represented on microarray platforms used in prior HMDP studies, we queried our cis-eQTL database and found that *Myh14* has cis-eQTL signals across multiple tissues at the same locus and its expression in adipose and liver was negatively correlated with body mass normalized heart weight at week 8 in the HMDP obesity study (S9 and S10 Tables) [18]. Based on these findings, we hypothesized that *Myh14* was the causal gene for this locus.

**Fig 8:**
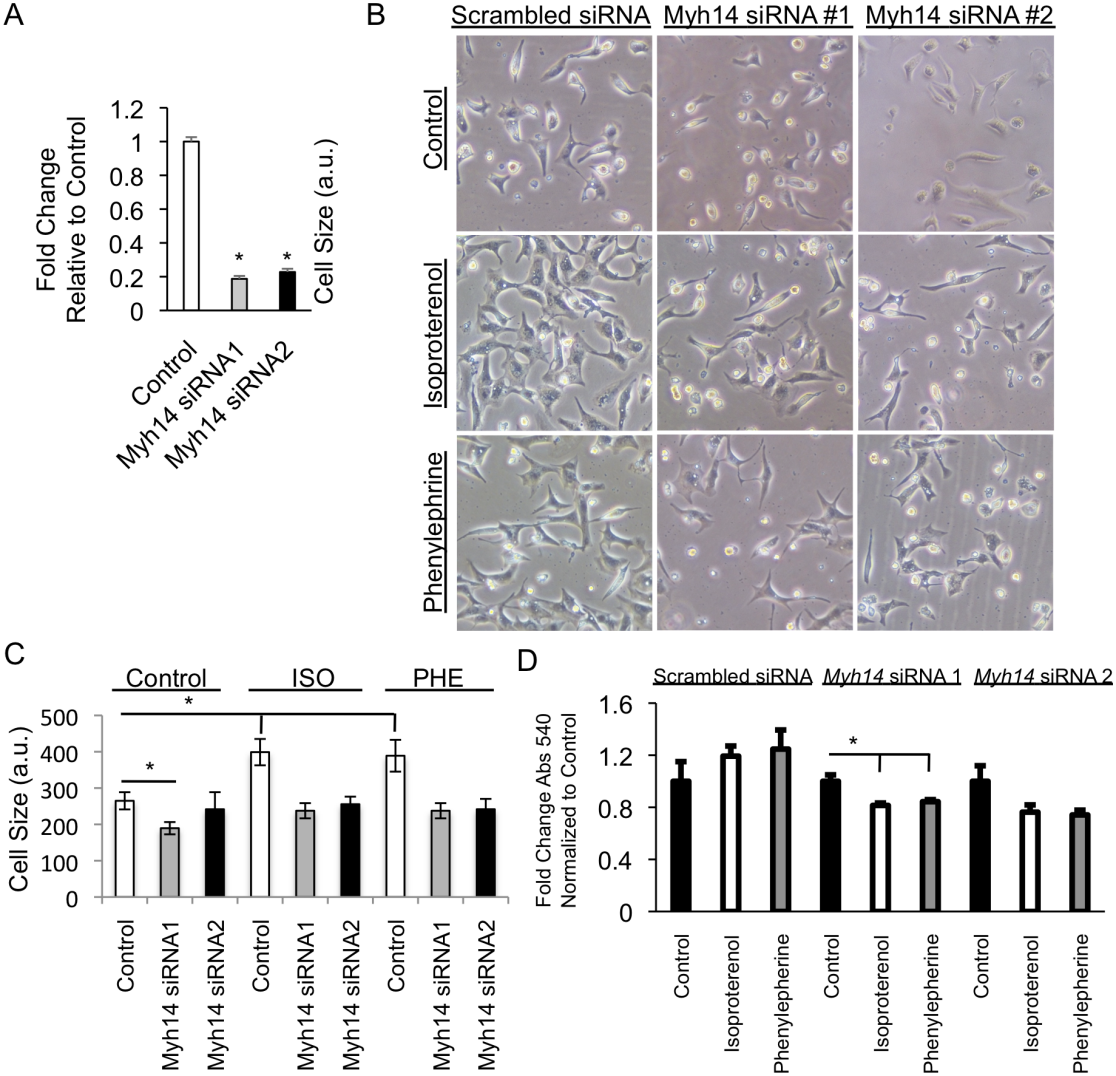
*Myh14* knock-down in stressed neonatal rat ventricular myocytes (NRVMs) causes abrogation of hypertrophic response and decreased cell viability. Knock-down of *Myh14* in NRVMs using siRNA (A), followed by ISO and phenylephrine (PHE) treatments, showed the *Myh14* knock-down treated NRVMs displayed poor attachment to tissue culture plates (B), did not undergo characteristic hypertrophy (C) and resulted in decreased cell viability based on MTT assay (D). Error bars are SEM, n = 3. * denotes p < 0.05.

MYH14 is a non-muscle myosin involved in mechanotransduction—knockout of the gene/protein has no basal phenotype [2] and the role of MYH14 has never been comprehensively determined in the heart. To validate *Myh14* as a causal gene for LVM hypertrophy, we performed siRNA knock-down of *Myh14* in neonatal rat ventricular myocytes (NRVM) followed by phenylephrine and ISO stimulation *in vitro*. *Myh14* knock-down demonstrated > 70% efficiency (Fig 8A). *Myh14* siRNA treated cells displayed poor attachment to the tissue culture plates (Fig 8B). In addition, quantitative measurements of cell size showed that *Myh14* siRNA treated NRVMs failed to undergo characteristic cellular hypertrophy under phenylephrine (PE) and ISO stimulation (Fig 8C) and resulted in decreased cell viability (Fig 8D).

**Fig 9:**
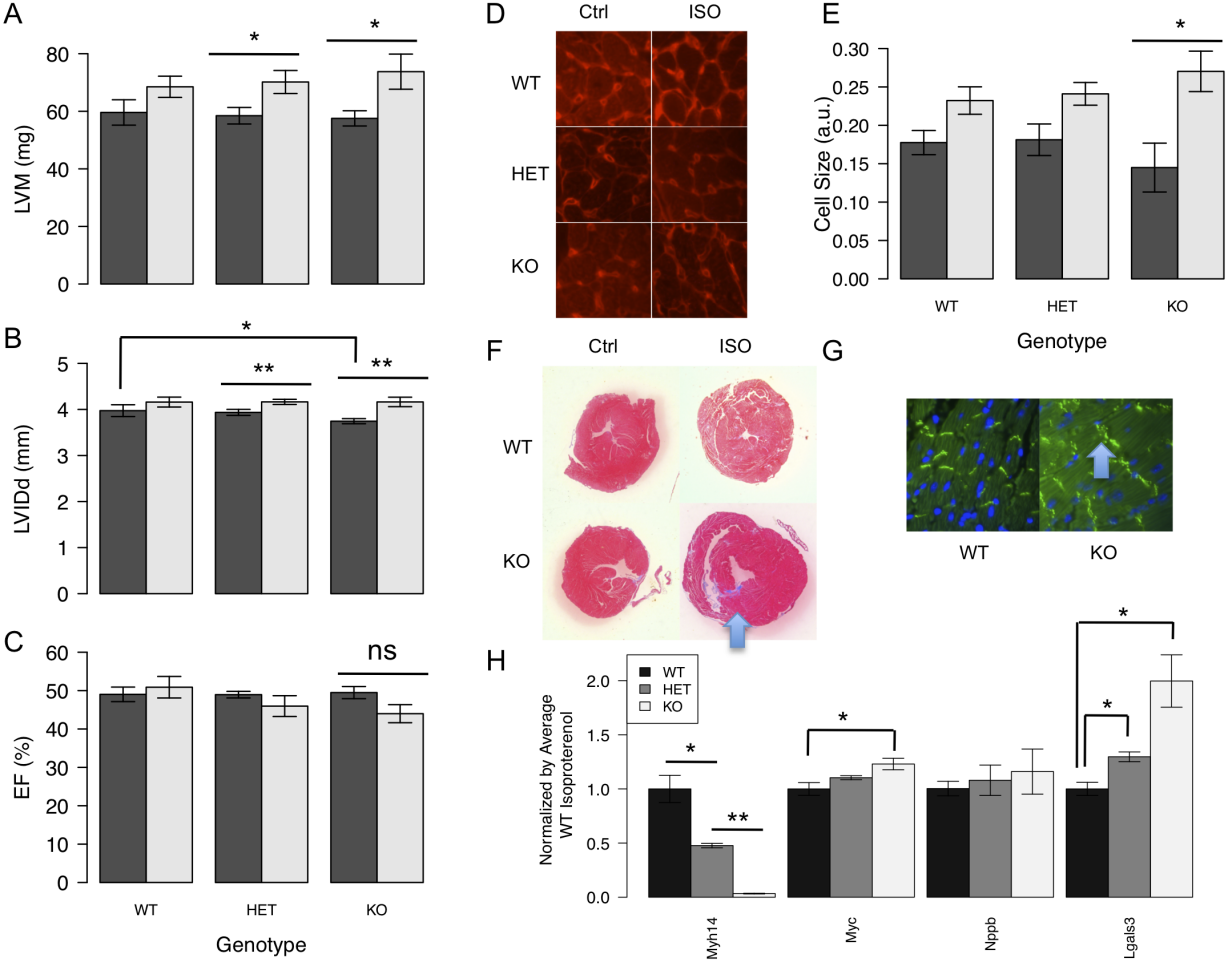
*Myh14* deficiency under ISO results in LVM hypertrophy, LVIDd dilation, increased cardiomyocyte size, fibrosis, intercalated disc disarray, and hypertrophic signals. Female mice of wild-type (WT), heterozygous (HET) and knockout (KO) *Myh14* genotypes were treated with ISO at 10–12 weeks of age (n = 6, 9, and 7, respectively). *Myh14* deficiency conferred an increase in ISO-induced LVM hypertrophy (A) and LVID dilation (B). There was a trend towards decreased ISO-induced EF (C) due to *Myh14* deficiency. Cardiomyocyte cross-sectional area, as measured by wheat germ agglutinin (WGA) staining (D), showed that cardiomyocytes from ISO-treated KO mice were more significantly hypertrophied compared to HET and WT mice (E). Dark gray bars (baseline in A and B; average of baseline and week 1 in C; control in E). Light gray bars (average of weeks 1–3 measures in A and B; average of weeks 2–3 measures in C; ISO-treated in E). *Myh14* deficiency conferred an increase in ISO-induced fibrosis (blue arrow) by Masson’s trichrome staining (F) and an increase in intercalated disc disarray (blue arrow) by β-catenin staining (G). Cardiac tissue gene expression of hypertrophic and fibrosis markers were examined by RT-PCR at the end of a 3-week ISO infusion (H). *Myc*, *Nppb*, and *Lgals3* were increased with *Myh14* deficiency. Error bars are SEM, n = 3. * represents t-test p-value < 0.05. ** represents t-test p-value < 0.005.

To further examine the role of *Myh14* in cardiac remodeling *in vivo*, we obtained *Myh14*^-/-^ mice from the Adelstein lab [40]. Wild-type, heterozygous (*Myh14*^+/-^) and homozygous (*Myh14*^-/-^) littermates were not phenotypically different and no disproportionate spontaneous sudden death was observed in *Myh14* deficient mice (oldest *Myh14*^-/-^ mouse 12 months to date). qPCR analysis showed a complete loss of *Myh14* expression in *Myh14*^-/-^ hearts (Fig 9H). ISO-induced LVM and LVIDd hypertrophy were significantly increased in *Myh14*^-/-^ and *Myh14*^+/-^ versus wild-type controls (Fig 9A and 9B). There were no significant differences in baseline EF among genotypes but ISO-treated *Myh14*^-/-^ mice demonstrated a trend towards EF reduction compared to *Myh14*^+/-^ and WT mice (Fig 9C). Cardiomyocyte cross-sectional area, as measured by wheat germ agglutinin (WGA) staining, showed that cardiomyocytes from ISO-treated *Myh14*^-/-^ mice were more significantly hypertrophied compared to *Myh14*^+/-^ and WT mice (Fig 9D and 9E). Histological sections of *Myh14*^-/-^ hearts revealed no abnormal phenotype at baseline but increased myocardial fibrosis and intercalated disc disarray after ISO treatment (Fig 9F and 9G). RT-PCR demonstrated the up-regulation of immediate early gene *Myc*, fetal gene *Nppb*, and fibrosis gene *Lgals3* in ISO-treated *Myh14* deficient hearts compared to controls (Fig 9H).

### The HMDP resource

The study data, including gene-gene and gene-trait correlations as well as clinical trait and transcript mapping, can be accessed via a user-friendly web-based interface at http://systems.genetics.ucla.edu/.

## Discussion

Human HF GWAS have identified a handful of genetic determinants that explain a small proportion of the genetic variance, in spite of involving tens of thousands of individuals [46]. Given the breadth of etiological and clinico-pathological heterogeneity, even larger cohorts may not be big enough to discriminate signals from noise. We carried out an alternative approach in the mouse, which has several advantages over human. First, we used multiple mice per strain and were able to fully control the age, environment, severity and timing of cardiac injury, to accurately assess cardiac remodeling with minimal confounding. As a result, our heritability estimates of LVM were significantly higher than that in human of 24–32% in the Framingham Heart Study [47] and 36–47% in an Australian twin study [48]. The relatively high heritability provided sufficient power to uncover novel loci not previously identified in human. Second, the HMDP strains have either been sequenced or densely genotyped, eliminating genotyping costs and errors. Third, access to control and ISO LV transcriptome as well as sequence variation in 17 of the classical inbred strains available from the Wellcome Trust Mouse Genomes Project (MGP) allowed us to examine cis-eQTL, transcript-to-trait correlations, and functional variants to prioritize candidate genes and generate hypotheses for functional validation without prior knowledge in an unbiased manner, thus overcoming some of the challenges of working with relatively long LD in mice. Given the paucity of association signals in humans, we consider the association signals we observed in mice to be complementary to existing human studies. While the individual genetic variants observed in mice may not be identical to the variants observed in human populations, candidate genes from this study help identify genes and/or pathways participating in modifying mechanisms of heart failure pathology.

We recognize that using only one gender is a limitation of our study that prevents us to readily extrapolate our findings to male mice. During the pilot phase of the study, we observed a slightly wider phenotypic separation among the 4 core classical inbred strains (A/J, C57BL/6J, C3H/HeJ, and DBA/2J) in females than in males, which is important for our association mapping study design. Under the constraints of cost for animal procurement and maintenance, we chose to study females only. We believe that at least some of the findings observed in females will likely recapitulate in males, since gender differences in mortality in humans appear to be predominantly due to factors, such as age, co-morbidities and treatment approaches [49], rather than gender itself. Moreover, higher rates of mortality and cardiogenic shock have been observed in women compared with men after acute myocardial infarction [49], yet females are under represented in both human and mouse studies.

Our study established an echocardiographic reference range for 100+ inbred mouse strains. To minimize the variability to handling among different mouse strains, inhaled isoflurane, one of the most commonly used agent for prolonged anesthesia in mice, was administered during echocardiography. Based on recent studies on sedated mice, FS of 33% and 32% in 129X1/SvJ male and C57BL/6J female mice were similar to our strain averages of 33% and 34%, respectively [50,51].

Inhaled anesthetics, including isoflurane, are known cardiac depressants, especially upon deep sedation. In a study involving C57BL/6 male mice, mean arterial pressure (MAP), HR, and body temperature at an isoflurane concentrations of 1.5% (volume-to-volume, v/v) remained comparable to the conscious state for up to 90 minutes post-induction [52]. In another study using C57BL/6 male mice, where HR was fixed at different dosages/depths of isoflurane [53], echocardiographic measurements were found to be highly reproducible at a high HR (475–525 bpm) but were correlated with HR under a low HR (350–400 bpm). Although no comparable prior data exist for the vast majority of the mouse strains in our study, we empirically observed that different mouse strains exhibited variable sensitivity to anesthesia and that HR correlated to a number of echocardiographic measures representing cardiac inotropy and chamber size (S6B Fig). Therefore, we adjusted for HR as a covariate and a surrogate for isoflurane sensitivity in our analysis. In spite of concerns regarding HR being a confounding factor and surrogate marker for variable isoflurane sensitivity, correlations among echocardiographic measures remained roughly unchanged, before and after adjusting for HR, consistent with relatively minor cardiac depressive effects of isoflurane.

In spite of ongoing inotropic stimulation by ISO throughout the 3-week treatment time course, some strains displayed the same or decreased FS at later ISO time points compared to baseline, consistent with a decrease in contractile reserve—an observation in early HF stages of decreased cardiac function augmentation to direct inotropic stimulation—marked by reduced beta-adrenergic receptors along with altered sarcoplasmic reticulum Ca2+-adenosine triphosphatase 2a (SERCA2a) and phospholamban [54]. Our narrow-sense heritability estimates were roughly half of broad-sense heritability estimates, suggesting that both additive and dominant/epistatic genetic effects play important roles in the genetic variance of HF.

Our association analyses provided direct evidence that common genetic variation control ISO-induced cardiac remodeling. The following considerations are relevant to the interpretation of the results. Equivalent dosages of ISO were given to each strain to control the degree of cardiac insult. Although we could not control for ISO metabolism, we believe its contribution to the results was minor. Linkage analysis using only the BXD RI strains resulted in a significant reduction in resolution (S10 Fig). On the other hand, association analysis using only the classical inbred strains suffered a significant loss of power (S11 Fig). Our study, which combines the classical inbred and RI strains to achieve high genetic resolution and adequate statistical power, represents a substantial improvement over traditional linkage analysis. In addition, panel-specific SNP selection based on minor allele frequency and missing genotype cutoffs could indirectly affect association results. For example, SNPs that did not meet the minor allele frequency and missing genotype cutoffs in the entire HMDP panel resulted in the loss of association signals around the 40 Mb region on chromosome 7 (S11C Fig). Moreover, genetic variation that is well represented among the BXD panel could contribute more substantially to the association results, due to the fact that the BXD panel (44 strains) was highly represented among the entire HMDP panel (104 strains) (S10 Fig). In the setting of a relatively narrow phenotypic spectrum, adjustments for uncorrelated covariates can deflate the association results. For example, baseline FS, which was not correlated to BBW and has a similar narrow-sense heritability estimate as ISO-treated FS, had two associated loci prior to but none after BBW adjustment. Finally, the significantly associated loci explained about 23–38% of the total variance observed, which is significantly higher than in human HF GWAS.

In our previously published manuscript, we identified a number of heart weight and fibrosis loci but none for ISO-induced LV weight hypertrophy [21]. A number of factors may have hampered the detection of association signals for LV weight. For example, LV weight hypertrophy for each strain was estimated based on the difference of the strain averages from control and ISO-treated cohorts. Given the small numbers of individuals in each cohort, variation in LV weight due to variation in body size and cardiac chamber dissection may have introduced errors in ISO-induced LV weight hypertrophy estimates per strain. In contrast, LVM was measured non-invasively, which allowed for repeated measurements in the same animal. The baseline LVM estimate served as an internal control measure for body size in each mouse treated with ISO. This approach represented an important advantage over our previous analysis, which allowed us to estimate ISO-induced LVM hypertrophy more accurately and improve the power of association analysis. Of note, the LVM measurements demonstrated a positive bias in the upper ranges as a result of its derivation, which contains a constant multiplier:

$$\text{LV mass }(\text{Penn})\;=\;1.04\;([\text{LVIDd }+\text{ PWd }+\text{ IVSd}{]}^{3}\;-\;[\text{LVIDd}{]}^{3})\;-\;13,6\text{ g},$$

where LVIDd represents left ventricular internal dimension at end diastole, PWd represents posterior wall thickness at end diastole, and IVSd represents interventricular septal wall thickness at end diastole.

In a mapping study, the spread rather than the absolute values of the data drives the association analysis. Therefore, in spite of the positive bias in the upper ranges, LVM and LV weights yielded similar association results (Figs 6C and 7D), with LV weight having a reduced statistical significance as compared to LVM.

**Fig 10:**
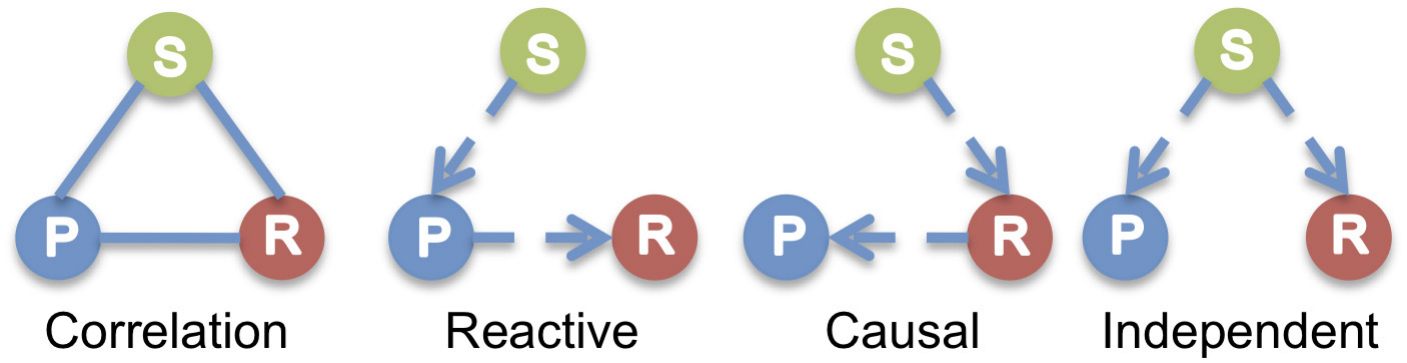
Relationships between variation and correlated traits. There are three possible causal relationships when there is correlation among a SNP (single nucleotide polymorphism) S, a transcript (RNA) R, and a physiological or pathologic trait (phenotype) P. In the causal model, the SNP variation affects its transcript levels leading to the resulting phenotype. In the reactive model, the SNP acts on the phenotypes, which in turn affects transcript. In the independent model, the SNP variation acts on both the phenotype and transcript independently.

Our association analysis not only highlighted a number of genomic regions and genes for HF susceptibility in mice, it also provides independent supportive evidence for syntenic regions in humans and is complementary to existing and future human HF genome-wide association and family-based linkage studies. For example, a candidate causal genomic region or gene for HF or inherited cardiomyopathy in humans not reaching statistical significance may be recovered based on additional supportive evidence from mice. Next, based on the central dogma that the flow of genomic information starts from the DNA, to the RNA then to the phenotype of interest, the cardiac transcriptome data allow us to generate hypotheses to test causal inference. For instance, when the transcript level of a gene in an association locus is associated with genetic variation and correlates with phenotype, we may hypothesize that the gene could be causally related to the phenotype, thereby facilitating prioritization of candidate genes in an association locus (Fig 10). Our data could be used to explore the roles of individual candidate genes in existing human GWAS loci. In addition, we have provided a rich data resource for the HF research community to interrogate a given gene’s relationship with other genes in the cardiac transcriptome. For example, a HF community researcher studying a novel HF gene X may be interested in its upstream regulatory and downstream regulated genes. In addition to identifying other genes in the cardiac transcriptome that correlated with gene X, a search for gene X’s trans-eQTL, a distant locus controlling the expression of gene X, may reveal a number of putative upstream regulators of gene X for hypothesis driven experiments and testing. On the other hand, if gene X was regulated at the local level, identification of additional cardiac transcripts regulated by the same locus could reveal gene X’s putative downstream targets. Finally, the differentially expressed cardiac transcripts that correlated with cardiac remodeling phenotypes represent a set of genes that may play important roles in HF pathogenesis and compensatory changes and may be enriched for HF biomarkers. Our transcriptome data could complement existing human transcriptome data, a valuable set of transcripts that represent the true spectrum of human HF but whose RNA sample quality may be more variable, to identify additional players of HF pathogenesis, compensatory changes and biomarkers.

Our study implicated *Klf4* and *Myh14* to be negative regulators of cardiac hypertrophy under ISO stress. Our results showed that the degree of LVM hypertrophy was inversely correlated with the expression of *Klf4* (S9 Fig). Since the expression of *Klf4* expression was genetically determined by the cis-eQTL, our findings suggest that *Klf4* expression may be causally related to the degree of LVM hypertrophy. In other words, the higher the Klf4 expression, the lesser the degree of LVM hypertrophy. We note, however, that our data does not provide proof of a causal relationship, since a significant fraction of *Klf4* expression occurs in trans, but is consistent with findings from previous literature showing that KLF4 is a negative modulator of ISO-induced LVM. *Klf4*, or Krüppel-like factor 4, a zinc-finger transcriptional regulator best known as one of the four Yamanaka factors that are sufficient to reprogram differentiated cells into embryonic-like induced-pluripotent stem cells (iPSCs) [55], has recently emerged as an important modulator of cardiomyocyte hypertrophy. Cardiomyocyte-specific deletion of *Klf4* in mice resulted in a slightly increased heart weight and increased ANF levels compared to controls. When subjected to pressure overload, these mice developed increased pathologic cardiac hypertrophy, fibrosis, apoptosis and mortality, compared to control banded mice [56]. *In vitro* studies confirmed that KLF4 binds to the ANF and GATA4 promoters and acts as a repressor of cardiomyocyte hypertrophy [57].

*Myh14* was the only gene expressed in the heart that was located at the chromosome 7 locus affecting cardiac hypertrophy in our study. *Myh14*, myosin heavy polypeptide 14, encodes the heavy chain of the molecular motor nonmuscle myosin II-C (NMIIC), which is a member of the nonmuscle myosin II motor protein family that plays an integral role in mechanotransduction, converging external and cell-generated forces by interacting with cytoskeletal actin. When bred in a background of non-muscle myosin II-B (NMIIB) hypomorphic mice expressing only 12% of wild-type amounts of NMIIB protein, *Myh14*^-/-^ mice developed marked cardiac myocyte hypertrophy, interstitial fibrosis and diffuse N-cadherin and β-catenin patterns at the intercalated discs, where NMIIB and NMIIC colocalized [40]. Collectively, these prior studies implicated *Klf4* and *Myh14* as negative regulators of stress-induced cardiac hypertrophy *in vivo*, which is also supported by our results.

In our *in vivo* study, we showed that *Myh14* deficient mice exhibited increased ISO-induced LVID dilation, LVM hypertrophy, cardiac fibrosis, and hypertrophic markers *Myc* and *Nppb*. On the other hand, *In vitro* siRNA knockdown of *Myh14* in NRVMs failed to elicit a hypertrophic response when stimulated with ISO but rather displayed poor attachment to the tissue culture plates and poor survival. As a non-muscle myosin, *Myh14* is ubiquitously expressed and could act through non-cardiomyocytes to modulate cardiomyocyte growth and hypertrophy. We focused on *Myh14* inside cardiomyocytes due to a previous study demonstrating the location of *Myh14* in the intercalated disc and its implicated role at the cell-cell junction [40]. Since NRVMs in tissue culture do not fully recapitulate the three-dimensional structure of the heart, the consequence of *Myh14* deficiency may be quite different *in vitro* versus *in vivo*. These seemingly discrepant results raise important questions regarding the mechanisms through which *Myh14* deficiency leads to cardiomyocyte death in the absence of mechanotransduction stress *in vitro* and cardiac hypertrophy on an organ level *in vivo*.

To further explore mechanisms mediating *Myh14* deficiency in LVM hypertrophy, we queried the existing HMDP cardiac transcriptome database available via http://systems.genetics.ucla.edu/ [58]. We found that the cis-eQTL locus that controls *Myh14* expression also controls *Foxo1* in trans (p-value = 1.20x 10^−5^) and that the expression of *Foxo1* was significantly positively correlated with *Myh14* (bicor = 0.74, p-value = 6.7 x 10^−18^), which implicated *Foxo1*, a forkhead family transcription factor, as a potential mediator of *Myh14*. In addition, *Myh14* expression was correlated with *Cdk11b* (bicor = 0.59, p-value 1.53 x 10^−10^) and *Cdk11b* was controlled by the *Myh14* locus (rs32804715, p-value = 9.08 x 10^−6^), which implicated *Cdk11b*, a member of the cyclin-dependent kinase family known to be a negative regulator of normal cell cycle progression, as a potential mediator of *Myh14*.

Moreover, we showed that ISO-treated *Myh14* deficient heart demonstrated disrupted assembly of intercalated discs involving β-catenin, which is a dual function protein that provides cell-cell connections at the adherens junctions in the intercalated disc, to facilitate mechanical and electrical coupling between adjacent cardiomyocytes, and regulates gene transcription as a transcriptional coactivator, raising the possibility that disordered β-catenin localization in the intercalated disk due to *Myh14* deficiency could have direct consequences on β-catenin signaling. In fact, altered intercalated disc architecture in cardiac muscle hypertrophy and HF has previously been associated with altered β-catenin abundance [59], subcellular localization [60], and canonical Wnt/β-catenin/TCF signaling. Interestingly, transcription factors FOXO and TCF are known to compete to interact with β-catenin [61]. Our experimental results showed that *Myh14* is important in the maintenance of normal cardiac structure under ISO stress and that the loss of *Myh14* exacerbates the hypertrophic response to ISO. Further understanding of the crosstalk between *Myh14*, β-catenin, and *Foxo1*, will likely reveal important mechanisms regarding how *Myh14* deficiency leads to cardiac hypertrophy under ISO stress.

Cardiac remodeling is one of the most important prognostic determinants of clinical HF. Our study results provide an important resource to the HF research community and highlight the strength of a systems approach to studying HF. The integration of high throughput molecular phenotypes, such as genomic and transcriptomic data, provides a means to identify novel candidate causal genes and a powerful alternative to human studies to understand the complex interactions underpinning phenotypic variation of cardiac remodeling. Similar to human GWAS, many of our lead SNPs lie in noncoding regions. Future directions will include sequence-based computational approaches to systematically prioritize functional regulatory variants. Future insights in how common genetic variations in a population modify HF progression will further shed light on genetic risk profiling, gene-gene and gene-environment interactions as well as the design of personalized therapies for HF patients.

## Supporting Information

S1 Fig Baseline and week 3 echocardiographic measures in control mice. (A) Baseline and week 3 echocardiographic measures in control mice were significantly correlated. (B) Bland-Altman plots demonstrated agreement between baseline and week 3 echocardiographic measures in control mice. IVSd represents the interventricular septal wall thickness during diastole. LVIDd represents the left ventricular internal dimension during diastole. LVM represents left ventricular mass. FS represents fractional shortening. Each data point represents a mouse in the control group.
(PDF)

S2 Fig Association results from EMMA and FaST-LMM. The manhattan plots for the change in week 1 IVSd show that association p-values by EMMA and FaST-LMM analyses were similar.
(PDF)

S3 Fig Left ventricular weight and echocardiographic estimates of left ventricular mass were significantly and highly correlated. (A) LVM at baseline versus control LV. (B) LVM versus LV after ISO for 3 weeks. (C) The change in LVM from baseline to week 3 of ISO versus the difference in the strain-averaged LV weights between control and ISO. Bland-Altman plots (bottom row) showing the agreement between LV and LVM are provided below. (D) Comparison of LV and LVM phenotypic spectra between control and isoproterenol hearts at week 3.
Each data point represents a mouse strain.
(PDF)

S4 Fig Variation in the echocardiographic measures of cardiac structure and function among mouse strains. (A) Black bars represent measurements under the baseline condition. White bars represent measurements after 3 weeks of continuous ISO infusion. Data presented in alphabetical order of strain names. Error bars represent the standard errors of the means. (B) Gray bars represent the differences in measurements between baseline and week 3 of continuous ISO infusion in ranked order.
(PDF)

S5 Fig The spectrum of LVM among C57BL/6, DBA/2, and BXD recombinant inbred strains. The baseline LVM and week 3 LVM for each of the C57BL/6, DBA/2, and BXD RI strains (top). The change in LVM at week 3 for C57BL/6, DBA/2, and BXD RI strains (bottom). Error bars represent the standard error of the means.
(PDF)

S6 Fig Relationships between baseline body weight, heart rate and echocardiographic measures. (A) Relationships between echocardiographic measures and baseline body weight. BBW represents baseline body weight. (B) Relationships between echocardiographic measures and heart rate at corresponding time points. HR represents heart rate. (C) Relationships between HR across time points. Each data point represents a mouse strain.
(PDF)

S7 Fig Relative magnitude of heritability estimates. Line repeatability and marker-based h2 estimates for echo measures IVSd, LVIDd, LVM, and FS at baseline, week 1, week 2, and week 3 of ISO are compared.
(PDF)

S8 Fig Fine mapping of LVM at chromosome 4 across time points. A. Regional plot for LVM hypertrophy at week 1. B. Regional plot for LVM hypertrophy at week 2. C. Regional plot for ISO-treated LV hypertrophy at week 3. D. Regional plot for the ISO-treated *Klf4* expression (ILMN_1221264) around SNP rs27794497 (purple).
(PDF)

S9 Fig Correlation between *Klf4* transcript levels and week 3 LVM. Color circles represent SNP genotype at rs27811538. Bicor correlation and p-values are provided above. Pearson correlation between LVM and *Klf4* are as follows: Control ILMN_1241903 r = -0.28 p-value = 0.01, ISO ILMN_1241903 r = -0.21 p-value = 0.07; Control ILMN_1221264 r = -0.23 p-value = 0.04, ISO ILMN_1221264 r = -0.21 and p-value = 0.07.
(PDF)

S10 Fig Comparative analysis of classical inbred, BXD, and all HMDP strains. The genome-wide and chromosome 9 manhattan plots for the change in week 1 IVSd analyzed using the classical inbred strains only (A) and all of the HMDP strains (C). The genome-wide and chromosome 9 linkage maps for the change in week 1 IVSd analyzed using the BXD recombinant inbred strains only (B).
(PDF)

S11 Fig Comparative analysis of classical inbred, BXD, and all HMDP strains. The genome-wide and chromosome 7 manhattan plots for the change in week 3 LVM analyzed using the classical inbred strains only (A) and all of the HMDP strains (C). The genome-wide and chromosome 7 linkage maps for the change in week 3 LVM analyzed using the BXD recombinant inbred strains only (B).
(PDF)

S1 Table The reproducibility of echocardiographic measures in control mice. (PDF)

S2 Table Differentially regulated genes by isoproterenol treatment. A. Significantly up- or down-regulated cardiac genes by isoproterenol treatment. B. DAVID gene ontology analysis of up-regulated cardiac genes. C. DAVID gene ontology analysis of down-regulated cardiac genes. D. Summary of DAVID gene ontology analysis of cardiac genes correlated to echocardiographic measures. DAVID gene ontology analysis was performed based on top 1000 correlated cardiac genes for each echocardiographic measures either at baseline or at wee 3 of isoproterenol. Gene clusters exceeding nominal p-value 0.05 are shown.
(XLSX)

S3 Table The number of births by gender and genotypes in *Myh14*^+/-^ crosses. (PDF)

S4 Table Study sample characteristics of the HMDP across isoproterenol treatment time points. (PDF)

S5 Table Echcardiographic measures of cardiac structure and function at different isoproterenol treatment time points by HMDP mouse strains. (PDF)

S6 Table Summary of association loci and loci gene differential expression, cis-eQTL, correlation to trait and sequence variations. A. Summary of genetic loci by trait. Weight traits such as total heart (TH), left ventricle (LV) and right ventricle (RV) weights are denoted by the prefix wt. Echo traits are denoted by the prefix w1 for week 1, w2 for week 2, and w3 for week 3 followed by echo for measurement or delta for the difference compared to baseline at the corresponding time point. Genomic coordinates were listed in mm9. B. Genes within the association loci were queried using a custom computational pipeline to examine the presence of differential expression with isoproterenol, cis-eQTL, correlation to trait of interest and sequence or structural variations.
(XLSX)

S7 Table SNPs with association p-value < 0.001. Select SNPs from our analysis that exceed the association p-value threshold of < 0.001.
(XLSX)

S8 Table Human and mouse cardiovascular GWAS overlap genes. (PDF)

S9 Table *Myh14* cis-eQTL across multiples tissues in different HMDP datasets. (PDF)

S10 Table *Myh14* correlation to heart weight in different HMDP datasets. (PDF)
