# Supplementary material for: Genetic Dissection of Cardiac Remodeling in an Isoproterenol-Induced Heart Failure Mouse Model
Source: PLoS Genet. 2016 Jul 6;12(7):e1006038. doi: 10.1371/journal.pgen.1006038 (PMC4934852; doi:10.1371/journal.pgen.1006038)
Supplement: S1 Fig — (A) Baseline and week 3 echocardiographic measures in control mice were significantly correlated. (B) Bland-Altman plots demonstrated agreement between baseline and week 3 echocardiographic measures in control mice. IVSd represents the interventricular septal wall thickness during diastole. LVIDd represents the left ventricular internal dimension during diastole. LVM represents left ventricular mass. FS represents fractional shortening. Each data point represents a mouse in the control group. (PDF) [file pgen.1006038.s001.pdf]

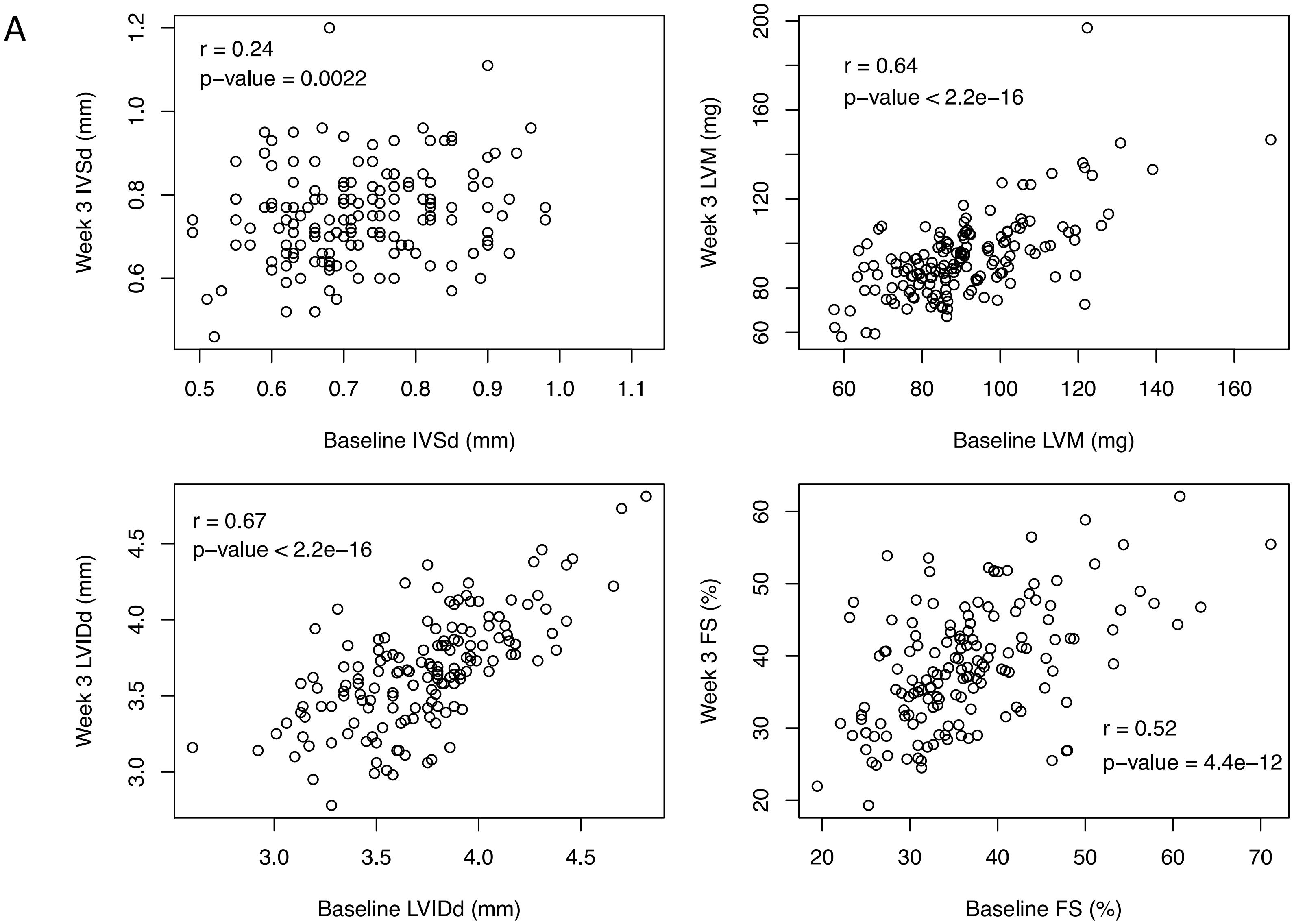

S1 Fig. Baseline and week 3 echocardiographic measures in control mice

(A) Baseline and week 3 echocardiographic measures in control mice were significantly correlated. IVSd represents the interventricular septal wall thickness during diastole. LVIDd represents the left ventricular internal dimension during diastole. LVM represents left ventricular mass. FS represents fractional shortening. Each data point represents a mouse in the control group.

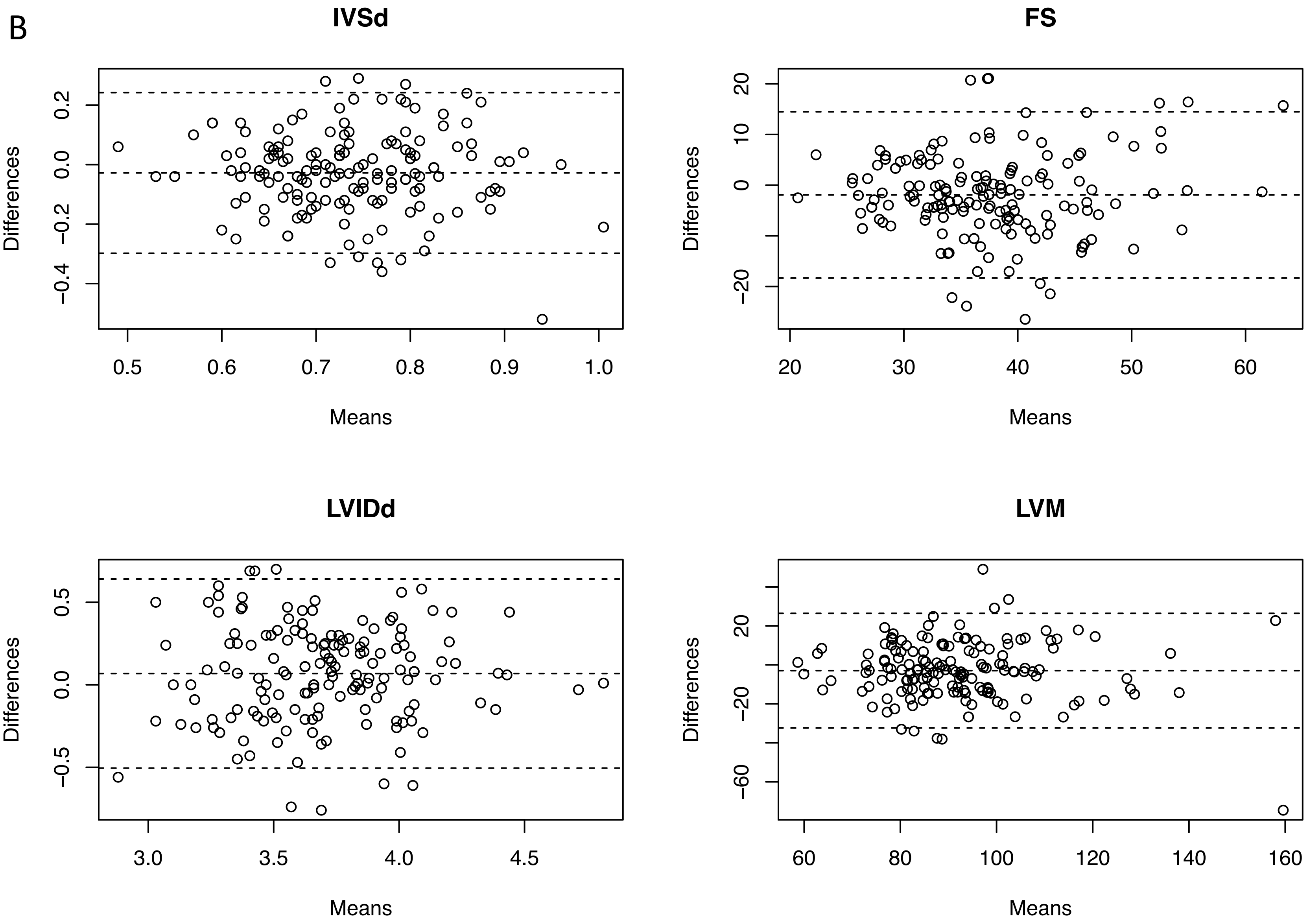

S1 Fig. Baseline and week 3 echocardiographic measures in control mice

(B) Bland-Altman plots demonstrated agreement between baseline and week 3 echocardiographic measures in control mice.
