# Supplementary material for: Genetic Dissection of Cardiac Remodeling in an Isoproterenol-Induced Heart Failure Mouse Model
Source: PLoS Genet. 2016 Jul 6;12(7):e1006038. doi: 10.1371/journal.pgen.1006038 (PMC4934852; doi:10.1371/journal.pgen.1006038)
Supplement: S2 Fig — The manhattan plots for the change in week 1 IVSd show that association p-values by EMMA and FaST-LMM analyses were similar. (PDF) [file pgen.1006038.s002.pdf]

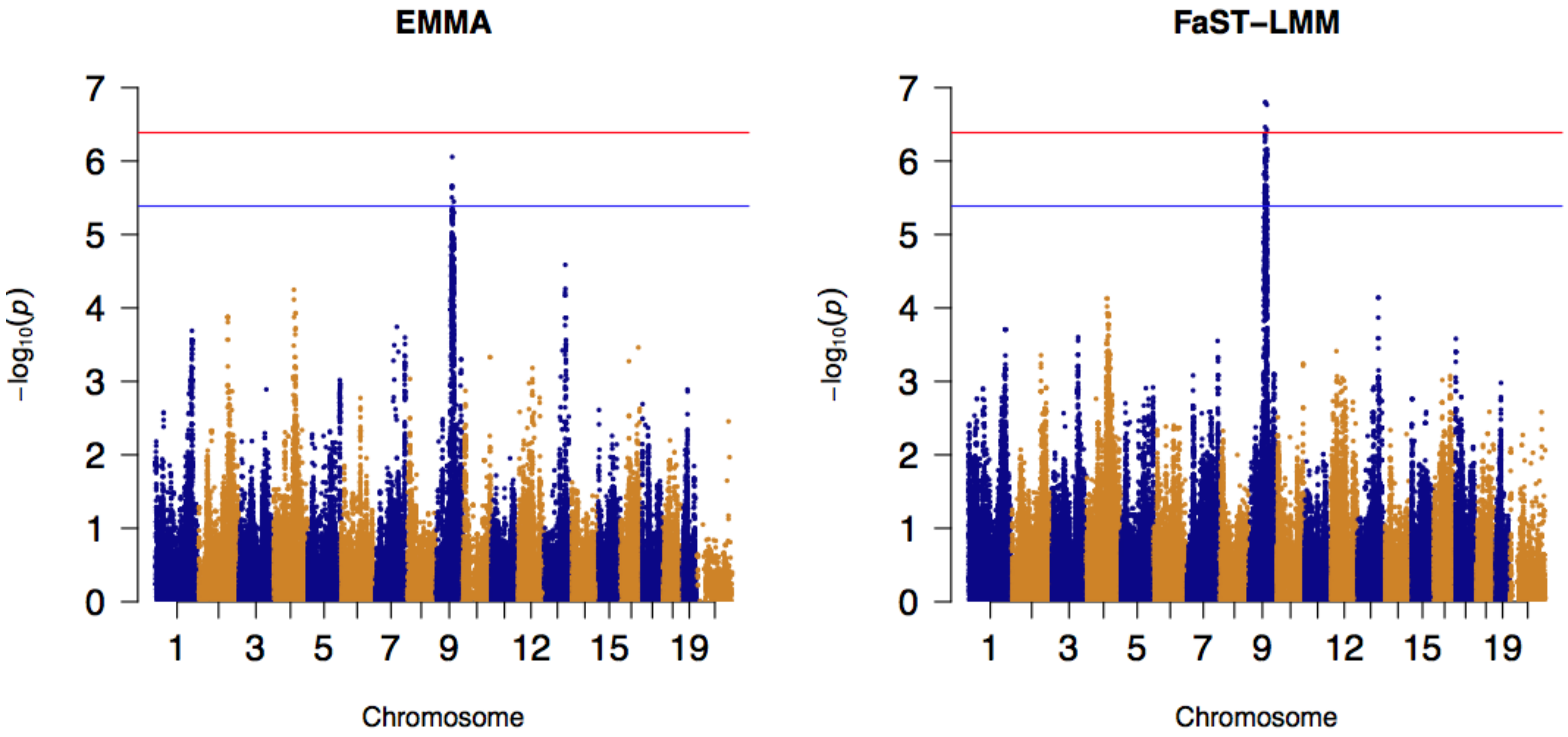

S2 Fig. Association results from EMMA and FaST-LMM
The manhattan plots for the change in week 1 IVSd show that association p-values by EMMA and FaST-LMM analyses were similar.
