# Supplementary material for: Genetic Dissection of Cardiac Remodeling in an Isoproterenol-Induced Heart Failure Mouse Model
Source: PLoS Genet. 2016 Jul 6;12(7):e1006038. doi: 10.1371/journal.pgen.1006038 (PMC4934852; doi:10.1371/journal.pgen.1006038)
Supplement: S3 Fig — (A) LVM at baseline versus control LV. (B) LVM versus LV after ISO for 3 weeks. (C) The change in LVM from baseline to week 3 of ISO versus the difference in the strain-averaged LV weights between control and ISO. Bland-Altman plots (bottom row) showing the agreement between LV and LVM are provided below. (D) Comparison of LV and LVM phenotypic spectra between control and isoproterenol hearts at week 3. Each data point represents a mouse strain. (PDF) [file pgen.1006038.s003.pdf]

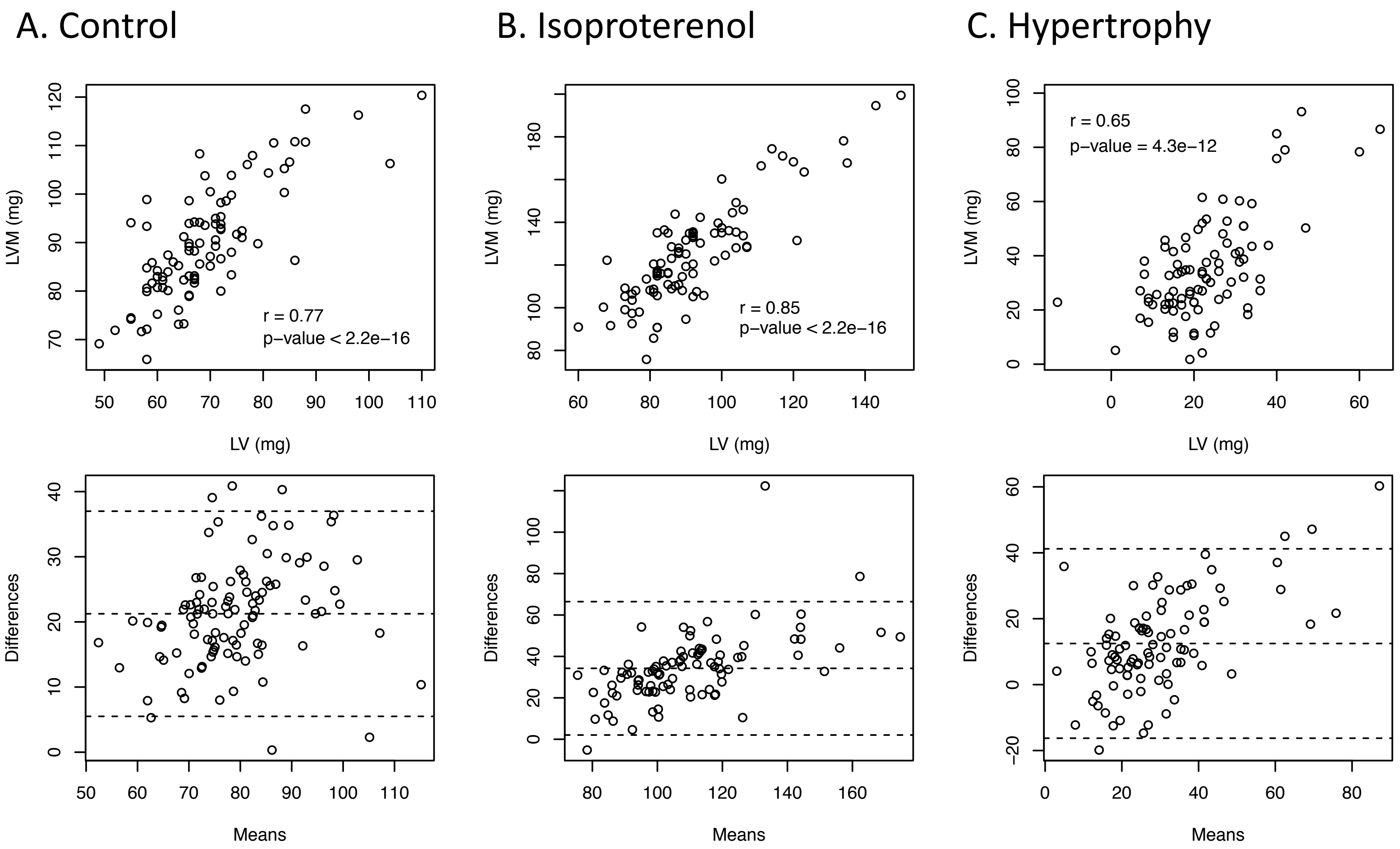

S3 Fig. Left ventricular weight and echocardiographic estimates of left ventricular mass were significantly and highly correlated

(A) LVM at baseline versus control LV. (B) LVM versus LV after ISO for 3 weeks. (C) The change in LVM from baseline to week 3 of ISO versus the difference in the strain-averaged LV weights between control and ISO. Bland-Altman plots (bottom row) showing the agreement between LV and LVM are provided below. Each data point represents a mouse strain.

D

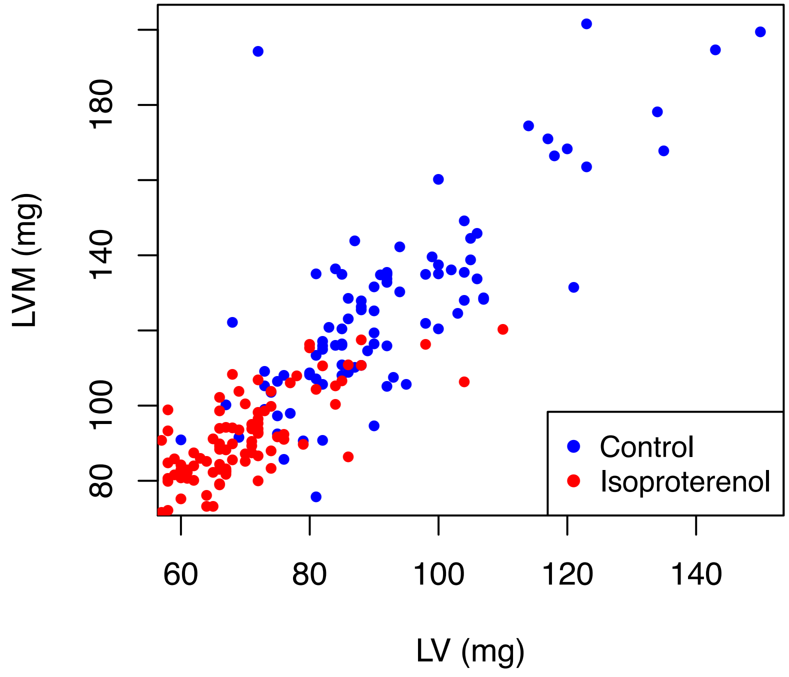

S3 Fig. Left ventricular weight and echocardiographic estimates of left ventricular mass were significantly and highly correlated

(D) Comparison of LV and LVM phenotypic spectra between control and isoproterenol hearts at week 3.
