# Supplementary material for: Genetic Dissection of Cardiac Remodeling in an Isoproterenol-Induced Heart Failure Mouse Model
Source: PLoS Genet. 2016 Jul 6;12(7):e1006038. doi: 10.1371/journal.pgen.1006038 (PMC4934852; doi:10.1371/journal.pgen.1006038)
Supplement: S4 Fig — (A) Black bars represent measurements under the baseline condition. White bars represent measurements after 3 weeks of continuous ISO infusion. Data presented in alphabetical order of strain names. Error bars represent the standard errors of the means. (B) Gray bars represent the differences in measurements between baseline and week 3 of continuous ISO infusion in ranked order. (PDF) [file pgen.1006038.s004.pdf]

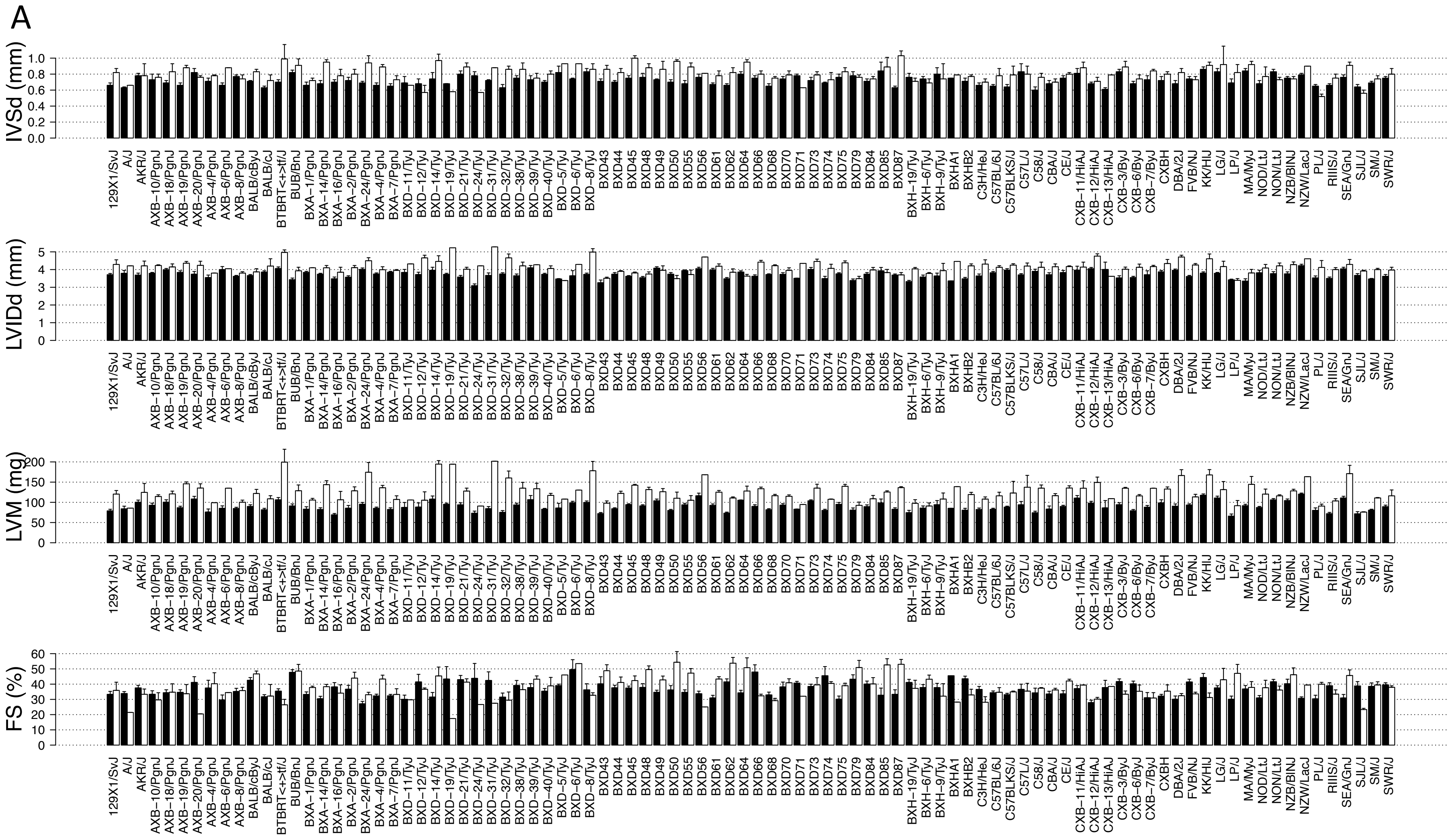

S4 Fig. Variation in the echocardiographic measures of cardiac structure and function among mouse strains. (A) Black bars represent measurements under the baseline condition. White bars represent measurements after 3 weeks of continuous ISO infusion. Data presented in alphabetical order of strain names. Error bars represent the standard errors of the means.

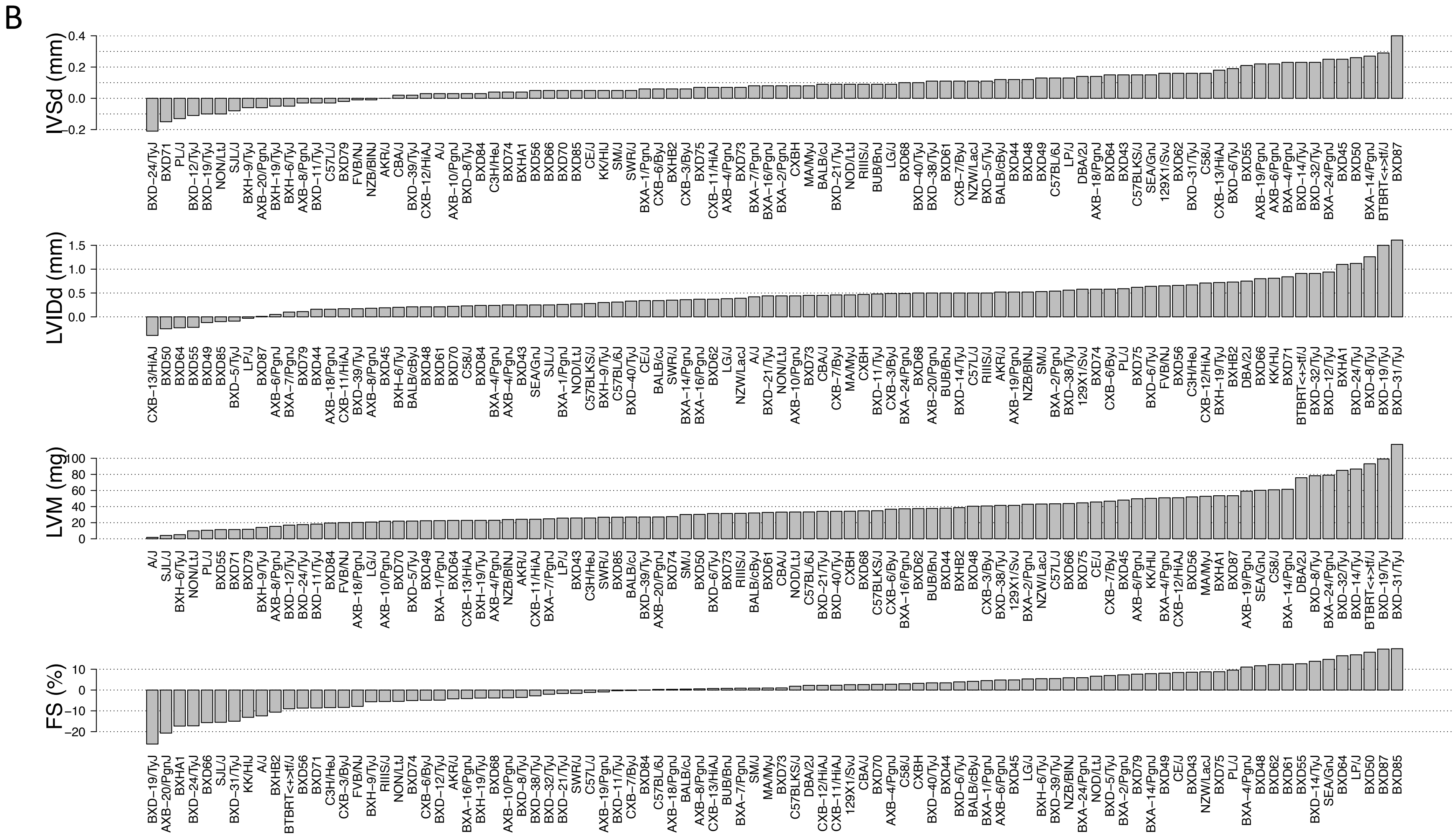

S4 Fig. Variation in the echocardiographic measures of cardiac structure and function among mouse strains. (B) Gray bars represent the differences in measurements between baseline and week 3 of continuous ISO infusion in ranked order.
