# Supplementary material for: Genetic Dissection of Cardiac Remodeling in an Isoproterenol-Induced Heart Failure Mouse Model
Source: PLoS Genet. 2016 Jul 6;12(7):e1006038. doi: 10.1371/journal.pgen.1006038 (PMC4934852; doi:10.1371/journal.pgen.1006038)
Supplement: S5 Fig — The baseline LVM and week 3 LVM for each of the C57BL/6, DBA/2, and BXD RI strains (top). The change in LVM at week 3 for C57BL/6, DBA/2, and BXD RI strains (bottom). Error bars represent the standard error of the means. (PDF) [file pgen.1006038.s005.pdf]

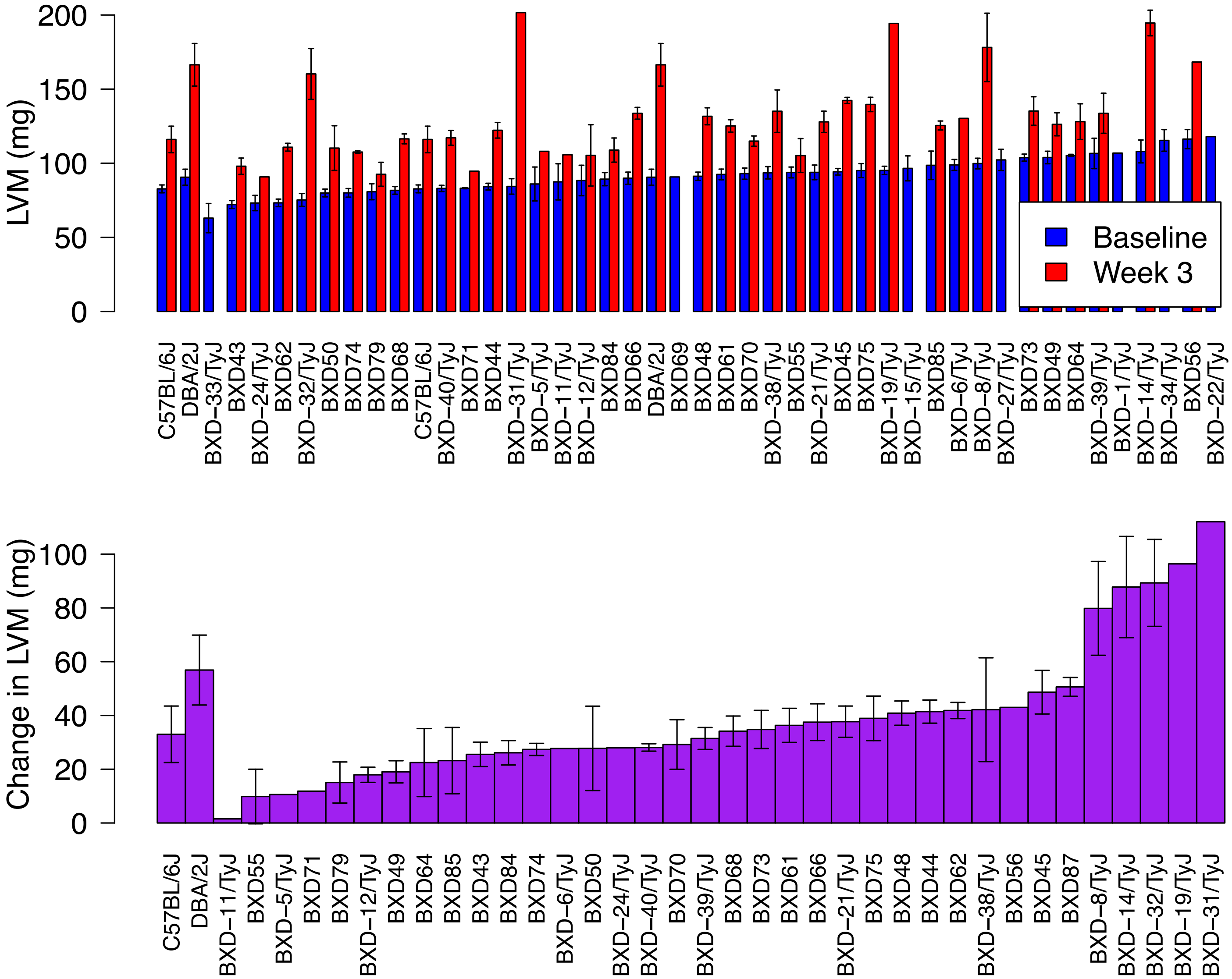

S5 Fig. The spectrum of LVM among C57BL/6, DBA/2, and BXD recombinant inbred strains
The baseline LVM and week 3 LVM for each of the C57BL/6, DBA/2, and BXD RI strains (top). The change in LVM at week 3 for C57BL/6, DBA/2, and BXD RI strains (bottom). Error bars represent the standard error of the means.
