# Supplementary material for: Genetic Dissection of Cardiac Remodeling in an Isoproterenol-Induced Heart Failure Mouse Model
Source: PLoS Genet. 2016 Jul 6;12(7):e1006038. doi: 10.1371/journal.pgen.1006038 (PMC4934852; doi:10.1371/journal.pgen.1006038)
Supplement: S6 Fig — (A) Relationships between echocardiographic measures and baseline body weight. BBW represents baseline body weight. (B) Relationships between echocardiographic measures and heart rate at corresponding time points. HR represents heart rate. (C) Relationships between HR across time points. Each data point represents a mouse strain. (PDF) [file pgen.1006038.s006.pdf]

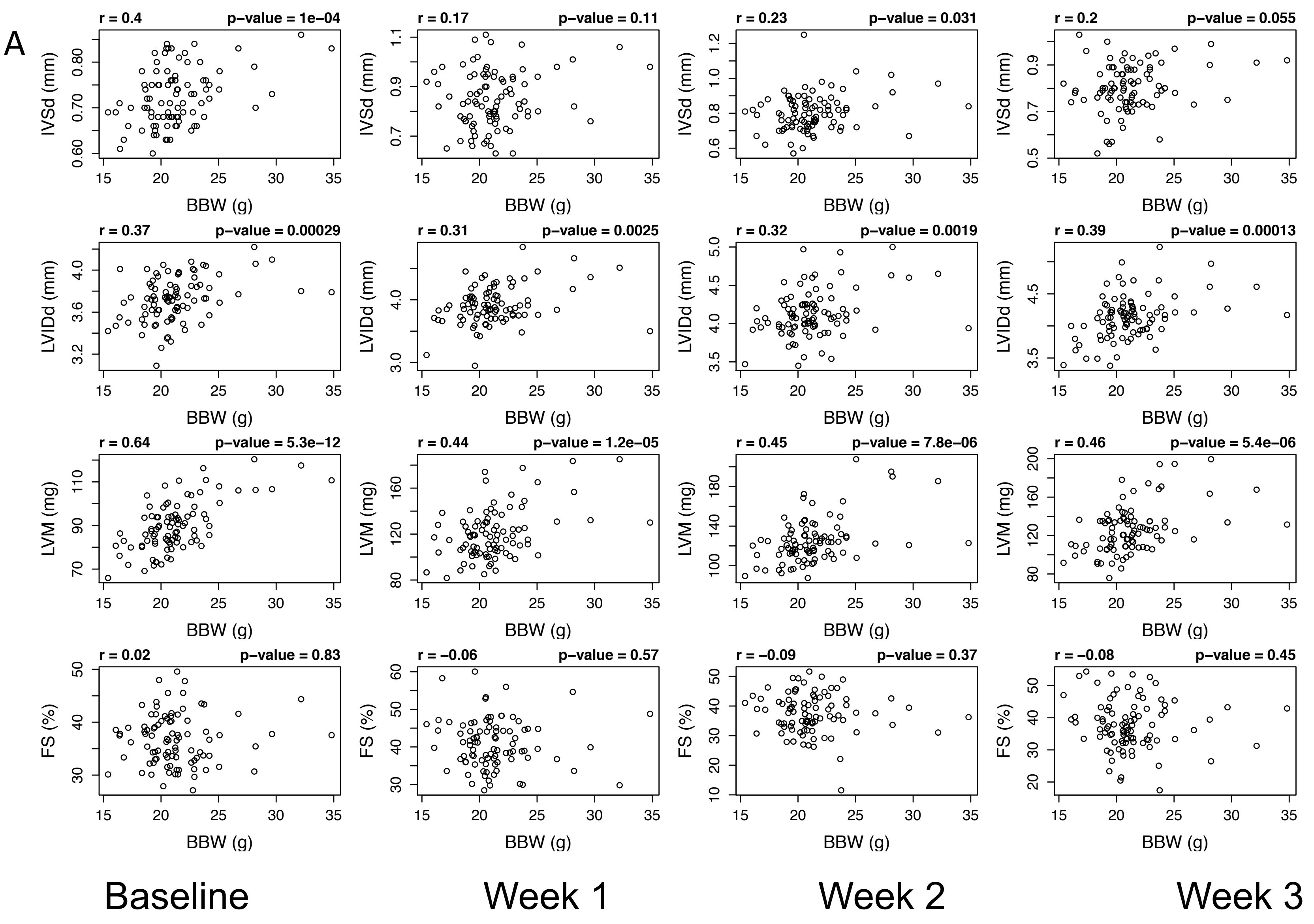

S6 Fig. Relationships between baseline body weight, heart rate and echocardiographic measures

(A) Relationships between echocardiographic measures and baseline body weight. BBW represents baseline body weight. Each data point represents a mouse strain.

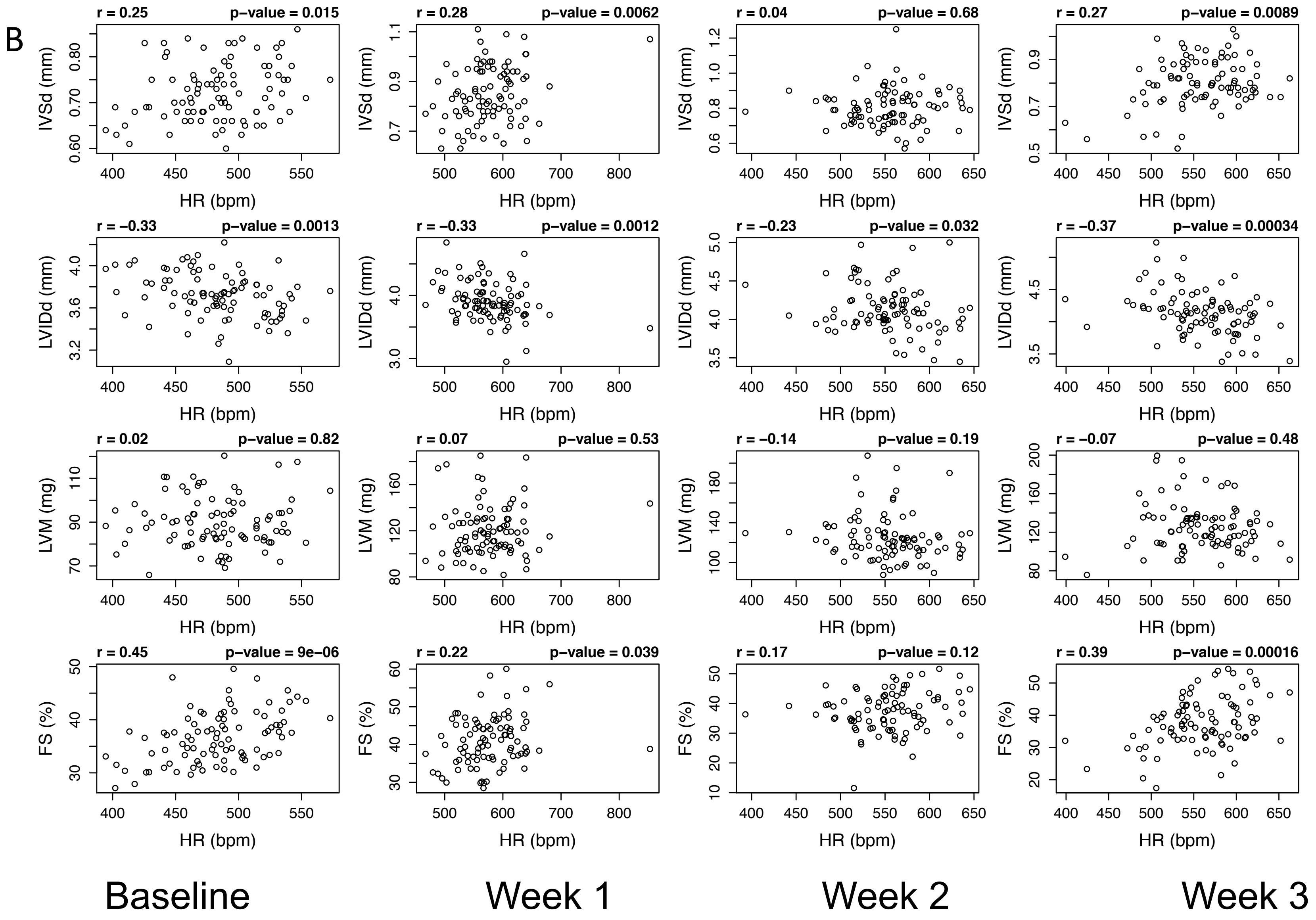

S6 Fig. Relationships between baseline body weight, heart rate and echocardiographic measures

(B) Relationships between echocardiographic measures and heart rate at corresponding time points. HR represents heart rate. Each data point represents a mouse strain.

C

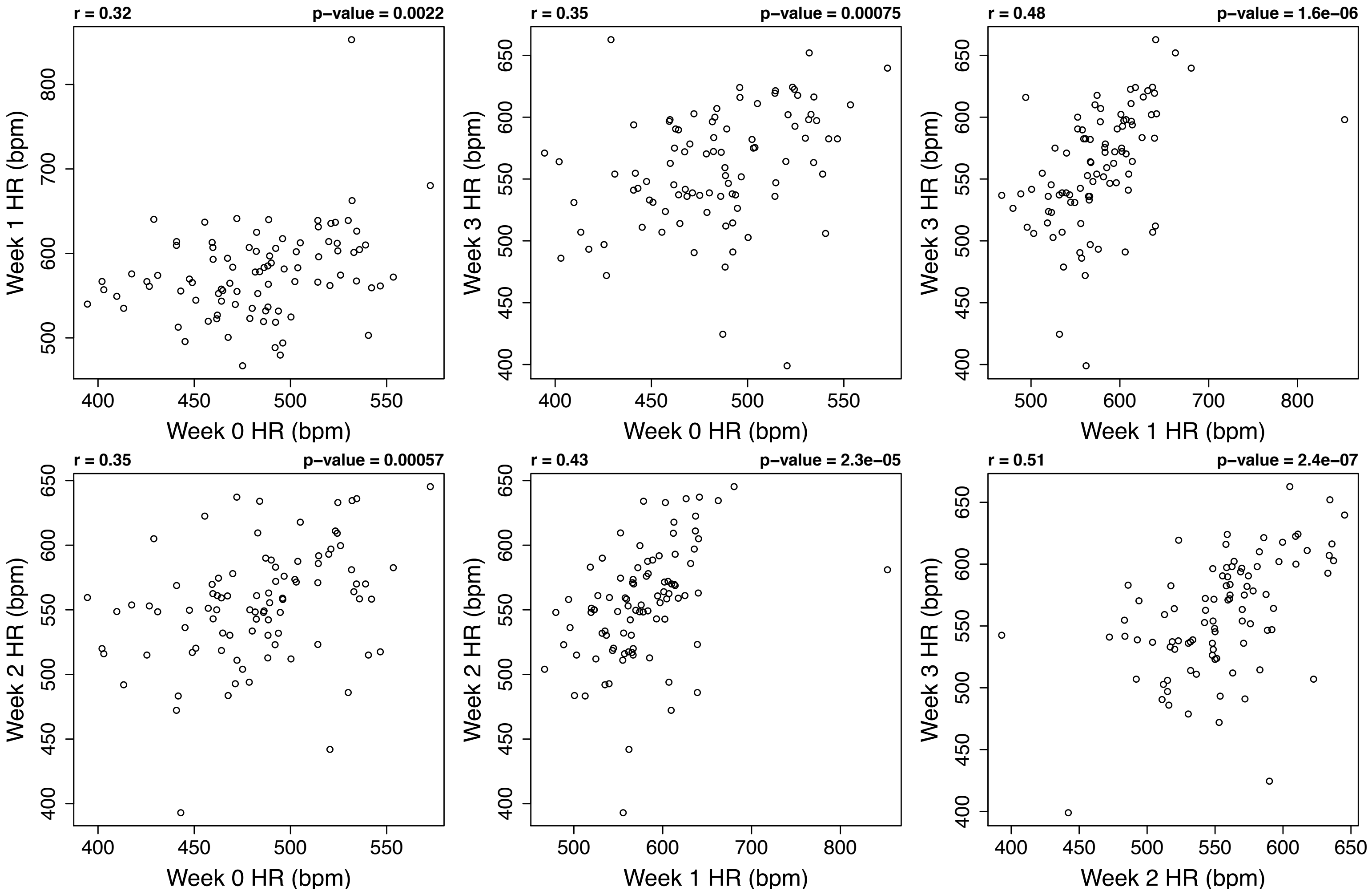

S6 Fig. Relationships between baseline body weight, heart rate and echocardiographic measures

(C) Relationships between heart rates across time points. HR represents heart rate. Each data point represents a mouse strain.
