# Supplementary material for: Genetic Dissection of Cardiac Remodeling in an Isoproterenol-Induced Heart Failure Mouse Model
Source: PLoS Genet. 2016 Jul 6;12(7):e1006038. doi: 10.1371/journal.pgen.1006038 (PMC4934852; doi:10.1371/journal.pgen.1006038)
Supplement: S7 Fig — Line repeatability and marker-based h2 estimates for echo measures IVSd, LVIDd, LVM, and FS at baseline, week 1, week 2, and week 3 of ISO are compared. (PDF) [file pgen.1006038.s007.pdf]

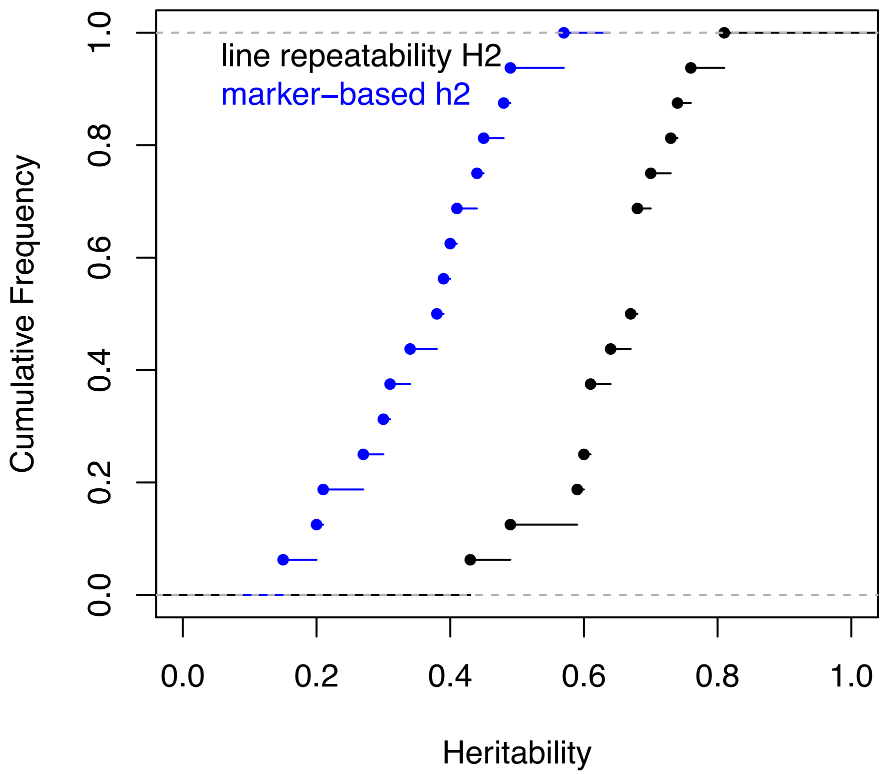

S7 Fig. Relative magnitude of heritability estimates
Line repeatability and and marker-based h2 estimates for echo measures IVSd, LVIDd, LVM, and FS at baseline, week 1, week 2, and week 3 of ISO are compared.
