# Supplementary material for: Genetic Dissection of Cardiac Remodeling in an Isoproterenol-Induced Heart Failure Mouse Model
Source: PLoS Genet. 2016 Jul 6;12(7):e1006038. doi: 10.1371/journal.pgen.1006038 (PMC4934852; doi:10.1371/journal.pgen.1006038)
Supplement: S8 Fig — A. Regional plot for LVM hypertrophy at week 1. B. Regional plot for LVM hypertrophy at week 2. C. Regional plot for ISO-treated LV hypertrophy at week 3. D. Regional plot for the ISO-treated Klf4 expression (ILMN_1221264) around SNP rs27794497 (purple). (PDF) [file pgen.1006038.s008.pdf]

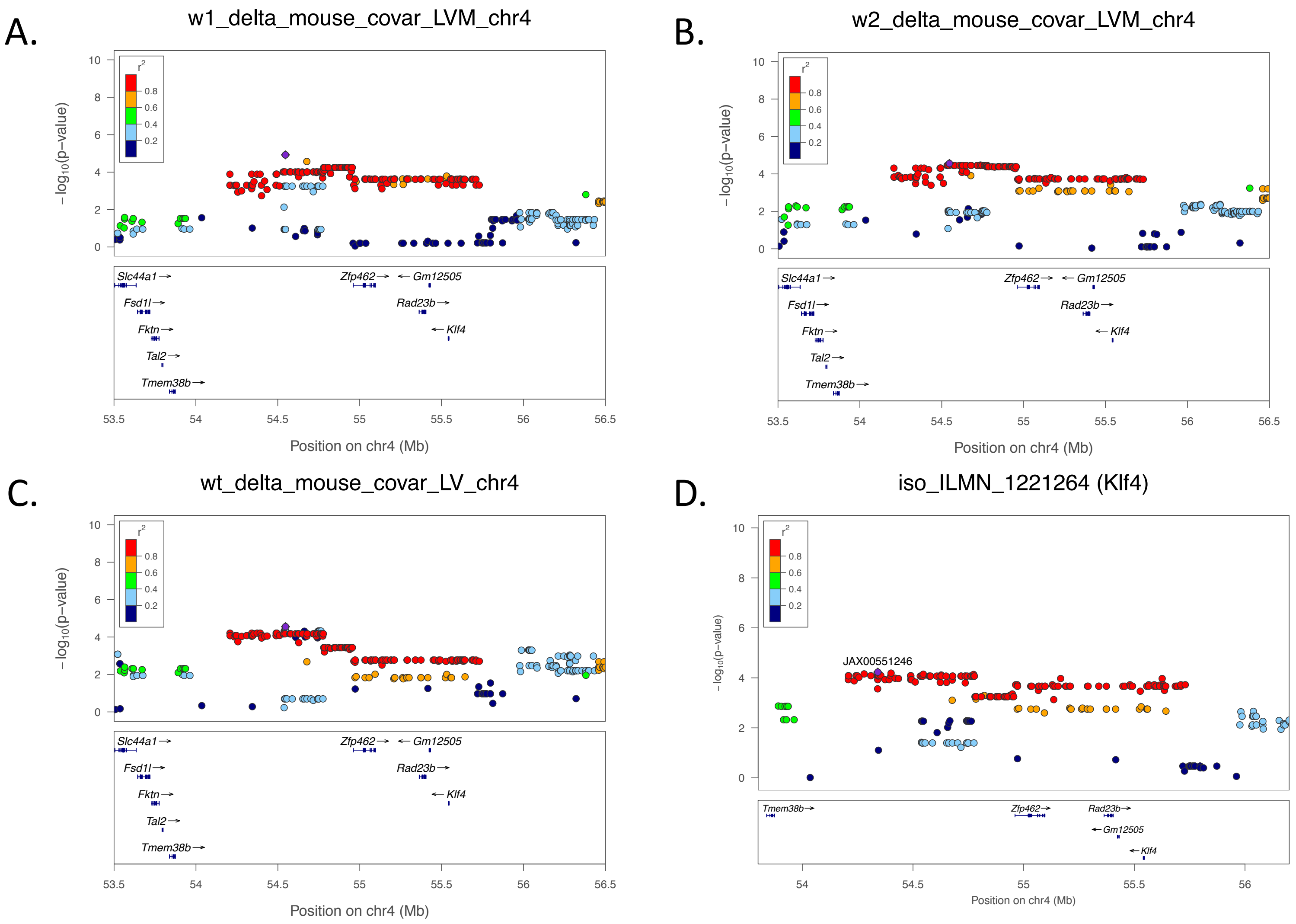

S8 Fig. Fine mapping of LVM at chromosome 4 across time points

A. Regional plot for LVM hypertrophy at week 1. B. Regional plot for LVM hypertrophy at week 2. C. Regional plot for ISO-treated LV hypertrophy at week 3. D. Regional plot for the ISO-treated *Klf4* expression (ILMN_1221264) around SNP rs27794497 (purple).
