# Supplementary material for: Genetic Dissection of Cardiac Remodeling in an Isoproterenol-Induced Heart Failure Mouse Model
Source: PLoS Genet. 2016 Jul 6;12(7):e1006038. doi: 10.1371/journal.pgen.1006038 (PMC4934852; doi:10.1371/journal.pgen.1006038)
Supplement: S9 Fig — Color circles represent SNP genotype at rs27811538. Bicor correlation and p-values are provided above. Pearson correlation between LVM and Klf4 are as follows: Control ILMN_1241903 r = -0.28 p-value = 0.01, ISO ILMN_1241903 r = -0.21 p-value = 0.07; Control ILMN_1221264 r = -0.23 p-value = 0.04, ISO ILMN_1221264 r = -0.21 and p-value = 0.07. (PDF) [file pgen.1006038.s009.pdf]

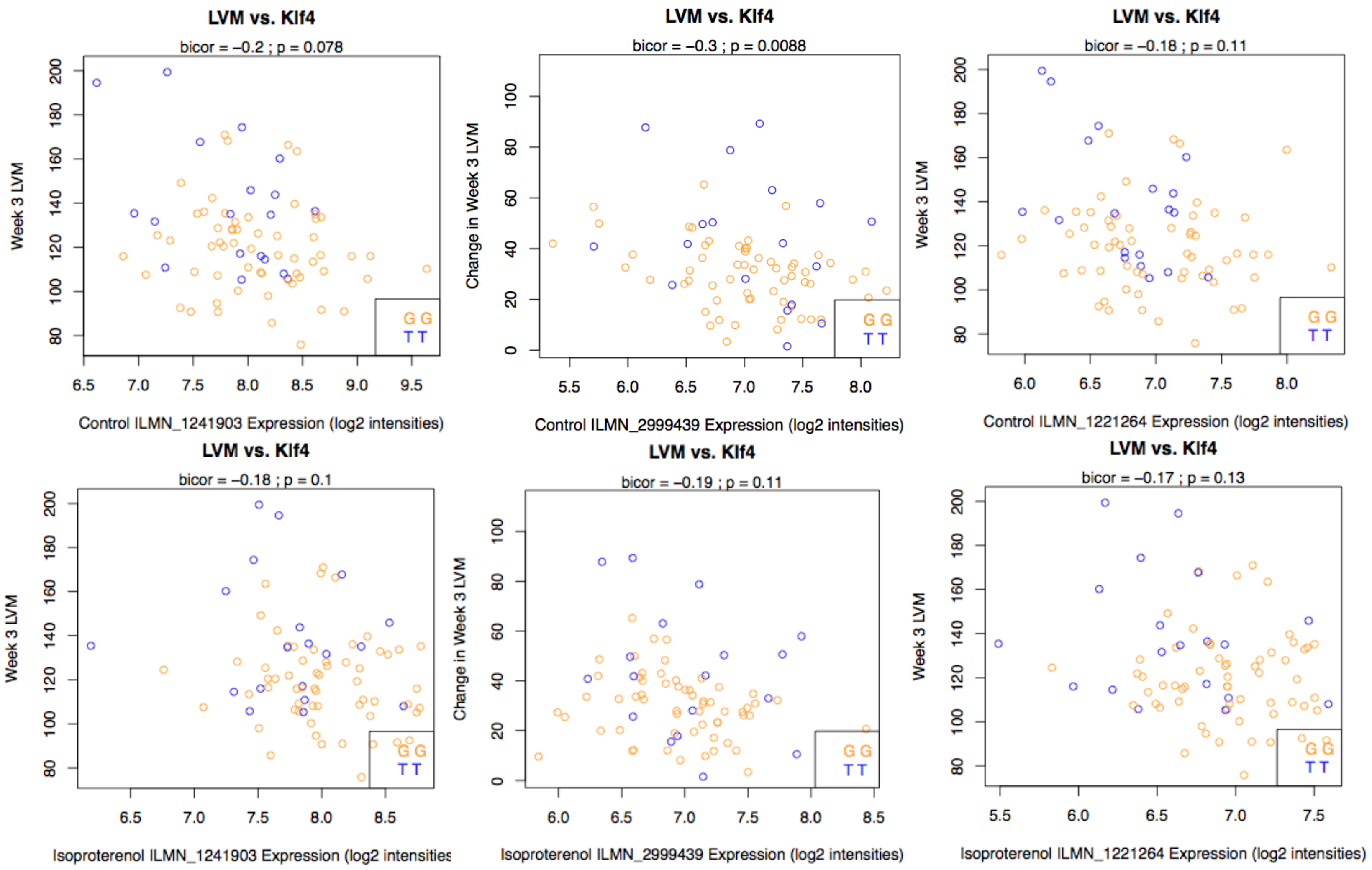

S9 Fig. Correlation between *Klf4* transcript levels and week 3 LVM

Color circles represent SNP genotype at rs27811538. Bicor correlation and p-values are provided above. Pearson correlation between LVM and *Klf4* are as follows: Control ILMN_1241903 r = -0.28 p-value = 0.01, ISO ILMN_1241903 r = -0.21 p-value = 0.07; Control ILMN_1221264 r = -0.23 p-value = 0.04, ISO ILMN_1221264 r = -0.21 and p-value = 0.07.
