# Supplementary material for: Genetic Dissection of Cardiac Remodeling in an Isoproterenol-Induced Heart Failure Mouse Model
Source: PLoS Genet. 2016 Jul 6;12(7):e1006038. doi: 10.1371/journal.pgen.1006038 (PMC4934852; doi:10.1371/journal.pgen.1006038)
Supplement: S11 Fig — The genome-wide and chromosome 7 manhattan plots for the change in week 3 LVM analyzed using the classical inbred strains only (A) and all of the HMDP strains (C). The genome-wide and chromosome 7 linkage maps for the change in week 3 LVM analyzed using the BXD recombinant inbred strains only (B). (PDF) [file pgen.1006038.s011.pdf]

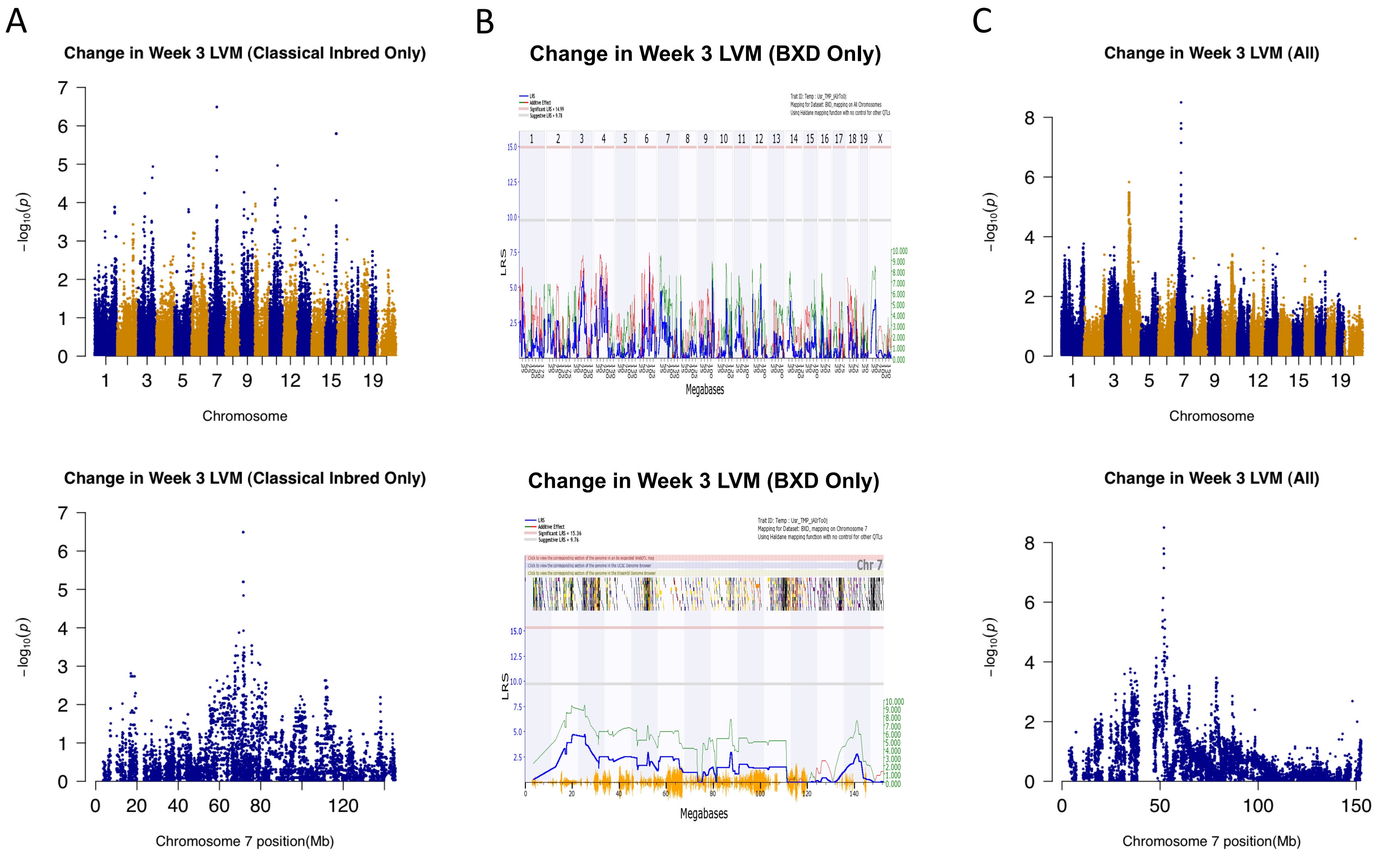

S11 Fig. Comparative analysis of classical inbred, BXD, and all HMDP strains

The genomewide and chromosome 7 manhattan plots for the change in week 3 LVM analyzed using the classical inbred strains only (A) and all of the HMDP strains (C). The genomewide and chromosome 7 linkage maps for the change in week 3 LVM analyzed using the BXD recombinant inbred strains only (B).
