# Supplementary material for: Genetic Dissection of Cardiac Remodeling in an Isoproterenol-Induced Heart Failure Mouse Model
Source: PLoS Genet. 2016 Jul 6;12(7):e1006038. doi: 10.1371/journal.pgen.1006038 (PMC4934852; doi:10.1371/journal.pgen.1006038)
Supplement: S1 Table — (PDF) [file pgen.1006038.s012.pdf]

S1 Table. The reproducibility of echocardiographic measures in control mice.

	Baseline	Week 3	p-value
IVSd (mm)	0.73	0.75	0.725
LVIDd (mm)	3.75	3.68	0.019
LVM (mg)	92.3	93.5	0.345
FS (%)	36.5	38.8	0.002

Echocardiograms in control mice from 70 strains were performed at baseline and at week 3 time points. The mean values at each time point were compared using a paired t-test.
