# Supplementary material for: Genetic Dissection of Cardiac Remodeling in an Isoproterenol-Induced Heart Failure Mouse Model
Source: PLoS Genet. 2016 Jul 6;12(7):e1006038. doi: 10.1371/journal.pgen.1006038 (PMC4934852; doi:10.1371/journal.pgen.1006038)
Supplement: S3 Table — (PDF) [file pgen.1006038.s014.pdf]

S3 Table. The number of births by gender and genotypes in *Myh14*$^{+/-}$ crosses.

Sex	HET	KO	WT
Female	49	23	23
Male	33	23	18

Chi-Square test p = 0.13
