# Supplementary material for: Genetic Dissection of Cardiac Remodeling in an Isoproterenol-Induced Heart Failure Mouse Model
Source: PLoS Genet. 2016 Jul 6;12(7):e1006038. doi: 10.1371/journal.pgen.1006038 (PMC4934852; doi:10.1371/journal.pgen.1006038)
Supplement: S4 Table — (PDF) [file pgen.1006038.s015.pdf]

S4 Table. Study sample characteristics of the HMDP across isoproterenol treatment time points.

	Baseline	Week 1	Week 2	Week 3
IVSd (mm)	0.72 ± 0.06	0.85 ± 0.11	0.80 ± 0.10	0.80 ± 0.10
LVIDd (mm)	3.72 ± 0.21	3.91 ± 0.30	4.13 ± 0.30	4.14 ± 0.34
LVM (mg)	89.9 ± 11.4	120.2 ± 21.8	124.9 ± 22.7	125.8 ± 24.4
FS (%)	36.7 ± 4.8	40.9 ± 6.5	37.5 ± 6.7	37.7 ± 7.7

The population mean ± standard deviation of the 91 strains for which we have complete data across all time points are represented above.
