# Supplementary material for: Genetic Dissection of Cardiac Remodeling in an Isoproterenol-Induced Heart Failure Mouse Model
Source: PLoS Genet. 2016 Jul 6;12(7):e1006038. doi: 10.1371/journal.pgen.1006038 (PMC4934852; doi:10.1371/journal.pgen.1006038)
Supplement: S5 Table — (PDF) [file pgen.1006038.s016.pdf]

S5 Table. Echcardiographic measures of cardiac structure and function at different isoproterenol treatment time points by HMDP mouse strains.

A. IVSd (mm)

Strain	m.0	se.0	n.0	m.1	se.1	n.1	m.2	se.2	n.2	m.3	se.3	n.3
129X1/SvJ	0.66	0.03	11	0.85	0.05	7	0.75	0.06	5	0.82	0.05	4
A/J	0.63	0.01	7	0.67	0.06	3	0.6	0	2	0.66	NA	1
AKR/J	0.78	0.03	8	0.8	0.05	6	0.72	0.02	4	0.78	0.15	2
AXB-10/PgnJ	0.73	0.07	5	0.96	NA	1	0.67	NA	1	0.76	0.04	3
AXB-12/PgnJ	0.74	0.04	3	NA	NA	NA	NA	NA	NA	NA	NA	NA
AXB-13/PgnJ	0.68	NA	1	NA	NA	NA	NA	NA	NA	NA	NA	NA
AXB-18/PgnJ	0.69	0.03	8	0.68	0.06	4	0.95	0.02	3	0.83	0.1	3
AXB-19/PgnJ	0.66	0.03	10	0.8	0.08	3	0.93	0.19	3	0.88	0.03	3
AXB-20/PgnJ	0.82	0.05	5	0.95	0.05	2	0.76	0.09	2	0.76	0.02	2
AXB-4/PgnJ	0.71	0.04	3	0.91	0.13	2	0.67	0.01	2	0.78	0.01	2
AXB-6/PgnJ	0.66	0.03	2	0.63	NA	1	0.72	NA	1	0.88	NA	1
AXB-8/PgnJ	0.77	0.02	8	0.79	0.06	5	0.73	0.06	5	0.74	0.05	5
BALB/cByJ	0.71	0.01	6	0.86	0.03	3	0.88	0.05	3	0.83	0.03	3
BALB/cJ	0.63	0.02	18	0.7	0.05	6	0.7	0.05	4	0.72	0.07	3
BTBRT<+>tf/J	0.7	0.03	17	0.82	0.03	2	0.92	0.09	2	0.99	0.18	2
BUB/BnJ	0.82	0.03	11	0.92	0.1	5	0.98	0.04	4	0.91	0.08	5
BXA-1/PgnJ	0.66	0.04	5	0.78	0.01	2	0.75	0.04	2	0.72	0.03	2
BXA-11/PgnJ	0.7	NA	1	NA	NA	NA	NA	NA	NA	NA	NA	NA
BXA-12/PgnJ	0.66	0.04	4	0.79	NA	1	NA	NA	NA	NA	NA	NA
BXA-14/PgnJ	0.68	0.04	9	0.95	0.09	5	0.85	0.04	5	0.95	0.03	5
BXA-16/PgnJ	0.7	0.03	6	0.83	0.12	4	0.76	0.05	4	0.78	0.08	4
BXA-2/PgnJ	0.72	0.04	7	0.8	0.02	3	0.83	0.07	3	0.8	0.06	3
BXA-24/PgnJ	0.69	0.02	13	0.87	0.05	5	0.79	0.08	5	0.94	0.09	4
BXA-4/PgnJ	0.66	0.03	8	0.74	0.08	4	0.88	0.07	4	0.89	0.03	4
BXA-7/PgnJ	0.65	0.03	10	0.72	0.06	5	0.82	0.03	5	0.73	0.05	5
BXD-1/TyJ	0.89	NA	1	NA	NA	NA	NA	NA	NA	NA	NA	NA
BXD-11/TyJ	0.69	0.08	3	0.94	NA	1	0.9	NA	1	0.66	NA	1
BXD-12/TyJ	0.68	0.06	5	0.78	0.01	2	0.76	0.02	2	0.57	0.09	2
BXD-14/TyJ	0.74	0.08	4	0.94	0.04	3	1.04	0.06	3	0.97	0.08	3
BXD-15/TyJ	0.62	0.04	3	0.9	NA	1	NA	NA	NA	NA	NA	NA
BXD-19/TyJ	0.68	0	2	0.97	NA	1	0.72	NA	1	0.58	NA	1
BXD-21/TyJ	0.8	0.04	15	0.84	0.04	8	0.82	0.03	8	0.89	0.05	7
BXD-22/TyJ	0.93	NA	1	0.97	NA	1	0.49	NA	1	NA	NA	NA
BXD-24/TyJ	0.78	0.05	3	1.09	NA	1	0.57	NA	1	0.57	NA	1
BXD-27/TyJ	0.77	0.07	4	NA	NA	NA	NA	NA	NA	NA	NA	NA
BXD-31/TyJ	0.72	0.01	2	0.88	NA	1	NA	NA	NA	0.88	NA	1
BXD-32/TyJ	0.63	0.04	9	1.11	NA	1	0.79	NA	1	0.86	0.04	3
BXD-33/TyJ	0.71	0.19	2	NA	NA	NA	NA	NA	NA	NA	NA	NA
BXD-34/TyJ	0.74	0.07	7	NA	NA	NA	NA	NA	NA	NA	NA	NA
BXD-38/TyJ	0.75	0.03	10	0.99	0.05	2	0.79	0.15	2	0.86	0.08	2
BXD-39/TyJ	0.73	0.05	5	0.76	0.03	3	0.67	0.09	3	0.75	0.06	3
BXD-40/TyJ	0.7	0.02	21	0.76	0.04	6	0.93	0.1	6	0.8	0.04	5
BXD-5/TyJ	0.82	0.08	2	1.01	NA	1	0.85	NA	1	0.93	NA	1
BXD-6/TyJ	0.74	0.01	2	0.63	NA	1	0.77	NA	1	0.93	NA	1

BXD-8/TyJ	0.83	0.04	3	0.9	0.17	2	0.7	0.08	2	0.86	0.07	2
BXD43	0.71	0.03	7	0.88	0.04	5	0.9	0.04	5	0.86	0.05	5
BXD44	0.7	0.02	4	0.82	0.01	2	0.76	0.09	2	0.82	0.05	2
BXD45	0.75	0.03	7	0.87	0.05	2	0.9	0.08	2	1	0.03	2
BXD48	0.76	0.05	6	1.08	0.05	4	0.88	0.05	4	0.88	0.05	4
BXD49	0.73	0.01	7	0.89	0.01	3	0.84	0.08	3	0.86	0.1	3
BXD50	0.7	0.03	5	0.86	0.08	2	0.88	0	2	0.96	0.02	2
BXD55	0.68	0.04	7	0.83	0.04	4	0.8	0.07	4	0.89	0.05	4
BXD56	0.76	0.04	7	1.07	NA	1	0.7	NA	1	0.81	NA	1
BXD61	0.67	0.02	11	0.84	0.04	5	0.76	0.06	5	0.78	0.06	5
BXD62	0.66	0.02	7	0.95	0.01	3	0.87	0.03	3	0.82	0.06	3
BXD64	0.8	0.03	6	0.83	0.06	3	0.86	0.02	3	0.95	0.03	3
BXD66	0.75	0.03	6	1.02	0.04	3	0.75	0.04	3	0.8	0.04	3
BXD68	0.65	0.03	8	0.75	0.02	5	0.73	0.03	5	0.75	0.02	5
BXD69	0.91	NA	1	NA	NA	NA	NA	NA	NA	NA	NA	NA
BXD70	0.74	0.03	7	0.91	0.03	3	0.79	0.03	3	0.79	0.05	3
BXD71	0.78	0.02	2	0.97	NA	1	0.9	NA	1	0.63	NA	1
BXD73	0.72	0.04	8	0.76	0.04	4	0.71	0.02	4	0.79	0.04	4
BXD74	0.69	0.01	4	0.93	0	2	0.82	0.12	2	0.73	0.09	2
BXD75	0.76	0.04	9	0.94	0.03	6	0.83	0.05	6	0.83	0.05	5
BXD79	0.78	0.05	7	0.78	0.01	4	0.8	0.03	4	0.76	0.03	4
BXD84	0.71	0.03	9	0.8	0.06	5	0.73	0.04	5	0.74	0.03	5
BXD85	0.84	0.11	3	0.93	0.05	2	0.96	0.11	2	0.89	0.12	2
BXD86	0.82	0.05	4	1.12	NA	1	NA	NA	NA	NA	NA	NA
BXD87	0.63	0.02	6	0.98	0.09	3	0.85	0.03	3	1.03	0.06	3
BXH-19/TyJ	0.76	0.05	4	0.78	0.11	3	0.68	0.06	3	0.71	0.04	3
BXH-6/TyJ	0.74	0.03	9	0.77	0.07	3	0.7	0.05	5	0.69	0.05	5
BXH-9/TyJ	0.8	0.08	3	0.73	0.02	2	0.86	0.02	2	0.74	0.19	2
BXHA1	0.75	NA	1	0.9	NA	1	0.86	NA	1	0.79	NA	1
BXHB2	0.71	0.04	9	0.81	0.06	5	0.82	0.05	5	0.77	0.02	5
C3H/HeJ	0.66	0.03	12	0.8	0.04	7	0.66	0.07	5	0.7	0.04	4
C57BL/6J	0.65	0.02	17	0.72	0.03	7	0.72	0.04	6	0.78	0.09	5
C57BLKS/J	0.64	0.03	8	0.7	0.04	2	0.77	0.02	2	0.79	0.11	2
C57L/J	0.83	0.1	5	0.98	0.1	3	0.83	0.13	3	0.8	0.1	3
C58/J	0.6	0.04	6	0.68	0.03	3	0.88	0	2	0.76	0.05	2
CBA/J	0.68	0.04	9	0.66	0.04	7	0.8	0.03	5	0.7	0.04	4
CE/J	0.75	0.03	3	0.78	0.03	2	0.82	0.08	2	0.8	0.02	2
CXB-11/HiAJ	0.81	0.06	5	0.72	0.05	2	0.78	0.01	2	0.88	0.07	2
CXB-12/HiAJ	0.68	0.02	10	0.82	0.03	4	0.71	0.06	4	0.71	0.05	4
CXB-13/HiAJ	0.61	0.02	3	0.82	NA	1	0.79	NA	1	0.79	NA	1
CXB-3/ByJ	0.83	0.03	7	0.76	0.03	3	0.72	0.03	3	0.89	0.07	3
CXB-6/ByJ	0.68	0.03	10	0.79	0.03	3	0.74	0.07	3	0.74	0.05	3
CXB-7/ByJ	0.73	0.05	5	0.94	0.03	2	0.89	0.15	2	0.84	0.02	2
CXBH	0.72	0.05	7	0.84	0.03	4	0.72	0.07	4	0.8	0.03	4
DBA/2J	0.68	0.04	17	0.77	0.1	8	0.75	0.04	7	0.82	0.05	6
FVB/NJ	0.74	0.03	16	0.78	0.03	9	0.84	0.08	7	0.73	0.04	6
KK/HIJ	0.86	0.03	6	1.06	0.08	4	0.97	0	4	0.91	0.04	4
LG/J	0.83	0.04	8	0.98	0.05	4	0.84	0.06	4	0.92	0.23	3
LP/J	0.69	0.05	6	0.92	0.02	3	0.81	0.08	3	0.82	0.1	3

MA/MyJ	0.84	0.03	3	0.94	0.04	2	1.25	0.02	2	0.92	0.04	2
NOD/LtJ	0.68	0.04	10	0.75	0.04	7	0.75	0.06	5	0.77	0.12	3
NON/LtJ	0.83	0.03	10	0.98	0.03	5	0.84	0.07	5	0.73	0.03	5
NZB/BINJ	0.75	0.02	6	0.88	0.08	3	0.79	0.03	3	0.74	0.03	3
NZW/LacJ	0.79	0.02	9	1.01	0.11	2	1.02	0.05	2	0.9	NA	1
PL/J	0.65	0.02	8	0.68	0.1	4	0.7	0.08	3	0.52	0.03	2
RIIIS/J	0.66	0.02	13	0.65	0.04	6	0.62	0.07	4	0.75	0.05	4
SEA/GnJ	0.76	0.03	12	0.84	0.04	4	0.94	0.1	4	0.91	0.04	4
SJL/J	0.64	0.03	8	0.66	0.05	4	0.62	0.07	3	0.56	0.04	2
SM/J	0.69	0.02	6	0.96	0.11	3	0.82	0.01	3	0.74	0.04	3
SWR/J	0.75	0.02	13	0.87	0.07	4	0.84	0.07	4	0.8	0.07	4

B. LVIDd (mm)

Strain	m.0	se.0	n.0	m.1	se.1	n.1	m.2	se.2	n.2	m.3	se.3	n.3
129X1/SvJ	3.71	0.07	11	3.61	0.11	7	3.99	0.19	5	4.29	0.26	4
A/J	3.79	0.16	7	3.92	0.09	3	4.25	0.09	2	4.21	NA	1
AKR/J	3.69	0.11	8	3.76	0.15	6	4.17	0.13	4	4.21	0.27	2
AXB-10/PgnJ	3.79	0.05	5	3.88	NA	1	3.96	NA	1	4.23	0.04	3
AXB-12/PgnJ	3.6	0.16	3	NA	NA	NA	NA	NA	NA	NA	NA	NA
AXB-13/PgnJ	3.42	NA	1	NA	NA	NA	NA	NA	NA	NA	NA	NA
AXB-18/PgnJ	3.99	0.07	8	4.03	0.08	4	4	0.15	3	4.15	0.17	3
AXB-19/PgnJ	3.84	0.09	10	4.21	0.17	3	4.26	0.09	3	4.36	0.1	3
AXB-20/PgnJ	3.74	0.15	5	3.8	0.03	2	4.24	0.17	2	4.24	0.23	2
AXB-4/PgnJ	3.55	0.14	3	3.68	0.07	2	4.2	0.12	2	3.8	0	2
AXB-6/PgnJ	4	0.17	2	4.28	NA	1	4.33	NA	1	4.05	NA	1
AXB-8/PgnJ	3.62	0.05	8	4.01	0.19	5	4.05	0.04	5	3.8	0.08	5
BALB/cByJ	3.66	0.06	6	3.71	0.07	3	3.85	0.29	3	3.87	0.18	3
BALB/cJ	3.86	0.07	18	4.12	0.14	6	4.21	0.14	4	4.2	0.41	3
BTBRT<+>tf/J	4.06	0.09	17	4.66	0.54	2	5	0.08	2	4.97	0.14	2
BUB/BnJ	3.43	0.09	11	3.65	0.29	5	3.61	0.2	4	3.93	0.2	5
BXA-1/PgnJ	3.84	0.06	5	3.87	0.03	2	4.08	0.25	2	4.1	0	2
BXA-11/PgnJ	3.01	NA	1	NA	NA	NA	NA	NA	NA	NA	NA	NA
BXA-12/PgnJ	3.08	0.14	4	2.95	NA	1	NA	NA	NA	NA	NA	NA
BXA-14/PgnJ	3.74	0.05	9	3.92	0.08	5	4.13	0.06	5	4.1	0.13	5
BXA-16/PgnJ	3.48	0.1	6	3.68	0.09	4	3.87	0.19	4	3.85	0.28	4
BXA-2/PgnJ	3.57	0.08	7	3.99	0.13	3	4.12	0.07	3	4.11	0.12	3
BXA-24/PgnJ	4.01	0.08	13	4.21	0.23	5	4.49	0.27	5	4.5	0.17	4
BXA-4/PgnJ	3.75	0.06	8	4.05	0.27	4	4.13	0.21	4	3.99	0.17	4
BXA-7/PgnJ	3.85	0.05	10	3.85	0.13	5	3.88	0.1	5	3.95	0.05	5
BXD-1/TyJ	3.64	NA	1	NA	NA	NA	NA	NA	NA	NA	NA	NA
BXD-11/TyJ	3.84	0.17	3	4.02	NA	1	3.99	NA	1	4.32	NA	1
BXD-12/TyJ	3.72	0.13	5	4.05	0.07	2	4.19	0.06	2	4.66	0.15	2
BXD-14/TyJ	3.96	0.17	4	4.45	0.09	3	4.47	0.16	3	4.46	0.32	3
BXD-15/TyJ	4.1	0.14	3	4.92	NA	1	NA	NA	NA	NA	NA	NA
BXD-19/TyJ	3.73	0.06	2	4.84	NA	1	4.67	NA	1	5.23	NA	1
BXD-21/TyJ	3.58	0.09	15	3.94	0.1	8	3.95	0.16	8	4.02	0.11	7
BXD-22/TyJ	4.1	NA	1	3.74	NA	1	4.27	NA	1	NA	NA	NA
BXD-24/TyJ	3.09	0.1	3	2.95	NA	1	4.1	NA	1	4.21	NA	1
BXD-27/TyJ	3.97	0.17	4	NA	NA	NA	NA	NA	NA	NA	NA	NA

BXD-31/TyJ	3.67	0.14	2	4.79	NA	1	NA	NA	NA	5.28	NA	1
BXD-32/TyJ	3.75	0.07	9	3.87	NA	1	4.61	NA	1	4.66	0.22	3
BXD-33/TyJ	3.39	0.25	2	NA	NA	NA	NA	NA	NA	NA	NA	NA
BXD-34/TyJ	4.1	0.07	7	NA	NA	NA	NA	NA	NA	NA	NA	NA
BXD-38/TyJ	3.65	0.1	10	3.91	0.01	2	4.3	0.18	2	4.21	0.21	2
BXD-39/TyJ	4.1	0.13	5	4.36	0.07	3	4.6	0.05	3	4.27	0	3
BXD-40/TyJ	3.73	0.06	21	4.03	0.1	6	4.24	0.1	6	4.06	0.15	5
BXD-5/TyJ	3.47	0.02	2	3.55	NA	1	3.86	NA	1	3.38	NA	1
BXD-6/TyJ	3.65	0.29	2	4.07	NA	1	4.24	NA	1	4.29	NA	1
BXD-8/TyJ	3.73	0.07	3	4.39	0.2	2	4.97	0.19	2	4.99	0.19	2
BXD43	3.26	0.15	7	3.42	0.18	5	3.45	0.09	5	3.51	0.06	5
BXD44	3.74	0.09	4	3.84	0.12	2	3.97	0.07	2	3.9	0.04	2
BXD45	3.62	0.04	7	3.8	0.16	2	3.7	0.06	2	3.81	0.05	2
BXD48	3.54	0.07	6	3.7	0.08	4	3.83	0.13	4	3.75	0.12	4
BXD49	4.08	0.08	7	4.34	0.11	3	4.13	0.11	3	3.96	0.25	3
BXD50	3.74	0.09	5	3.91	0.13	2	4.01	0.06	2	3.49	0.18	2
BXD55	3.94	0.04	7	3.7	0.15	4	3.96	0.1	4	3.72	0.25	4
BXD56	4.05	0.08	7	3.48	NA	1	4.93	NA	1	4.71	NA	1
BXD61	3.98	0.09	11	3.99	0.11	5	4.19	0.14	5	4.19	0.13	5
BXD62	3.48	0.07	7	3.59	0.06	3	3.73	0.06	3	3.85	0.19	3
BXD64	3.86	0.07	6	3.75	0.15	3	4	0.03	3	3.63	0.1	3
BXD66	3.62	0.08	6	3.76	0.02	3	4.07	0.06	3	4.42	0.1	3
BXD68	3.72	0.04	8	3.88	0.11	5	4.08	0.08	5	4.22	0.05	5
BXD69	3.15	NA	1	NA	NA	NA	NA	NA	NA	NA	NA	NA
BXD70	3.73	0.09	7	3.76	0.17	3	3.84	0.04	3	3.95	0.15	3
BXD71	3.51	0.01	2	3.6	NA	1	4.05	NA	1	4.35	NA	1
BXD73	4.01	0.11	8	4.45	0.28	4	4.54	0.12	4	4.46	0.13	4
BXD74	3.49	0.13	4	3.94	0.14	2	4.32	0.11	2	4.07	0.23	2
BXD75	3.76	0.06	9	4.26	0.13	6	4.6	0.2	6	4.38	0.11	5
BXD79	3.38	0.09	7	3.73	0.06	4	3.96	0.14	4	3.49	0.13	4
BXD84	3.74	0.09	9	3.83	0.06	5	4.25	0.12	5	3.98	0.14	5
BXD85	3.93	0.19	3	3.75	0.3	2	3.54	0.12	2	3.83	0.18	2
BXD86	3.87	0.13	4	3.08	NA	1	NA	NA	NA	NA	NA	NA
BXD87	3.69	0.06	6	3.66	0.1	3	3.95	0.02	3	3.7	0.13	3
BXH-19/TyJ	3.32	0.07	4	3.57	0.08	3	4	0.12	3	4.04	0.08	3
BXH-6/TyJ	3.58	0.11	9	3.85	0.09	3	3.95	0.13	5	3.78	0.05	5
BXH-9/TyJ	3.64	0.16	3	3.83	0	2	3.88	0.08	2	3.94	0.41	2
BXHA1	3.36	NA	1	4.1	NA	1	4.25	NA	1	4.46	NA	1
BXHB2	3.48	0.08	9	3.91	0.06	5	4.1	0.03	5	4.21	0.11	5
C3H/HeJ	3.64	0.11	12	3.81	0.15	7	4.16	0.17	5	4.31	0.24	4
C57BL/6J	3.82	0.07	17	4.02	0.09	7	4.13	0.12	6	4.13	0.09	5
C57BLKS/J	3.97	0.08	8	4.24	0.16	2	4.19	0.27	2	4.25	0.08	2
C57L/J	3.7	0.07	5	3.74	0.1	3	3.9	0.14	3	4.2	0.17	3
C58/J	3.9	0.12	6	4.18	0.05	3	3.88	0	2	4.13	0.29	2
CBA/J	3.71	0.11	9	3.85	0.11	7	4.01	0.1	5	4.16	0.16	4
CE/J	3.83	0.12	3	3.91	0.03	2	4.04	0.02	2	4.17	0.04	2
CXB-11/HiAJ	3.97	0.22	5	4.06	0.15	2	4.45	0.06	2	4.14	0.28	2
CXB-12/HiAJ	4.05	0.07	10	4.35	0.2	4	4.52	0.06	4	4.76	0.13	4
CXB-13/HiAJ	4.01	0.41	3	3.83	NA	1	4.03	NA	1	3.62	NA	1

CXB-3/ByJ	3.53	0.12	7	4.19	0.03	3	4.38	0.13	3	4.02	0.12	3
CXB-6/ByJ	3.55	0.07	10	3.84	0.14	3	4.03	0.15	3	4.13	0.18	3
CXB-7/ByJ	3.71	0.22	5	3.77	0.02	2	4.06	0.15	2	4.17	0.07	2
CXBH	3.87	0.08	7	3.93	0.19	4	4.32	0.13	4	4.34	0.23	4
DBA/2J	3.96	0.07	17	4.28	0.08	8	4.64	0.16	7	4.71	0.1	6
FVB/NJ	3.61	0.06	16	3.84	0.12	9	3.99	0.13	7	4.26	0.08	6
KK/HIJ	3.8	0.07	6	4.51	0.15	4	4.65	0.22	4	4.61	0.27	4
LG/J	3.79	0.05	8	3.5	0.25	4	3.94	0.32	4	4.17	0.3	3
LP/J	3.42	0.04	6	3.12	0.2	3	3.47	0.04	3	3.39	0.1	3
MA/MyJ	3.35	0.13	3	3.61	0.22	2	3.56	0.08	2	3.81	0.2	2
NOD/LtJ	3.82	0.14	10	3.7	0.1	7	4.07	0.06	5	4.09	0.2	3
NON/LtJ	3.77	0.11	10	3.84	0.09	5	3.92	0.12	5	4.21	0.16	5
NZB/BINJ	3.76	0.11	6	3.69	0.15	3	4.15	0.19	3	4.28	0.15	3
NZW/LacJ	4.22	0.08	9	4.17	0.08	2	4.63	0.02	2	4.61	NA	1
PL/J	3.53	0.11	8	3.6	0.16	4	3.99	0.09	3	4.12	0.39	2
RIIIS/J	3.5	0.08	13	3.85	0.08	6	4.07	0.12	4	4	0.11	4
SEA/GnJ	4.04	0.09	12	4.34	0.19	4	4.19	0.2	4	4.29	0.28	4
SJL/J	3.67	0.1	8	3.96	0.07	4	4.35	0.2	3	3.92	0.04	2
SM/J	3.47	0.03	6	3.71	0.1	3	3.92	0.06	3	4	0.07	3
SWR/J	3.62	0.1	13	3.44	0.14	4	3.72	0.13	4	3.97	0.16	4

C. LVM (mg)

Strain	m.0	se.0	n.0	m.1	se.1	n.1	m.2	se.2	n.2	m.3	se.3	n.3
129X1/SvJ	78.91	4.01	11	102.33	8.07	7	105.41	10.82	5	120.45	8.75	4
A/J	83.96	6.56	7	85.06	6.17	3	98.75	2.8	2	85.72	NA	1
AKR/J	100.32	5.29	8	101.51	7.73	6	107.91	7.73	4	124.56	22.03	2
AXB-10/PgnJ	92.69	5.16	5	138.79	NA	1	118.06	NA	1	114.53	4.17	3
AXB-12/PgnJ	87.46	1.22	3	NA	NA	NA	NA	NA	NA	NA	NA	NA
AXB-13/PgnJ	67.3	NA	1	NA	NA	NA	NA	NA	NA	NA	NA	NA
AXB-18/PgnJ	100.48	5.82	8	99.91	9.02	4	130.28	4.94	3	120.78	6.9	3
AXB-19/PgnJ	86.66	3.72	10	123.68	15.74	3	142.86	19.2	3	145.88	4.63	3
AXB-20/PgnJ	108.3	6.55	5	127.97	0.01	2	142.46	24.6	2	135.42	10.43	2
AXB-4/PgnJ	76.08	7.41	3	127.99	6.27	2	96.94	8.79	2	99.08	0	2
AXB-6/PgnJ	85.24	6.01	2	112.03	NA	1	111.6	NA	1	134.97	NA	1
AXB-8/PgnJ	84.78	3.01	8	110.82	8.45	5	101.9	11.08	5	100.25	5.21	5
BALB/cByJ	89.82	4.17	6	107.88	10.29	3	125.11	12.12	3	121.91	10.11	3
BALB/cJ	81.63	3.56	18	100.35	11.58	6	102.32	8.04	4	108.67	7.71	3
BTBRT<+>tf/J	106.29	5.54	17	156.64	17.21	2	190.12	28.18	2	199.44	31.88	2
BUB/BnJ	91.03	4.98	11	122.17	17.26	5	127.51	12.85	4	128.72	14.23	5
BXA-1/PgnJ	83.14	5.93	5	98.2	5.17	2	106.78	8.94	2	105.66	4.13	2
BXA-11/PgnJ	65.29	NA	1	NA	NA	NA	NA	NA	NA	NA	NA	NA
BXA-12/PgnJ	60.82	3.08	4	71.15	NA	1	NA	NA	NA	NA	NA	NA
BXA-14/PgnJ	82.29	4.23	9	133.64	11.43	5	136.61	4.16	5	143.8	9.77	5
BXA-16/PgnJ	69.14	3.28	6	107.99	19.35	4	92.45	6.51	4	106.4	20.68	4
BXA-2/PgnJ	85.59	6.71	7	111.11	2.96	3	128.28	14.87	3	128.52	9.75	3
BXA-24/PgnJ	95.34	4.37	13	154.35	21.71	5	151.83	10.74	5	174.4	24.12	4
BXA-4/PgnJ	85.16	3.02	8	100.9	2.16	4	132.45	4.69	4	136.09	5.83	4
BXA-7/PgnJ	82.37	4.08	10	100.82	6.58	5	114.77	4.1	5	107.2	8.91	5
BXD-1/TyJ	106.84	NA	1	NA	NA	NA	NA	NA	NA	NA	NA	NA
BXD-11/TyJ	87.43	12.19	3	140.72	NA	1	140.64	NA	1	105.73	NA	1
BXD-12/TyJ	88.34	10.26	5	118.61	9.14	2	117.78	1.49	2	105.3	20.67	2
BXD-14/TyJ	107.94	7.72	4	165.12	9.23	3	207.52	15	3	194.61	8.64	3
BXD-15/TyJ	96.52	8.41	3	175.29	NA	1	NA	NA	NA	NA	NA	NA
BXD-19/TyJ	95.13	2.8	2	177.57	NA	1	131.82	NA	1	194.31	NA	1
BXD-21/TyJ	93.82	4.94	15	126.67	7.45	8	125.17	7.88	8	127.91	7.2	7

BXD-22/TyJ	117.95	NA	1	129.06	NA	1	65.74	NA	1	NA	NA	NA
BXD-24/TyJ	73.15	5.17	3	118.89	NA	1	96.72	NA	1	90.77	NA	1
BXD-27/TyJ	102.23	7.19	4	NA	NA	NA	NA	NA	NA	NA	NA	NA
BXD-31/TyJ	84.34	5.23	2	160	NA	1	NA	NA	NA	201.62	NA	1
BXD-32/TyJ	75.23	4.34	9	166.54	NA	1	145.49	NA	1	160.24	17.16	3
BXD-33/TyJ	62.96	9.85	2	NA	NA	NA	NA	NA	NA	NA	NA	NA
BXD-34/TyJ	115.38	7.3	7	NA	NA	NA	NA	NA	NA	NA	NA	NA
BXD-38/TyJ	93.56	4.15	10	130.75	3.78	2	117	32.97	2	135.08	14.35	2
BXD-39/TyJ	106.61	10.25	5	132.11	8.1	3	120.84	16.17	3	133.69	13.53	3
BXD-40/TyJ	82.98	2.1	21	110.91	8.56	6	148.61	27.28	6	117.14	5	5
BXD-5/TyJ	85.99	11.44	2	119.47	NA	1	135.8	NA	1	108.02	NA	1
BXD-6/TyJ	98.87	3.7	2	88.16	NA	1	116.04	NA	1	130.27	NA	1
BXD-8/TyJ	99.78	3.58	3	174.13	19.37	2	168.64	3.45	2	178.12	23.08	2
BXD43	72.13	2.68	7	103.21	4.17	5	104.95	2.73	5	97.97	5.5	5
BXD44	84.19	2.27	4	107.87	2.25	2	106.37	6.25	2	122.22	5.26	2
BXD45	94.24	2.2	7	117.64	2.37	2	127.31	13.69	2	142.32	2.09	2
BXD48	91.2	2.77	6	142	13.41	4	125.35	4.2	4	131.66	5.72	4
BXD49	103.86	4.21	7	143.54	9.46	3	124.08	15.48	3	126.26	7.77	3
BXD50	79.91	2.62	5	114.78	13.28	2	124.85	4.82	2	110.21	15.08	2
BXD55	93.77	3.65	7	101.81	8.56	4	116.25	10.68	4	105.18	11.42	4
BXD56	116.28	6.45	7	143.63	NA	1	149.57	NA	1	168.3	NA	1
BXD61	92.43	3.59	11	119.28	7.99	5	124.54	6.25	5	125.17	4.19	5
BXD62	73.25	2.54	7	119.3	2.28	3	116.14	1.52	3	110.79	2.64	3
BXD64	105.25	0.72	6	123.92	1.38	3	138.56	6.9	3	128.04	12.04	3
BXD66	89.89	4.1	6	131.18	3.56	3	111.68	6.7	3	133.69	3.96	3
BXD68	81.67	2.6	8	103.34	2.61	5	112.59	4.95	5	116.43	3.33	5
BXD69	90.73	NA	1	NA	NA	NA	NA	NA	NA	NA	NA	NA
BXD70	92.96	3.78	7	125.26	6.29	3	113.19	8.74	3	114.92	3.46	3
BXD71	83.11	0.32	2	120.36	NA	1	130.63	NA	1	94.62	NA	1
BXD73	103.77	2.37	8	126.57	5.17	4	127.4	2.53	4	135.22	9.63	4
BXD74	80	2.89	4	136.83	5.5	2	122.75	25.56	2	107.53	0.81	2
BXD75	94.99	4.72	9	147.49	7.89	6	163.32	24.52	6	139.65	4.81	5
BXD79	80.74	5.38	7	106.17	5.34	4	111.11	8.08	4	92.53	8.08	4
BXD84	89.26	4.45	9	111.06	6.13	5	115.9	8.34	5	108.86	8.15	5
BXD85	98.58	9.59	3	124.15	7.92	2	123.69	8.7	2	125.45	3.03	2
BXD86	116.36	6.23	4	123.56	NA	1	NA	NA	NA	NA	NA	NA
BXD87	82.91	3.53	6	138.48	3.54	3	126.03	2.44	3	136.42	2.36	3
BXH-19/TyJ	74.48	5.8	4	92.07	12.54	3	87.57	1.76	3	97.35	9.18	3
BXH-6/TyJ	85.85	5.22	9	93.92	11.98	3	100.85	9.23	5	90.94	7.52	5
BXH-9/TyJ	94.08	10.27	3	103.39	4.34	2	108.98	2.51	2	108.19	14.9	2
BXHA1	85.25	NA	1	139.25	NA	1	146.13	NA	1	138.74	NA	1
BXHB2	80.6	4.63	9	110.1	5.23	5	124.19	11.67	5	119.32	5.33	5
C3H/HeJ	82.21	3.67	12	106.02	7.18	7	102.92	11.67	5	108.06	5.33	4
C57BL/6J	82.68	2.65	17	109.1	6.41	7	114.75	7.71	6	116.03	8.95	5
C57BLKS/J	88.26	2.3	8	104.7	13.15	2	116.44	17.58	2	123.08	28.8	2
C57L/J	93.94	8.17	5	130.09	14.58	3	120.55	20.38	3	137.43	29.26	3
C58/J	74.21	3.86	6	104.91	2.63	3	112.22	11.25	2	135.07	7.82	2
CBA/J	83.34	7.59	9	93.91	9.12	7	112.93	4.5	5	116.5	5.03	4
CE/J	89.77	2.91	3	115.17	5.67	2	129.59	1.8	2	135.48	5.47	2
CXB-11/HiAJ	110.55	6.29	5	105.07	3.89	2	129.6	4.91	2	134.84	18.41	2
CXB-12/HiAJ	98.23	4.31	10	123.44	8.24	4	118.88	7.76	4	149.22	13.21	4
CXB-13/HiAJ	86.33	18.11	3	103.96	NA	1	110.69	NA	1	109.18	NA	1
CXB-3/ByJ	94.15	4.8	7	117.72	8.57	3	114.43	3.26	3	134.92	2.05	3
CXB-6/ByJ	79.14	3.15	10	107	6.59	3	114.42	10.4	3	115.92	3.29	3
CXB-7/ByJ	88	4.24	5	118.46	7.57	2	128.56	22.01	2	134.74	0.25	2
CXBH	98.64	7.69	7	121.85	5.98	4	120.71	7.86	4	132.87	5.2	4
DBA/2J	90.55	5.4	17	126.97	18.09	8	141.76	11.93	7	166.41	14.34	6
FVB/NJ	93.36	2.96	15	114.56	5.71	9	132.43	8.92	7	113.43	6.42	6
KK/HlJ	117.52	3.26	6	185.1	7.33	4	185.43	10.72	4	167.76	13.2	4

LG/J	110.72	4.57	8	130.02	12.92	4	122.89	20.05	4	131.47	19.99	3
LP/J	65.91	5.2	6	86.67	8.32	3	89.59	7.88	3	91.61	12.95	3
MA/MyJ	91.71	3.53	3	130.32	5.69	2	172.34	15.4	2	144.48	19.49	2
NOD/LtJ	87.14	2.67	10	98.52	5.12	7	114.73	13.6	5	120.43	12.41	3
NON/LtJ	106.08	3.28	10	130.87	6.42	5	122.36	7.82	5	115.97	2.67	5
NZB/BlNJ	104.35	3.93	6	115.08	10.38	3	129.54	2.24	3	128.25	4.08	3
NZW/LacJ	120.36	2.52	9	183.51	17.9	2	195.03	14.72	2	163.56	NA	1
PL/J	80.11	6.14	8	88.35	8.28	4	95.52	13.21	3	90.69	4.88	2
RIIIS/J	71.91	3.19	13	81.77	3.28	6	95.2	12.29	4	103.53	5.42	4
SEA/GnJ	110.8	4.22	12	148.87	14.17	4	165.05	22.85	4	171.05	20.42	4
SJL/J	71.65	5.28	8	91.93	7.73	4	96.09	10.41	3	75.8	0.87	2
SM/J	80.71	2.3	6	117.11	4.58	3	120.32	5.31	3	110.88	1.04	3
SWR/J	89.19	3.82	13	108.01	2.86	4	121.48	14.2	4	116.02	14.58	4

D. FS (%)

Strain	m.0	se.0	n.0	m.1	se.1	n.1	m.2	se.2	n.2	m.3	se.3	n.3
129X1/SvJ	33.33	1.94	11	35.43	3.37	7	40.97	5.85	5	35.91	5.37	4
A/J	33.85	1.26	7	28.47	2.78	3	30.07	7.43	2	21.43	NA	1
AKR/J	37.55	1.64	8	44.81	1.45	6	31.12	5.76	4	33.34	3.47	2
AXB-10/PgnJ	33.37	2.28	5	42.6	NA	1	43.82	NA	1	29.63	4.74	3
AXB-12/PgnJ	36.7	3.19	3	NA	NA	NA	NA	NA	NA	NA	NA	NA
AXB-13/PgnJ	29.6	NA	1	NA	NA	NA	NA	NA	NA	NA	NA	NA
AXB-18/PgnJ	34.39	1.9	8	33.27	3.72	4	37.21	2.47	3	34.74	5.49	3
AXB-19/PgnJ	34.64	1.66	10	32.62	5.11	3	34.51	4.99	3	33.73	5.98	3
AXB-20/PgnJ	41.14	3.75	5	33.52	4.58	2	34.93	10.71	2	20.45	0.04	2
AXB-4/PgnJ	37.49	5.06	3	44.36	5.29	2	30.71	2.86	2	40.28	7.19	2
AXB-6/PgnJ	29.66	3.23	2	38.83	NA	1	40.14	NA	1	34.49	NA	1
AXB-8/PgnJ	35.2	2.08	8	39.4	2.97	5	31.57	2.67	5	35.79	2.13	5
BALB/cByJ	42.56	1.76	6	48.28	0.78	3	42.89	1.37	3	46.74	1.86	3
BALB/cJ	31.73	1.35	18	31.04	4	6	31.77	4.98	4	32.19	7.59	3
BTBRT<+>tf/J	35.43	1.52	17	33.63	3.46	2	33.63	0.74	2	26.44	3.6	2
BUB/BnJ	47.77	1.7	11	44.3	6.48	5	49.92	3.99	4	48.58	4.38	5
BXA-1/PgnJ	33.26	1.66	5	42.33	0.53	2	35.54	2.32	2	37.8	0.87	2
BXA-11/PgnJ	48.28	NA	1	NA	NA	NA	NA	NA	NA	NA	NA	NA
BXA-12/PgnJ	43.16	6.06	4	37.04	NA	1	NA	NA	NA	NA	NA	NA
BXA-14/PgnJ	30.45	1.59	9	37.01	2.43	5	34.88	2.6	5	38.32	1.5	5
BXA-16/PgnJ	38.2	2.8	6	41.27	7.79	4	30.76	5.38	4	34.1	5.44	4
BXA-2/PgnJ	36.69	2.54	7	37.13	2.26	3	40.27	1.75	3	43.98	3.85	3
BXA-24/PgnJ	27.13	1.56	13	36.75	3.64	5	36.51	5.88	5	33.07	1.58	4
BXA-4/PgnJ	32.31	1.1	8	30.17	5.59	4	38.93	0.69	4	43.36	2.53	4
BXA-7/PgnJ	32.37	0.94	10	38.5	3.9	5	38.86	1.18	5	33.27	3.73	5
BXD-1/TyJ	38.82	NA	1	NA	NA	NA	NA	NA	NA	NA	NA	NA
BXD-11/TyJ	30.08	2.72	3	40.47	NA	1	37.67	NA	1	29.75	NA	1
BXD-12/TyJ	41.49	4.94	5	41.68	0.62	2	44.88	2.14	2	36.63	0.87	2
BXD-14/TyJ	31.57	2.97	4	39.48	1.43	3	37.75	5.62	3	45.39	5.82	3
BXD-15/TyJ	31.07	5.51	3	34.44	NA	1	NA	NA	NA	NA	NA	NA
BXD-19/TyJ	43.37	8.12	2	29.94	NA	1	11.54	NA	1	17.42	NA	1
BXD-21/TyJ	42.97	2.75	15	41.28	0.6	8	40.79	2.4	8	41.31	2.2	7
BXD-22/TyJ	32.67	NA	1	54.04	NA	1	19.23	NA	1	NA	NA	NA
BXD-24/TyJ	43.83	9.74	3	60.05	NA	1	28	NA	1	26.62	NA	1
BXD-27/TyJ	39.8	3.44	4	NA	NA	NA	NA	NA	NA	NA	NA	NA

BXD-31/TyJ	42.44	5.62	2	24	NA	1	NA	NA	NA	27.46	NA	1
BXD-32/TyJ	31.51	2.65	9	37.85	NA	1	31.76	NA	1	29.5	5.36	3
BXD-33/TyJ	35.91	5.83	2	NA	NA	NA	NA	NA	NA	NA	NA	NA
BXD-34/TyJ	28.25	1.95	7	NA	NA	NA	NA	NA	NA	NA	NA	NA
BXD-38/TyJ	39.16	3.24	10	45.72	0.1	2	33.98	0.46	2	36.38	3.79	2
BXD-39/TyJ	37.76	2.6	5	39.91	2.55	3	39.44	2.95	3	43.27	2.13	3
BXD-40/TyJ	35.39	1.76	21	36.04	4.41	6	32.99	3.71	6	38.85	5.56	5
BXD-5/TyJ	39.05	0.95	2	39.23	NA	1	39.72	NA	1	46.03	NA	1
BXD-6/TyJ	49.58	6.48	2	42.28	NA	1	43.23	NA	1	53.5	NA	1
BXD-8/TyJ	36.28	3.99	3	32.28	1.82	2	27	4.36	2	32.73	1.62	2
BXD43	40.22	4.63	7	44.12	3.01	5	49.35	2.43	5	48.79	3.94	5
BXD44	37.62	1.64	4	44.18	5.94	2	45.52	4.66	2	41.09	3.59	2
BXD45	37.46	1.27	7	44.15	0.11	2	42.11	2.25	2	42.32	3.07	2
BXD48	37.87	2.46	6	47.96	2.13	4	51.64	1.25	4	49.54	2.43	4
BXD49	34.68	1.37	7	44.15	1.6	3	43.42	3.77	3	42.79	2.51	3
BXD50	36.2	2.81	5	46.6	2.16	2	46.27	5.62	2	54.38	6.98	2
BXD55	34.63	1.63	7	42.68	3.66	4	44.91	1.32	4	47.25	2.98	4
BXD56	33.72	1.67	7	38.82	NA	1	22.11	NA	1	25.06	NA	1
BXD61	30.96	1.71	11	39.14	1.98	5	39.37	3.41	5	43.36	1.52	5
BXD62	41.5	1.95	7	46.54	0.85	3	49.48	2.38	3	53.75	3.76	3
BXD64	34.31	1.67	6	46.84	4.81	3	46.08	5.99	3	50.78	6.49	3
BXD66	47.97	4.71	6	41.17	3.94	3	45.62	1.31	3	32.33	1.08	3
BXD68	32.96	1.81	8	42.26	3.01	5	34.33	3	5	29.14	1.54	5
BXD69	47.85	NA	1	NA	NA	NA	NA	NA	NA	NA	NA	NA
BXD70	38.2	3.15	7	44.57	4.28	3	35.24	2.17	3	40.92	2.59	3
BXD71	40.7	1.13	2	53.23	NA	1	39.19	NA	1	32.08	NA	1
BXD73	38.51	1.45	8	35.94	3.45	4	34.11	3.4	4	39.53	4.7	4
BXD74	45.54	5.98	4	48.29	1.98	2	35.81	1.14	2	40.49	1.13	2
BXD75	30.17	2.01	9	36.93	1.93	6	34.15	2.87	6	38.98	1.64	5
BXD79	43.27	2.86	7	43.53	1.73	4	41.39	4.82	4	50.91	4.66	4
BXD84	40.09	1.93	9	47.89	1.95	5	34.56	2	5	40.14	4.14	5
BXD85	32.77	4.64	3	47.99	6.77	2	46.12	5.08	2	52.64	4.04	2
BXD86	35.03	1.77	4	55.41	NA	1	NA	NA	NA	NA	NA	NA
BXD87	33.34	2.95	6	58.28	2.05	3	42.45	4.48	3	53.02	3.21	3
BXH-19/TyJ	41.13	2.01	4	45.25	1.04	3	34.66	1.82	3	37.27	5.29	3
BXH-6/TyJ	37.8	1.93	9	37.55	2.78	3	40.71	3.32	5	43.25	2.69	5
BXH-9/TyJ	37.74	2.79	3	38.39	9.46	2	29.19	1.85	2	32.11	8.21	2
BXHA1	45.53	NA	1	47.12	NA	1	37.27	NA	1	28.22	NA	1
BXHB2	43.54	1.71	9	30.18	3.44	5	36.82	5.15	5	32.92	3.74	5
C3H/HeJ	36.55	1.95	12	35.34	3.47	7	28.81	2.02	5	28.11	3.64	4
C57BL/6J	34.38	1.15	17	37.59	2.51	7	32.03	4.5	6	34.67	3.04	5
C57BLKS/J	33.09	0.89	8	35.33	4.9	2	38.09	5.27	2	34.93	0.52	2
C57L/J	36.59	3.4	5	44.07	5.18	3	33.99	5.07	3	35.44	5.11	3
C58/J	34.3	3.08	6	46.33	3.64	3	39.17	4.66	2	37.32	0.44	2
CBA/J	33.58	1.75	9	38.15	0.94	7	36.55	2.46	5	36.21	1.1	4
CE/J	33.66	2.08	3	44.83	1.42	2	42.31	0.55	2	42.08	1.17	2
CXB-11/HiAJ	37.1	1.98	5	35.91	3.05	2	36.34	8.06	2	39.41	0.1	2
CXB-12/HiAJ	27.9	1.58	10	36.45	2.39	4	31.08	2.53	4	30.17	1.16	4
CXB-13/HiAJ	37.78	4.73	3	47.16	NA	1	38.98	NA	1	38.52	NA	1

CXB-3/ByJ	41.72	1.71	7	29.76	3.84	3	26.7	1.55	3	33.38	2.14	3
CXB-6/ByJ	40.21	1.78	10	46.84	3.55	3	40.33	2.88	3	35.32	4.13	3
CXB-7/ByJ	31.11	3.38	5	40.81	3.59	2	29	1.07	2	30.88	3.55	2
CXBH	32.18	1.15	7	32.56	2.38	4	27.87	3.01	4	35.41	4.11	4
DBA/2J	30.14	2.08	17	33.63	2.93	8	34.75	1.94	7	32.4	1.43	6
FVB/NJ	41.45	1.81	16	45.18	2.06	9	41.73	2.23	7	33.6	1.12	6
KK/HIJ	44.35	2.69	6	29.82	2.77	4	31.04	1.51	4	31.25	2.97	4
LG/J	37.56	1.41	8	48.81	2.95	4	36.25	2.56	4	42.9	7.44	3
LP/J	30.12	2.04	6	46.05	1.51	3	41.07	3.8	3	47.06	5.89	3
MA/MyJ	36.86	2.22	3	52.87	3.58	2	47.95	11.11	2	37.84	3.83	2
NOD/LtJ	30.99	1.55	10	37.63	1.67	7	26.24	3.17	5	37.66	4.89	3
NON/LtJ	41.59	1.53	10	36.76	1.19	5	37.54	2.06	5	36.15	2.6	5
NZB/BINJ	40.29	2.97	6	55.98	2.08	3	44.74	5.59	3	46.19	4.48	3
NZW/LacJ	30.68	0.97	9	54.65	6.02	2	42.57	3.75	2	39.43	NA	1
PL/J	30.39	2.33	8	36.77	3.21	4	34.42	3.28	3	39.98	1.2	2
RIIIS/J	38.96	1.98	13	33.58	2.32	6	38.75	0.91	4	33.46	2.78	4
SEA/GnJ	30.95	2.28	12	38.97	4.19	4	48.95	3.85	4	45.69	3.64	4
SJL/J	38.83	2.83	8	37.85	0.88	4	33.15	3.25	3	23.34	0.8	2
SM/J	38.55	2.26	6	39.77	4.19	3	43.47	4.37	3	39.47	2.09	3
SWR/J	39.54	1.37	13	42.52	2.91	4	45.6	2.84	4	37.92	1	4

Abbreviations: Interventricular septal wall thickness at end diastole (IVSd); left ventricular internal dimension at end diastole (LVIDd); left ventricular mass (LVM); fractional shortening (FS). Strain means (m), standard errors of the means (se), and sample sizes (n) at each of the baseline, week 1, week 2, and week 3 time points for IVSd (A), LVIDd (B), LVM (C) and FS (D) are reported. Suffixes represent weekly echocardiographic time points under control (.0 = baseline) and isoproterenol (.1 = week 1, .2 = week 2, .3 = week 3) conditions.
