# Supplementary material for: Genetic Dissection of Cardiac Remodeling in an Isoproterenol-Induced Heart Failure Mouse Model
Source: PLoS Genet. 2016 Jul 6;12(7):e1006038. doi: 10.1371/journal.pgen.1006038 (PMC4934852; doi:10.1371/journal.pgen.1006038)
Supplement: S8 Table — (PDF) [file pgen.1006038.s019.pdf]

S8 Table. Human and mouse cardiovascular GWAS overlap genes.
The association loci were compared to cardiovascular GWAS loci in humans to identify genes in the overlapping regions.

mouse	chr	start	end	analyses	human	trait	author	rsID
Tusc1	4	93000839	93002202	wt_delta_mouse_covar_RV	TUSC1	RR interval (heart rate)	Marroni F	rs12552736 rs13300284
Fam46a	9	85214046	85220731	w1_delta_mouse_covar_IVSd	FAM46A	Blood pressure	Franceschini N	rs6924906
Clstn2	9	97344814	97933600	wt_delta_mouse_covar_RV	CLSTN2	Sudden cardiac arrest	Aouizerat BE	rs11708189
Crhr1	11	103994169	104036837	wt_delta_mouse_covar_RV	CRHR1	Idiopathic pulmonary fibrosis	Noth I	rs17690703
Lrp12	15	39702149	39775540	w1_echo_mouse_covar_FS	LRP12	Sudden cardiac arrest	Aouizerat BE	rs16872085
Zfpm2	15	40486588	40936138	w1_echo_mouse_covar_FS	ZFPM2	Sudden cardiac arrest	Aouizerat BE	rs16872085
Slc8a1	17	81785359	82048947	wt_iso_mouse_covar_RV	SLC8A1	QT interval	Kim JW	rs13017846
Col17a1	19	47720834	47766584	wt_delta_mouse_covar_RV	COL17A1	Cardiac hypertrophy	Parsa A	rs1320448
