# Supplementary material for: Genetic Dissection of Cardiac Remodeling in an Isoproterenol-Induced Heart Failure Mouse Model
Source: PLoS Genet. 2016 Jul 6;12(7):e1006038. doi: 10.1371/journal.pgen.1006038 (PMC4934852; doi:10.1371/journal.pgen.1006038)
Supplement: S9 Table — (PDF) [file pgen.1006038.s020.pdf]

S9 Table. *Myh14* cis-eQTL across multiples tissues in different HMDP datasets.

Dataset	ProbesetID	pvalue	OR
AthAoFemale	1428835_PM_at	0.006	0.91
AthLiverFemale	1428835_PM_at	0.0002	0.89
ChowAdipose	1428835_at	0.004	1.13
ChowAorta	1428835_PM_at	0.006	1.16
ChowBone	ILMN_1217519	0.03	1.01
ChowHeart*	1428835_at	0.0005	1.35
ChowLiver	1428835_at	6.12E-06	1.19
HFAdiposeFemale	1428835_at	0.0004	1.22
HFAdiposeMale	1428835_at	0.004	1.18
HFHypothalMale	TSS82697	3.7E-09	0.79
HFLiverFemale	1428835_at	2.99E-11	1.23
HFLiverMale	1428835_at	2.08E-12	1.08

* Associated SNP outside of the LD block of rs40560913
