# Supplementary material for: Genetic Dissection of Cardiac Remodeling in an Isoproterenol-Induced Heart Failure Mouse Model
Source: PLoS Genet. 2016 Jul 6;12(7):e1006038. doi: 10.1371/journal.pgen.1006038 (PMC4934852; doi:10.1371/journal.pgen.1006038)
Supplement: S10 Table — (PDF) [file pgen.1006038.s021.pdf]

S10 Table. ***Myh14*** **correlation to heart weight in different HMDP datasets.**

Trait	Tissue	bicor	pvalue
Heart/NMR_Mass_8wks	HFAdiposeFemale	-0.27	9.31E-05
Heart/NMR_Mass_8wks	HFLiverMale	-0.19	0.003
Heart/NMR_Mass_8wks	HFLiverFemale	-0.15	0.033
